# Is emergency doctors’ tolerance of clinical uncertainty on a novel measure associated with doctor well-being, healthcare resource use and patient outcomes?

**DOI:** 10.1136/emermed-2023-213256

**Published:** 2024-11-27

**Authors:** Luke Budworth, Brad Wilson, Joanna Sutton-Klein, Subhashis Basu, Colin O'Keeffe, Suzanne M Mason, Andrew Ang, Sally Anne-Wilson, Kevin Reynard, Susan Croft, Anoop D Shah, Sakarias Bank, Mark Conner, Rebecca Lawton

**Affiliations:** 1 Bradford Institute for Health Research, Bradford, UK; 2 The University of Sheffield School of Health and Related Research, Sheffield, UK; 3 ScHARR, University of Sheffield, Sheffield, UK; 4 Barnsley District General Hospital, Barnsley, Barnsley, UK; 5 Airedale General Hospital, Keighley, West Yorkshire, UK; 6 Leeds Teaching Hospitals NHS Trust, Leeds, UK; 7 University College London Institute of Health Informatics, London, UK; 8 University of Skövde School of Health Sciences, Skovde, Västra Götaland, Sweden; 9 University of Leeds School of Psychology, Leeds, Leeds, UK; 10 University of Leeds Institute of Psychological Sciences, Leeds, Leeds, UK

**Keywords:** cost efficiency, risk management, psychology, safety, staff support

## Abstract

**Introduction:**

Emergency doctors routinely face uncertainty—they work with limited patient information, under tight time constraints and receive minimal post-discharge feedback. While higher uncertainty tolerance (UT) among staff is linked with reduced resource use and improved well-being in various specialties, its impact in emergency settings is underexplored. We aimed to develop a UT measure and assess associations with doctor-related factors (eg, experience), patient outcomes (eg, reattendance) and resource use (eg, episode costs).

**Methods:**

From May 2021 to February 2022, emergency doctors (specialty trainee 3 and above) from five Yorkshire (UK) departments completed an online questionnaire. This included a novel UT measure—an adapted Physicians’ Reaction to Uncertainty scale collaboratively modified within our team according to Hillen *et al*’s (2017) UT model. The questionnaire also included well-being-related measures (eg, Brief Resilience Scale) and assessed factors like doctors’ seniority. Patient encounters involving prespecified ‘uncertainty-inducing’ problems (eg, headache) were analysed. Multilevel regression explored associations between doctor-level factors, resource use and patient outcomes.

**Results:**

39 doctors were matched with 384 patients. The UT measure demonstrated high reliability (Cronbach’s *α*=0.92) and higher UT was significantly associated with better psychological well-being including greater resilience (Pearson’s r=0.56; 95% CI=0.30 to 0.74) and lower burnout (eg, Cohen’s d=−2.98; −4.62 to −1.33; mean UT difference for ‘no’ vs ‘moderate/high’ burnout). UT was not significantly associated with resource use (eg, episode costs: β=−0.07; −0.32 to 0.18) or patient outcomes including 30-day readmission (eg, OR=0.82; 0.28 to 2.35).

**Conclusions:**

We developed a reliable UT measure for emergency medicine. While higher UT was linked to doctor well-being, its impact on resource use and patient outcomes remains unclear. Further measure validation and additional research including intervention trials are necessary to confirm these findings and explore the implications of UT in emergency practice.

**WHAT IS ALREADY KNOWN ON THIS TOPIC**

- Emergency medicine demands rapid decision-making with limited information where doctors’ tolerance for uncertainty may affect resource utilisation and clinical decisions.

**WHAT THIS STUDY ADDS**

- We introduce a measure of uncertainty tolerance (UT) showing significant associations with improved doctor well-being but inconclusive results for resource use and patient outcomes.

**HOW THIS STUDY MIGHT AFFECT RESEARCH, PRACTICE OR POLICY**

- Enhancing UT could improve doctor well-being suggesting potential benefits from integrating tolerance training in emergency departments. Further research is needed to replicate findings related to resource use and patient outcomes.

## Background

 Emergency doctors routinely face uncertainty; they work with limited patient information under tight time constraints and receive minimal postdischarge feedback. Indeed, to do their jobs they need to tolerate this uncertainty. Uncertainty tolerance (UT) means adaptively balancing thoughts, feelings and actions in uncertain situations.^1 2^ Research shows that people with higher versus lower UT tend to feel less anxious, think more clearly and make decisions less influenced by uncertainty.^13^,^5^ While some see UT as a context-dependent ‘state’, we believe it’s likely that while UT fluctuates, an underlying ‘trait’-like stability shapes how individuals handle uncertainty.^6^

Across various specialities, higher UT is robustly associated with better psychological and work-life related well-being among clinicians (eg, reduced stress; increased resilience).^2^ Supporting doctors to manage uncertainty may thus help reduce endemic burnout among healthcare staff^7 8^—particularly in specialties with ‘high rates of undifferentiated illness and uncertainty’ as a large recent survey emphasised.^9^ While mixed, a plurality of evidence also shows that clinicians with higher UT make decisions that lead to lower resource use (eg, admit fewer patients; order fewer tests).^2^ This, combined with the possibility that higher burnout in low UT doctors could result in poor staff retention and/or more safety incidents, has led some to hypothesise that supporting staff management uncertainty could improve efficiency.^6 7^

Nevertheless, UT remains underexplored in the unique emergency medicine context. Most recent work has explored how emergency doctors navigate uncertainty rather than examining associations between UT and factors like clinical decision-making.^610^,^12^ Past studies looking at UT associations^13^,^17^ have had limitations. First, they were USA-based—poorly reflecting emergency department (ED) dynamics of resource-constrained universal healthcare systems.^18^ Second, they focused on specific tests/treatments (eg, CT scans) and/or patient groups—limiting generalisability. Third, they used the Physicians’ Reaction to Uncertainty Scale (PRUS) to assess UT which is not emergency medicine specific and primarily assesses emotional reactions to uncertainty (eg, anxiety) missing others (eg, behavioural) and undermining validity.^1^ Lastly, they did not assess UT associations with indicators of safety (eg, postdischarge mortality) despite the possibility that higher UT may cause patient harm through risky decision-making (eg, inappropriate discharges).^19^

We developed a more comprehensive, modified PRUS for the emergency medicine context and conducted an initial evaluation to assess associations between UT, doctor factors (eg, experience; burnout), resource use (eg, episode costs) and patient outcomes (eg, readmission). Based on literature from other specialties, we predicted that higher UT would be associated with lower resource use and better well-being indicators (eg, lower burnout). The association between UT and patient outcomes was considered either way; higher tolerance could lead to more risky decisions or alternatively, higher tolerance may protect doctors from cognitive ‘overload’—providing better judgement of costs/benefits of interventions which could have a beneficial impact on resource use and patient flow.^20^

## Method

### Study design

We developed an UT measure and tested it among emergency doctors, assessing its association with their demographics (eg, years of experience, gender), a chart review of their recent patient management decisions and outcomes (eg, episode costs, reattendance rates) and personality/work life factors (eg, self-perceived burnout, confidence, resilience). Online supplemental file 1 provides a study design diagram.

### Measures

#### UT measure

To measure doctors’ UT, the PRUS^21 22^ was adapted to better suit the emergency medicine context. Hillen *et al*^1^ conducted a comprehensive analysis of UT in healthcare identifying 22 existing self-report measures including the most prominent: PRUS. They categorised items across these measures according to a taxonomy of uncertainty sources (eg, complexity, unpredictability, disorder) and three types of responses: cognitive (eg, denial), emotional (eg, stress) and behavioural (eg, avoidance).

The research team held discussions over a series of meetings to create a comprehensive and context-specific measure. Starting with the PRUS, we incorporated relevant items from other validated measures that addressed the sources and responses to uncertainty described in Hillen’s model that were not fully covered by the original PRUS. Items were also modified/excluded if they were not relevant to emergency medicine.

The measure was piloted with seven emergency doctors whose feedback led to further revisions enhancing clarity and ensuring the content was appropriate to the National Health Service. The measure asks respondents to rate agreement from strongly disagree (1) to strongly agree (5) on 34 items, including: ‘I usually feel anxious when I am not sure of a diagnosis’ and *‘*The hardest thing to say to patients or their families is: I don’t know’. A score per doctor was derived by taking the mean (score range=1–5). It takes 7–9 min to complete. The measure can be seen in online supplemental file 2.

#### Personality and work-life measures

We captured other factors including resilience (6 items), risk aversion (1 item), burnout (1 item), confidence (5 items), work life well-being (10 items) and experience of an adverse event at work (1 item). For further details and specific items see online supplemental file 3.

#### Demographics

Doctors self-reported basic demographics including age, gender, hours worked a week, years of experience and seniority.

#### Patient selection criteria

The research team conducted an internal survey of 10 emergency doctors (from all sites except Leeds)—including members of the author team—ranking patient problems by greatest levels of uncertainty for the physician. These were used to guide the selection of patient charts to be reviewed for the association of a physician’s UT and patient management.

#### Doctor questionnaire

The final questionnaire was constructed through discussion and debate among the research team with some additional piloting work conducted with seven emergency doctors. In response to extensive pilot feedback, we kept the battery of measures brief using short and/or novel scales to reduce participant burden (eg, 1-item risk aversion scale). This pragmatic decision prioritised minimising slow recruitment, attrition and missing data over the potential loss of validity or reliability from using such measures.

The resulting questionnaire (online supplemental file 2) consisted of the UT measure, items about basic demographics (eg, age, grade), and the aforementioned personality and work-life measures. In total, it was 65 items and took 10–15 min to complete.

#### Participants

Doctors from five EDs in Yorkshire, UK completed all measures using an online questionnaire. Doctors at specialty trainee (ST) (year) 3 or equivalent and above were recruited only as they operate more independently than more junior doctors and thus make more independent and potentially impactful patient management decisions. Doctors were approached by collaborating colleagues and completed consent online (before the questionnaire). Recruitment commenced 18 May 2021 to 16 February 2022.

##### Patient episode outcomes

To correlate doctor responses on the questionnaire with patient-level variables, we manually extracted data from the 10 most recent patient encounters with each doctor from health records using a standardised form (online supplemental file 4). Eligible episodes met three criteria:

1. The episode occurred 30 days (minimum) before doctors completed the questionnaire to avoid any influence of the study on their decisions.
2. The patient’s primary problem was abdominal pain, back pain, chest pain, headache or vomiting. These were the top five problems ranked as leading to uncertainty by the internal survey.
3. The patient was 18+ years old.

In addition to patient-level demographic and clinical variables, we extracted resource use and patient outcomes to assess their correlation with UT. Online supplemental file 5 provides details on all data items and our cost estimation procedures.

##### Resource use indicators

- Ordering of any test and/or treatment per patient episode.
- 24-hour or shorter stay length among admitted patients (‘unnecessary admissions’).
- Estimated patient episode costs.

Patient episode costs were estimated using resource use and patient outcome indicators. The aim was not to estimate costs exactly but rather generate measures to assess variance in costs between doctors.

##### Patient outcomes

- 30-day attendance after initial attendance (‘reattendance’).
- 30-day admission to hospital on reattendance.

Though we captured mortality, it was not assessed as an outcome given just six deaths).

### Participants

#### Sample size

Multilevel regression power primarily depends on the sample size at the higher level (level 2) which in our study refers to the number of doctors versus patients (level 1). This was a challenge because we aimed to assess doctor-level variables as predictors of patient-level outcomes and doctor recruitment was much more resource-demanding than episode data extraction. According to simulation estimates derived by inputting a range of plausible values into a web app,^23^ a sample of 500 patients across 50 doctors (achievable within our time constraints) provided sufficient power (70–95%) to detect small to medium effect sizes (Cohen’s d of 0.30 or 0.50) when comparing median split UT differences on binary outcomes.

### Analysis

UT score distributions were assessed for variability and skewness. Cronbach’s α/McDonald’s ω assessed internal consistency and Pearson’s correlations, t-tests and analysis of variances (with Bonferroni-adjusted t-tests) were used to examine associations between UT scores and doctor-related factors.

Patient-level outcomes (ie, involving matched patient episode data) were assessed using multilevel logistics (eg, reattendance) or linear (costs) regression (random intercepts for doctors). Outcomes were regressed on doctor-level UT and adjusted for patient/doctor-level variables:

Model 1. Doctor UT + patient site

Model 2. Above + patient American Society of Anaesthesiologists (ASA) score.

Model 3. Above + patient age + patient gender

Model 4. Above + doctor experience (years) + doctor gender

Between-model fits were compared via Bayesian/Akaike Information Criteria. The within-model fit was assessed using *R^2^* or pseudo-*R^2^* metrics. Intraclass correlation coefficients (ICC) quantified between-doctor outcome variance.

It was hypothesised that higher UT would be associated with lower resource use, thus one-tailed tests were used. Two-tailed tests were planned for readmission, reattendance and mortality as it was hypothesised that UT could be associated in either direction. 95% CIs are provided for all estimates.

Missing data were minimal for most variables. Triage scores (acuity) had high missingness (eg, National Early Warning Scores (NEWS)) so ASA physical status scores were used for covariate adjustment instead. Sensitivity analyses assessed multiply-imputed acuity scores using all variables included in adjusted models as covariates. Other analyses assessed the removal of multivariate outliers and associations with specific versus composite indicators (eg, ECGs only vs all tests).

### Patient and public involvement

The research team held regular meetings with the Sheffield Emergency Care Forum and Yorkshire & Humber Patient Safety Translational Research Centres (PSTRC) patient panel. These groups were updated on the study progress and were provided feedback on the direction, management and findings throughout.

## Results

### Recruitment and sample characteristics

41 doctors participated and matched patient episodes were obtained for 39 of these doctors (78% of the target 50) (table 1). The mean doctor age was 35.3 and a mean of 8.3 years working in EDs; 63.4% were women. Among all respondents, 12.5% reported having ‘no’ burnout.

**Table 1:** Doctor and patient episode characteristics

Full doctor sample (n=41)			
Variable	Level	Value	n (%) miss.
Adverse event experienced? (n, %)	Yes	19 (47.5)	1 (2.4)
	No	21 (52.5)	
Age (Μ, SD)	–	35.3 (5.7)	0
Burnout (n, %)	None	5 (12.5)	1 (2.4)
	Some	26 (65)	
	Moderate	7 (17.5)	
	High	2 (5)	
Confidence (Μ, SD)	–	3.3 (0.4)	2 (4.8)
ED experience, **years** (Μ, SD)	–	8.3 (6.4)	0
Gender identity (n, %)	Male	15 (36.6)	0
	Female	26 (63.4)	
Grade* (n, %)	Specialty trainee year 3 (ST3)	7 (17.1)	1 (2.4)
	ST4	10 (24.4)	
	ST5	4 (9.8)	
	ST6	7 (17.1)	
	Consultant (highest level)	7 (17.1)	
	Other	6 (14.6)	
Resilience (Μ, SD)	–	3.3 (0.8)	1 (2.4)
Risk aversion (Μ, SD)	–	51.5 (21)	1 (2.4)
Site (n, %)	Airedale	6 (14.6)	–
	Barnsley	8 (19.5)	
	Bradford	15 (36.6)	
	Leeds	4 (9.8)	
	Sheffield	8 (19.5)	
Uncertainty tolerance (Μ, SD)	–	3.6 (0.4)	0
Weekly A&E hours (Μ, SD)	–	31.8 (10)	0
Work-related well-being (Μ, SD)	–	3.5 (0.4)	1 (2.4)

Patient episode sample (n=384)			
Variable	Level	Value	n (%) miss.
Admission (initial) (n, %)	Yes	100 (26)	0
	No	284 (74)	
Admission (return, 30 days) (n, %)			0
All	Yes	29 (7.6)	
	No	355 (92.4)	
Related to initial attendance?	Yes	26 (89.7)	
	No	3 (10.3)	
Related: number per patient (Μ, SD)	–	1.3 (0.6)	
Age (Μ, SD)	–	46.6 (18.8)	0
ASA score: continuous (Μ, SD)	–	1.9 (0.9)	2 (0.5)
Case complexity (Μ, SD)	–	2.9 (0.8)	3 (0.8)
Problem type (primary) (n, %)	Ab pain	99 (25.8)	0
	Back pain	33 (11.7)	
	Chest pain	162 (42.2)	
	Headache	36 (9.4)	
	Vomiting	7 (1.8)	
	Combo of>1	47 (12.2)	
Cost index v1: tests/treat (Μ, SD)	–	£32.3 (48.8)	0
Cost index v2: +admissions (Μ, SD)	–	£634.9 (1250.5)	0
Cost index v3: +re att./return admit. (Μ, SD)	–	£854.4 (1583.9)	0
Death (30-day) (n, %)			
All	Yes	6 (1.6)	
	No	378 (98.4)	
Related to initial attendance?	Yes	3 (50)	
	No	3 (50)	
Diagnosis status discharge (n, %)	Confirmed	102 (29.6)	39 (10.2)
	Suspected	243 (70.4)	
Discharge location (n, %)	Home	357 (92.9)	0
	Other	27 (7.1)	
Gender identity (n, %)	Male	174 (45.3)	0
	Female	210 (54.7)	
Hospital stay length in days (Μ, SD)	–	1.2 (4.3)	0
If admitted (Μ, SD)	–	4.5 (7.4)	0
Mode of arrival (n, %)	Ambulance	104 (30.4)	42 (11)
	Other	238 (69.6)	
NEWS (Μ, SD)	–	0.8 (0.9)	265 (69)
NEWS2 (Μ, SD)	–	1.1 (1.4)	96 (25)
Ordered tests (by doctors) (n, %)	Blood tests	84 (21.9)	0
	CT	29 (7.6)	
	ECG	20 (5.2)	
	MRI	1 (0.3)	
	USS	6 (1.6)	
	X-ray	113 (29.4)	
	Other	36 (9.4)	
Possible decision deferral† (n, %)	Yes	100 (26)	0
	No	284 (74)	
Reattendance (return, 30-day) (n, %)			0
All	Yes	61 (15.9)	
	No	323 (84.1)	
Related to initial attendance?	Yes	54 (88.5)	
	No	7 (11.5)	
Related: number per patient (Μ, SD)	–	1.7 (1.9)	
Site (n, %)	Airedale	38 (9.9)	–
	Barnsley	86 (22.4)	
	Bradford	139 (36.2)	
	Leeds	41 (10.7)	
	Sheffield	80 (20.8)	
Waiting time hours (initial) (Μ, SD)	–	2.6 (2.1)	2 (0.5)

* Note: In the UK: ST3–ST6: Specialty trainees in years 3–6 after foundational training equivalent to residents or registrars. Consultant: Fully qualified senior specialists similar to attending physicians. Other (eg, specialty doctors, trust grade doctors): For example, doctors not in formal training posts but with comparable experience.

† Defined as any recorded evidence that the treating (ie, patient-matched) doctor conferred with a colleague about patient management decisions (admission, treatments and/or tests). Note, the full (41) and episode-matched (39) doctor samples showed minimal differences.

Ab painabdomen painA&Eaccident and emergencyASAAmerican Society of AnaesthesiologistsEDemergency department% miss% missing dataNEWSNational Early Warning ScoresRe-att./rtn admit30 day reattendance or admission on returnΜmean

Data for 384 patients were extracted and matched to the doctors (median=10, range=1–22 patients each). 54.7% of matched patients were women and their mean age was 46.6. The most prominent problems were chest (42.2%) and abdominal (25.8%) pain. Across episodes, doctors admitted 26% of patients. Tests ordered most were X-rays (29.4% episodes) and bloods (21.9%), the least MRI (0.3%) scans. 16% of patients reattended within 30 days of initial attendance, 7.6% were readmitted.

### UT and doctor factors

Doctors’ mean UT score was 3.6/5 (range=2.6–4.8). The measure showed excellent internal reliability (α=0.92/ω=0.92^24^) indicating items consistently assessed one construct. Scores had low skewness (−0.01) and kurtosis (1.01) suggesting scores were evenly distributed around the mean with no extreme outliers or a sharp peak.

**Figure 1:**
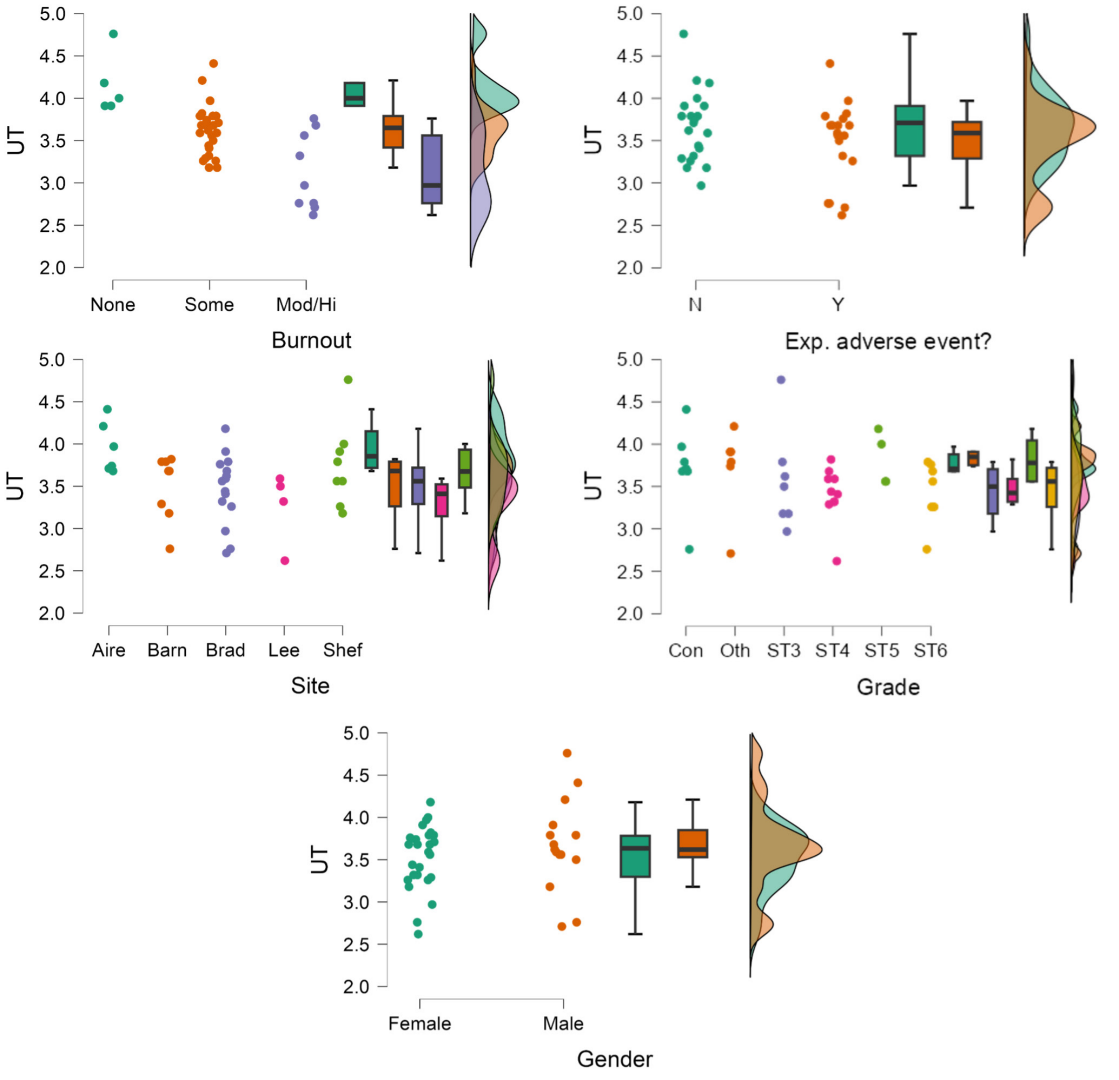
Jitter plots, box plots and density plots for associations between uncertainty tolerance (UT) (higher=more tolerant) and all categorical variables: burnout, experience of an adverse event, site, grade and gender.

**Figure 2:**
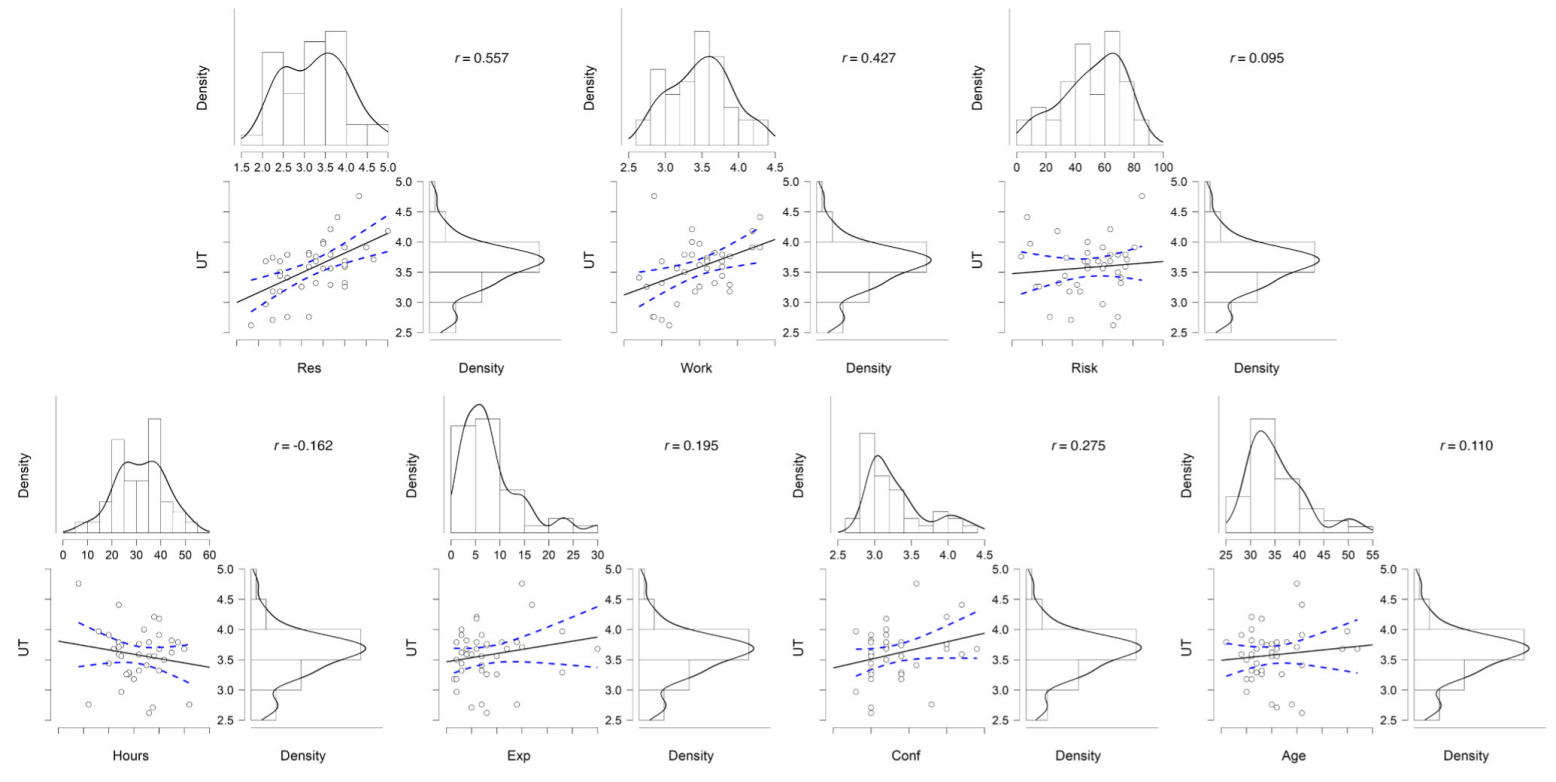
Histograms and density plots for all continuous variables plus scatterplots (with Pearson’s r estimates) between uncertainty tolerance (UT) (higher=more tolerant) and continuous variables: res (resilience), work (work life well-being), risk (risk aversion), hours (hours worked weekly), exp (years of experience), conf (confidence) and age. Could this be n (%)—clearer to readers fair point but it cannot be as the column sometimes references means and SD (eg, for age).

UT associations with categorical variables (eg, burnout) are shown in figure 1 and with continuous variables (eg, age in figure 2). Individuals with no burnout reported significantly higher tolerance compared with those with some (mean diff.=0.53; Cohen’s d=1.53; 95% CI=0.23 to 2.83) and moderate/high (collapsed categories) burnout (mean diff.=1.03; Cohen’s d=2.98; 95% CI=1.33 to 4.62). In turn, those with some reported significantly higher tolerance than those with moderate/high burnout (mean diff.=0.50; Cohen’s d=1.45; 95% CI=0.39 to 2.51).

Higher resilience was also strongly associated with higher UT (Pearson’s r=0.56; 95% CI=0.30 to 0.74) as was higher work-life well-being (0.43; 0.13 to 0.65). Though higher confidence correlated with higher UT (0.27; −0.04 to 0.54), this was non-significant (p=0.09). There were significant UT differences between sites.

Experience of adverse events, hours worked, gender and seniority was not notably associated with UT.

### UT and patient-level outcomes

Table 2 provides patient-level outcomes results. Point estimates indicated that as UT increased, episode costs decreased (eg, in Model IV looking at total costs shows there was a 7% decrease in costs per one UT scale point increase though we could not statistically rule out estimated increases in costs (0.07, 95% CI: −0.32 to 0.18). ICC was generally low (meaning there was low between-doctor variation in this outcome) and models explained small-to-moderate variance.

**Table 2:** Uncertainty tolerance (UT) associations with patient-level outcomes

Model	UT estimate	−95% CI	+95% CI	P value*	R^2^_M_ (R^2^_C_)	ICC	AIC | BIC
**Logistic models (ORs)**							
**Tests: any ordered?**							
Model I	0.75	0.40	1.41	0.18^1t^	14% (18%)	5%	498 | 525
Model II	0.72	0.38	1.36	0.16^1t^	14% (18%)	5%	496 | 528
Model III	0.72	0.38	1.35	0.16^1t^	17% (21%)	5%	**491 | 530**
Model IV	0.74	0.40	1.36	0.17^1t^	18% (21%)	4%	493 | 541
**Treatments: any ordered?**							
Model I	0.88	0.52	1.49	0.32^1t^	6% (7%)	1%	515 | 543
Model II	0.87	0.52	1.45	0.30^1t^	9%	<1%	**508 | 539**
Model III	0.90	0.54	1.52	0.35^1t^	11%	<1%	506 | 545
Model IV	0.93	0.55	1.59	0.40^1t^	14%	<1%	500 | 548
**Treatments or tests: any ordered?**							
Model I	0.65	0.29	1.47	0.15^1t^	11% (22%)	13%	**451 | 478**
Model II	0.64	0.29	1.43	0.14^1t^	11% (22%)	12%	451 | 483
Model III	0.66	0.30	1.46	0.15^1t^	13% (23%)	11%	448 | 488
Model IV	0.71	0.35	1.45	0.16^1t^	18% (23%)	7%	446 | 494
**Return attendance 30 days after presentation**							
Model I	0.95	0.47	1.92	0.89^2t^	4%	<1%	320 | 348
Model II	0.97	0.47	2.00	0.94^2t^	9%	<1%	**312 | 344**
Model III	0.95	0.46	1.95	0.88^2t^	13%	<1%	311 | 351
Model IV	0.97	0.46	2.04	0.94^2t^	13%	<1%	314 | 362
**Admission 30 days postpresentation**							
Model I	0.77	0.28	2.11	0.61^2t^	18%	<1%	191 | 219
Model II	0.79	0.28	2.26	0.66^2t^	24%	<1%	**185 | 217**
Model III	0.80	0.28	2.30	0.67^2t^	24%	<1%	189 | 229
Model IV	0.82	0.28	2.35	0.71^2t^	24%	<1%	192 | 240
**24-hour or shorter length of stay**							
Model I	0.91	0.35	2.34	0.43^1t^	98%	<1%	**196 | 224**
Model II	0.91	0.35	2.34	0.42^1t^	98%	<1%	198 | 230
Model III	0.97	0.36	2.59	0.48^1t^	98%	<1%	198 | 238
Model IV	0.97	0.36	2.59	0.73^1t^	98%	<1%	198 | 245
**Linear models (unstandardised *β*)†**							
**Test and treatment costs**							
Model I	−0.01	−0.06	0.05	0.37^1t^	8% (12%)	4%	**−162 | −130**
Model II	−0.01	−0.06	0.04	0.36^1t^	9% (13%)	3%	−156 | −120
Model III	−0.01	−0.06	0.04	0.31^1t^	13% (16%)	3%	−150 | −106
Model IV	−0.01	−0.06	0.04	0.32^1t^	13% (16%)	4%	−130 | −79
**+ admission costs**							
Model I	0.02	−0.24	0.28	0.56^1t^	16% (17%)	1%	1122 | 1153
Model II	−0.002	−0.24	0.24	0.49^1t^	23%	<1%	1088 | 1123
Model III	−0.005	−0.24	0.23	0.48^1t^	27%	<1%	**1082 | 1126**
Model IV	−0.002	−0.23	0.23	0.48^1t^	28%	<1%	1094 | 1145
**+ reattendance and return admission costs**							
Model I	−0.05	−0.34	0.24	0.37^1t^	17% (19%)	2%	1182 | 1213
Model II	−0.07	−0.33	0.19	0.29^1t^	25% (26%)	<1%	**1141 | 1177**
Model III	−0.08	−0.33	0.17	0.28^1t^	27%	<1%	1146 | 1190
Model IV	−0.07	−0.32	0.18	0.28^1t^	28%	<1%	1156 | 1208

R2_M_=variance explained by fixed effects.

R2_C_=variance explained by fixed+random effects (where random effects variance minimal R2_C_=R2_M_).

Bold AIC/BIC 2=model with the lowest AIC+BIC.

* 1t/2t=one/two-tailed tests.

† Note the natural logarithm of all outcomes was used to mitigate skew after a constant of 182 was added to cost outcomes.

AICAkaike Information CriteriaBICBayesian Information CriteriaICCintraclass correlation coefficients

Tolerance was not significantly associated with the composite outcomes of rates of ordering ‘any test’, ‘any treatment’ or ‘any or test or treatment’. Models here explained small amounts of variance and ICC was low. UT was also not significantly associated with the proportion of patients with≤24-hour hospital stay after admission (extremely high *R^2^* values indicated overfitting, meaning estimates should be interpreted cautiously). When the composite primary outcome of ‘any tests ordered’ was broken down into singular test outcomes (eg, CT scan: yes vs no), higher UT was significantly associated with less ECG ordering*—*but no other tests (see online supplemental file 6). Analyses showed a small decrease in reattendance and readmission rates with increased UT but estimates were again not significant. Models had low to moderate *R^2^* values meaning other important variables were unaccounted for.

Coefficients were practically equivalent when NEWS2 values were imputed and used in place of ASA scores. Removing multivariate outliers did not produce notable changes.

## Discussion

Our UT measure demonstrated a good range and balance of scores, high internal consistency and significant correlations with self-reported doctor characteristics such as higher psychological resilience and lower burnout. There was no strong evidence of an association between tolerance and resource use; results indicated no significant associations and while coefficients mostly suggested an increase in costs with increased tolerance, CIs showed results consistent with the opposite. This pattern was also seen in patient outcomes.

While our UT measure addresses limitations with the PRUS including making it relevant to the UK emergency medicine context, it remains to be seen how the measure performs in further psychometric testing—for example, test–retesting—in larger samples. The measure appeared to be internally consistent and alpha/omega values were so high that some items may have been redundant and less burdensome measures could be derived. Future work within our team aims to conduct a more thorough validity assessment of our measure. We welcome future studies to adapt and assess the measure for UT assessment in other medical specialties and see whether scale-assessed tolerance is stable, modifiable and predictive of change in future behaviour.

The lack of significant associations between UT and resource use mirror findings from previous emergency medicine research,^13^,^17^ though not all specialties.^3^ One reason could be that patient pathways in emergency medicine are often protocolised, making significant practice variance difficult to capture. Homogenising the sample to ST3+ (ie, resident+level) doctors may have further reduced variation. Nonetheless, UT was linked with increased staff well-being suggesting it could be associated with resource use outcomes not captured here such as fewer staff sick days (eg, from burnout) and (eg, stress-induced) clinical errors.

Additional complexities in interpreting ED-based studies of UT arise from overlapping staff dynamics. In secondary analyses, we found that UT is significantly associated with lower ECG ordering rates but such relationships may be challenging to assess where several staff groups such as both nurses and doctors are empowered to order the same tests. Future studies could disentangle these subtleties, perhaps through path models and qualitative work.

For return admission outcomes, though small, regression coefficients indicated lower return rates with higher UT although it with CIs consistent with higher return rates. Thus, we could at least provide some evidence against positive associations of a medium to large size (ie, ORs above the upper estimate of our intervals which rarely exceeded 2). Future research needs to replicate our work with larger sample sizes to assess whether there are other outcomes not captured here that may be more sensitive or further contextual or other doctor factors which may moderate or mediate associations. For example, it could be that because the patients evaluated in this study all had high ‘uncertainty’ problems there was a ceiling effect; with more variation in problems, perhaps there would be more variation in decision-making processes sensitive to UT.

Consistent with previous studies,^1 9^ the differences in UT across sites suggest that it may be influenced by contextual factors. Future research could identify and investigate departments where doctors exhibit relatively higher average UT, providing insights into contributing factors that could be applied in other organisations^25^ to boost work-related well-being.

While we are unaware of trials of interventions to improve UT for doctors, recent research has identified uncertainty management strategies emergency doctors use already, providing a basis for intervention development.^10^,^1226^ Patel demonstrated how visual thinking strategies and interactions with horses can prepare medical students to manage uncertainty by exposing them to a variety of clinical and non-clinical scenarios.^27^ Ilgen *et al* also highlighted that UT may manifest not only as a personality trait but also as a situational response that varies with clinical context.^6^ These insights suggest a need for interventions that are both adaptive and context-specific using real-world scenarios to effectively cultivate tolerance among healthcare staff.

### Limitations

Records data were taken retrospectively, so some doctors may have deferred decisions to colleagues without noting it, undermining the assumption that costs resulted from their decisions. Similarly, UT scores may not reflect past scores if tolerance-modifying events occurred between episodes and doctor participation.

Because the coefficients in our analysis cannot be interpreted causally, future studies should consider modifying UT through randomised trials to assess causal effects. We did not meet our sample size targets due to overestimating achievable recruitment rates; as a result, our CIs were wider and less precise, making it difficult to detect smaller effects suggested by our point estimates. Convenience sampling may have biased our doctor sample towards those who are more confident and relying on self-reported data could have introduced social desirability bias.^28^ Additionally, the self-selection of sites may have led to the inclusion of locations with specific characteristics such as particular organisational cultures.

While no associations between UT and negative patient outcomes were found, the proxy measures chosen may have been too insensitive. Future research should capture broader outcomes such as 30-day patient general practitioner visits and provide more granular analyses of how UT relates to outcomes in specific contexts. For example, research could identify patient groups that are more often inappropriately dismissed or overlooked by doctors with higher UT and assess how this more targeted issue could be mitigated.

Studies have suggested UT can only be investigated as ‘state’^11 12^ but this is disputed^1 10^ not least because UT may be stable over time.^29^ Further, some argue neither extreme tolerance nor intolerance may be inherently beneficial or detrimental as each could provide advantages and expose clinicians to risks in practice, undermining our assumptions that higher scores were ‘better’.^30^

While costs were taken from tariffs, some potentially unrealistic assumptions were made, for example, assuming a medication packet was opened for each given dose and some costs were missed such as tests on reattendance.

Some measures used only one question for example, risk aversion and burnout thus not very sensitive to the complexity of these concepts.

## Conclusion

Higher doctor UT on a novel measure was associated with higher self-reported psychological and work-related well-being but was not significantly associated with patient outcomes or most measures of resource use. Interventions to support emergency doctors to better tolerate uncertainty might have positive implications for them but our study is unable to answer the question as to whether there are positive or negative side effects on patient outcomes. It is necessary for future work to replicate and build on our study to resolve such ambiguities.

## supplementary material

10.1136/emermed-2023-213256 online supplemental file 1

10.1136/emermed-2023-213256 online supplemental file 2

10.1136/emermed-2023-213256 online supplemental file 3

10.1136/emermed-2023-213256 online supplemental file 4

10.1136/emermed-2023-213256 online supplemental file 5

10.1136/emermed-2023-213256 online supplemental file 6

## Data Availability

Data are available upon reasonable request.
