# Supplementary material for: Is emergency doctors’ tolerance of clinical uncertainty on a novel measure associated with doctor well-being, healthcare resource use and patient outcomes?
Source: Emerg Med J. 2024 Nov 27;42(1):e213256. doi: 10.1136/emermed-2023-213256 (PMC11874457; doi:10.1136/emermed-2023-213256)
Supplement: online supplemental file 2 [file emermed-42-1-s002.pdf]

Doctor questionnaire

Doctor ID: _______

Thank you for taking the time to complete this questionnaire.

It is **65 items** long, is in **3 parts** and takes around **10-15 minutes** to complete. As outlined in the information sheet:

Part 1 asks you to provide some demographic information.

Part 2 asks you to answer questions related to your tolerance of uncertainty.

Part 3 asks you to answer questions related to your personality and work life.

Please answer as accurately as possible without skipping questions.

Part I

Demographics

7 items, ~1 minute

1	How old are you?				
2	How do you identify?				
	Female	Male		Other	
3	How many clinical A&E hours do you work a week on average?				
4	How long have you worked in A&E?				
5	What year did you qualify as a doctor?				
6	What is your grade (or equivalent?)				
ST3	ST4	ST5	ST6	Other	
7	Would you be willing to take part in a follow-up interview about uncertainty?				

Focus group	Interview	Either	None

Part II
Uncertainty tolerance

34 items ~-7-9 minutes

	All the below statements relate to your work life.<br><br>Please tick the response that best represents your level of agreement with each statement.	Strongly disagree	Disagree	Neither agree or disagree	Agree	Strongly agree
1	The uncertainty of patient care often troubles me					
2	Not being sure of what is best for a patient is one of the most stressful parts of being a doctor					
3	I am tolerant of the uncertainties present in patient care					

	All the below statements relate to your work life.<br><br>Please tick the response that best represents your level of agreement with each statement.	Strongly disagree	Disagree	Neither agree or disagree	Agree	Strongly agree
4	I find the uncertainty involved in patient care disconcerting					
5	I usually feel anxious when I am not sure of a diagnosis					
6	When I am uncertain of a diagnosis, I imagine all sorts of bad scenarios –					

	All the below statements relate to your work life.<br><br>Please tick the response that best represents your level of agreement with each statement.	Strongly disagree	Disagree	Neither agree or disagree	Agree	Strongly agree
	patients dies, patient sues etc.					
7	I am frustrated when I do not know a patient's diagnosis					
8	I fear being held accountable for the limits of my knowledge					
9	Uncertainty in patient care makes me uneasy					

	All the below statements relate to your work life.<br><br>Please tick the response that best represents your level of agreement with each statement.	Strongly disagree	Disagree	Neither agree or disagree	Agree	Strongly agree
10	I worry about malpractice when I do not know a patient's diagnosis					
11	The vastness of the information doctors are expected to know overwhelms me					
12	I frequently wish I had gone into a specialty that would minimise the					

	All the below statements relate to your work life.<br><br>Please tick the response that best represents your level of agreement with each statement.	Strongly disagree	Disagree	Neither agree or disagree	Agree	Strongly agree
	uncertainties of patient care					
13	I am quite comfortable with the uncertainty in patient care					
14	The hardest thing to say to patients or their families is ‘I don’t know’					
15	When doctors are uncertain of a diagnosis,					

All the below statements relate to your work life.<br><br>Please tick the response that best represents your level of agreement with each statement.		Strongly disagree	Disagree	Neither agree or disagree	Agree	Strongly agree
	they should share this information with their patients					
16	If I share my uncertainties with patients, I will increase the likelihood that I will be sued					

All the below statements relate to your work life.<br><br>Please tick the response that best represents your level of agreement with each statement.		Strongly disagree	Disagree	Neither agree or disagree	Agree	Strongly agree
17	I almost never tell other doctors about diagnoses I have missed					
18	If I shared all of my uncertainties with my patients they would lose confidence in me					
19	I am afraid other doctors would doubt my ability if					

	All the below statements relate to your work life.<br><br>Please tick the response that best represents your level of agreement with each statement.	Strongly disagree	Disagree	Neither agree or disagree	Agree	Strongly agree
	they knew about my patient care mistakes					
20	If I do not make a diagnosis, I worry that the referring doctor will stop sending patients to me					
21	I never tell other doctors about patient care mistakes I have made					

All the below statements relate to your work life.<br><br>Please tick the response that best represents your level of agreement with each statement.		Strongly disagree	Disagree	Neither agree or disagree	Agree	Strongly agree
22	I always share my uncertainty with patients					
23	There is a right way and a wrong way to almost anything in medicine					
24	I will never be as good a doctor as I could be, because of the unpredictability in patient care					

	All the below statements relate to your work life.<br><br>Please tick the response that best represents your level of agreement with each statement.	Strongly disagree	Disagree	Neither agree or disagree	Agree	Strongly agree
25	I often get curious about what happened to patients I cared for					
26	I would not have confidence in a medical test or treatment if there were conflicting opinions about it					
27	When I find myself in an uncertain clinical					

	All the below statements relate to your work life.<br><br>Please tick the response that best represents your level of agreement with each statement.	Strongly disagree	Disagree	Neither agree or disagree	Agree	Strongly agree
	situation I tend to have doubts about what I'm doing					
28	It's more enjoyable to treat a patient with unclear symptoms					
29	Novel cases I have not seen before are exciting					
30	When I have to help a patient with vague,					

		Strongly disagree	Disagree	Neither agree or disagree	Agree	Strongly agree
All the below statements relate to your work life.<br><br>Please tick the response that best represents your level of agreement with each statement.						
	ambiguous complaints, uncertainty paralyses me					
31	When things are chaotic at work I will try to regain control					
32	I tend to give up easily when I don't clearly understand a situation					

	All the below statements relate to your work life.<br><br>Please tick the response that best represents your level of agreement with each statement.	Strongly disagree	Disagree	Neither agree or disagree	Agree	Strongly agree
33	When there is a lot of patient variety at work, I function well					
34	It gets me down working in an environment which is constantly changing					

Part III

Personality & work life

24 items, ~2-3 minutes

1	**In your career, have you been involved in a patient safety incident (or near miss) which has had long lasting, negative emotional impact on you?**

**Yes**	**No**

3	**Please tick next the <u>one</u> statement you identify with most…**

**I enjoy my work. I have no symptoms of burnout.**	
**Occasionally I am under stress, and I don't always have as much energy as I once did, but I don't feel burned out.**	
**I am definitely burning out and have one or more symptoms of burnout, such as physical and emotional exhaustion.**	
**The symptoms of burnout that I'm experiencing won't go away. I think about frustration at work a lot.**	

I feel completely burned out and often wonder if I can go on. I am at the point where I may need some changes or may need to seek some sort of help.	

2	From 1 (risk avoider) to 10 (risk seeker) how risk averse do you consider yourself?								
1	2	3	4	5	6	7	8	9	10

These questions relate to your confidence as a doctor. Please tick one box to complete these statements.

4	I am generally…	Very poor		…at exercising clinical judgement to establish diagnoses
		Poor		
		Good		
		Very good		
		Excellent		
5	I am generally…	Very poor		…at exercising clinical judgement to establish therapies/treatments
		Poor		
		Good		
		Very good		
		Excellent		
6	I am generally…	Very poor		…at managing patients in an effective manner
		Poor		
		Good		
		Very good		
		Excellent		
7	I am generally…	Very poor		…at managing patients in an efficient manner
		Poor		
		Good		
		Very good		
		Excellent		
8	I am generally…	Very poor		…at managing patients in an ethical manner
		Poor		
		Good		
		Very good		
		Excellent		

	All the below statements relate to your resilience as a person. This could be at work or in your personal life.<br><br>Please tick the response that best represents your level of agreement with each statement.	Strongly disagree	Disagree	Neither agree or disagree	Agree	Strongly agree
9	I tend to bounce back quickly after hard times					
10	I have a hard time making it through stressful events					
11	It does not take me long to recover from a stressful event					
12	It is hard for me to snap back when something bad happens					

13	I usually come through difficult times with little trouble					
14	I tend to take a long time to get over setbacks in life					

All the below statements relate to your work environment.<br><br>Please tick the response that best represents your level of agreement with each statement.		Not at all	To a small extent	To some extent	To a large extent	To a very large extent
15	Does your work in A&E give you chance to demonstrate your clinical skills and abilities?					
16	Does your work in A&E help your self-confidence?					
17	To what extent do you feel nervous when working in A&E?					
18	To what extent do you find you can use your knowledge and experience in your work in A&E?					
19	Is patient treatment in A&E complicated by conflicts among staff members?					

**20**	**Is it difficult to reconcile loyalty towards your team with loyalty towards your own profession?**					

All the below statements relate to your work environment.<br><br>Please tick the response that best represents your level of agreement with each statement.		Never	Rarely	Occasionally	Often	Very often
21	How often are worried about going to work?					
22	Do you get the support you need when you are faced with difficult treatment problems?					
23	Do you feel like you need to be in several places at the same time in A&E?					
24	How often do you think you have too many tasks imposed on you?					

Thank you again for taking the time to complete this questionnaire.
