# Supplementary material for: Is emergency doctors’ tolerance of clinical uncertainty on a novel measure associated with doctor well-being, healthcare resource use and patient outcomes?
Source: Emerg Med J. 2024 Nov 27;42(1):e213256. doi: 10.1136/emermed-2023-213256 (PMC11874457; doi:10.1136/emermed-2023-213256)
Supplement: online supplemental file 3 [file emermed-42-1-s003.pdf]

Table 1. Overview of study variables.

Level	Variable type	Variable	Measure/source	Purpose
Doctor	***Characteristics***	• Age<br>• Gender identity<br>• Clinical hours per week<br>• Grade<br>• Years worked in ED (experience)	All self-report	To assess sample characteristics, to control for confounders, and to assess the association between demographic variables and UT (e.g. to characterise potential intervention recipients)
		• Experience of a safety incident	Self-report. Adapted from [1] yes/no)	
		• Risk aversion*	1 item self-report adapted from [2] (range = 0-100, > = less averse)	
		• Burnout	1 item self-report from [3] (1-4, > = more burnout)	
		• Confidence	5 item self-report (novel measure) (1-5, mean, > = more confident)	
		• Psychological resilience	6 item self-report Brief Resilience Scale [4] (1-5, mean, > = more resilient)	
		• Work life wellbeing	10 item self-report (novel measure) (1-5, mean, > = better)	
	Uncertainty tolerance		34 item Likert self-report (based on Physicians' Reaction to Uncertainty scale [5, 6] (1-5, mean, > = higher UT)	Primary predictor variable
Patient	***Characteristics***	• Age<br>• Reported gender	Record extraction	To assess sample characteristics and to control for possible extraneous factors

Table 1. Overview of study variables.

Level	Variable type	Variable	Measure/source	Purpose
		• Health status	1. Extractor judged American Society of Anesthesiologists physical status classification system (ASA) scores (co-morbidity) (1 healthy to 4 'severe' comorbidities) based on notes,<br>2. Triage scores: National Early Warning Scores (NEWS, NEWS2) (0 healthy to 20 very unwell), Manc. Triage (5 non-urgent to 1 immediate)	
	Episode characteristics	• Mode of arrival<br>• Diagnosis<br>• Comorbidities	All record extraction	To provide a descriptive overview of the sample within and between sites
		• Complaints		To confirm met inclusion criteria, and to provide a descriptive overview of the sample within and between sites
		• Case complexity	Extractor judged with a 2 item Likert scale (1-5, mean, > = more complex)	To assess whether case complexity moderates association(s) of interest
	Resource use**	• Admission status<br>• Hospital stay length<br>• Ordered tests (total/type)<br>• Ordered treatments (total/type)	All record extraction	To assess whether lower UT doctors have patients with shorter stay lengths and order fewer tests/treatments. To estimate and model episode costs as an outcome
	Patient health	• 30-day re-attendance***<br>• 30-day admission upon return<br>• 30-day mortality	All record extraction	To assess whether doctor UT is associated with adverse event rates. To estimate and model episode costs as an outcome
	Episode costs		Record extraction	Episode costs were used as a primary outcome; costs were estimated based on *resource use* and *patient health* outcome data (see Supp. File 3).

Table 1. Overview of study variables.

Level	Variable type	Variable	Measure/source	Purpose

*The mean of items was used for multi-item doctor measures. **Data quality checks were included in the form e.g. contamination; whether patients' notes indicated that each doctor deferred their decision to admit them or consult when ordering treatment/tests. ***Return admittance/re-attendance data included patients re-presenting for conditions related to the initial complaint; reflecting returning patients who may have benefitted from admission in the initial presentation. Presentations for unrelated conditions were considered unlikely to be related to doctors' discharge decisions and were not counted.

[1] E. van Gerven, L. Bruyneel, M. Panella, M. Euwema, W. Sermeus and K. Vanhaecht, “Psychological impact and recovery after involvement in a patient safety incident: A repeated measures analysis,” *BMJ Open,* vol. 6, no. 8, pp. doi: 10.1136/bmjopen-2016-011403, 2016.

[2] T. Dohmen, A. Falk, D. Huffman, U. Sunde, J. Schupp and G. G. Wagner, “Individual risk attitudes: Measurement, determinants, and behavioral consequences,” *Journal of the European Economic Association,* vol. 9, no. 3, pp. 552-550, doi: 10.1111/j.1542-4774.2011.01015.x, 2011.

[3] V. Hansen and A. Girgis, “Can a single question effectively screen for burnout in Australian cancer care workers?,” *BMC Health Services Researc,* vol. 10, no. 1, pp. 1-4, doi: 10.1186/1472-6963-10-341, 2010.

[4] B. W. Smith, J. Dalen, K. Wiggins, E. Tooley, P. Christipher and J. Bernard, “The brief resilience scale: Assessing the ability to bounce back,” *International Journal of Behavioral Medicine,* vol. 15, no. 3, pp. 194-200, doi: 10.1080/10705500802222972, 2008.

[5] M. S. Gerrity, K. P. White, R. F. DeVellis and R. S. Dittus, “Physicians' reactions to uncertainty: refining the constructs and scales,” Motivation and Emotion, vol. 19, no. 3, pp. 175-191, 1995.

[6] M. S. Gerrity, R. F. DeVellis and J. A. Earp, “Physicians' reactions to uncertainty in patient care: A new measure and new insights,” Medical Care, pp. 724-736, 1990.
