# Supplementary material for: Is emergency doctors’ tolerance of clinical uncertainty on a novel measure associated with doctor well-being, healthcare resource use and patient outcomes?
Source: Emerg Med J. 2024 Nov 27;42(1):e213256. doi: 10.1136/emermed-2023-213256 (PMC11874457; doi:10.1136/emermed-2023-213256)
Supplement: online supplemental file 4 [file emermed-42-1-s004.pdf]

The TofU project: Can emergency doctors' tolerance of uncertainty impact on patient outcomes and resource utilisation?

Please extract data for **10 patients** per doctor. Please pick the most recent **eligible** patients (i.e. do not pick at random). To be included, a patient:

1. Must have been over 18 years old
2. The patient episode must have been over 30 days ago
3. Must have arrived with at least 1 of the following **primary** complaints: **abdominal pain**, **chest pain**, **headache**, **vomiting** and/or **back pain**.
4.

Please note that the 'treating doctor' refers to the doctor who did or did not make the decision to admit/discharge the patient.

Please try to complete every section (even if the patient died) as missing data will affect the statistical power of our analyses. If there are sections you cannot complete, please make a note of these, and reasons why, in the last section of this form.

We have included a completion guide/explanation for each item at the end of this form.

---

1. What is your extractor ID?

2. What are the doctor and patient's IDs?

**Doctor**		**Patient**	

3. What site was this patient seen at?

Airedale	Barnsley	Bradford	Sheffield	Leeds

4. Date of presentation

DD	MM	YY

5. Mode of arrival

999 road ambulance	Other

6. What was the patient's age?

7. What was the patient's gender?

Female	Male	Other

8. What triage scores did the patient receive? **(note all scores that were recorded)**

Manchester triage	NEWS	NEWS2	Other(s)
**If no score was documented, what equivalent score(s) would you give the patient based on any written information?**			

9. What pre-existing co-morbidities are listed for this patient?

10. Based on comorbidities, in your view this patient was… **(tick one option)**

Grade	Definition	Tick
**ASA I**	**A normal healthy patient**. Example: Fit, non-obese (BMI under 30), a non-smoking patient with good exercise tolerance.	
**ASA II**	**A patient with a mild systemic disease**. Example: Patient with no functional limitations and a well-controlled disease (e.g., treated hypertension, obesity with BMI under 35, frequent social drinker or is a cigarette smoker).	
**ASA III**	**A patient with a severe systemic disease that is not life-threatening**. Example: Patient with some functional limitation as a result of disease (e.g., poorly treated hypertension or diabetes, morbid obesity, chronic renal failure, a bronchospastic disease with intermittent exacerbation, stable angina, implanted pacemaker).	
**ASA IV**	**A patient with a severe systemic disease that is a constant threat to life**. Example: Patient with functional limitation from severe, life-threatening disease (e.g., unstable angina, poorly controlled COPD, symptomatic CHF, recent (less than three months ago) myocardial infarction or stroke.	

**Unsure**	Based on the case notes, it is not possible to estimate.	

11. Arrival and assessment times

Arrival time	Time saw treating doctor

12. Was the patient admitted?

Yes	No
If so - at what time? **(24-hr clock)**	
If so - to which specialty?	

13. Was there any written evidence that the treating doctor discussed the decision to admit/discharge with a senior ED or a specialist clinician?

Yes	No
If so - what was written? And where?	

14. After assessment and/or treatment where was the patient discharged to?

Home	Care home	Other hospital	Died	Other

15. Date of discharge from hospital

**Same as presentation**	***If after a stay…***		
	**DD**	**MM**	**YY**

16. Discharge time

**Planned discharge time** (24-hr clock)	**Time left the department** (24-hr clock)

17. What tests were made and who ordered these? (respond for each with the time)

Test	Treating doctor		Another clinician		Routinely ordered at triage		Unsure
**X-Ray**							
	Time?		Time?		Time?		
**CT**							
	Time?		Time?		Time?		
**USS**							
	Time?		Time?		Time?		
**MRI**							
	Time?		Time?		Time?		
**Blood**							
	Time?		Time?		Time?		
**ECG**							
	Time?		Time?		Time?		
Any others? Please document the tests and who ordered them below							

18. What treatments were given + who ordered them? (respond for each with times)

Treatment	Treating doctor		Another clinician		Ordered at triage		Unsure
	Time?		Time?		Time?		

	Time?		Time?		Time?		
	Time?		Time?		Time?		
	Time?		Time?		Time?		
	Time?		Time?		Time?		
	Time?		Time?		Time?		
	Time?		Time?		Time?		
Any others? Please document the treatments and who ordered them below							

19. Was there any written evidence that the treating doctor discussed any aspect of patient assessment/management with another (or more) senior ED or a specialist clinician?

**Discussed tests/investigations**	**Discussed treatments**	**Neither**
If discussed either - what was written? And where?		

20. What was the patient's presenting complaint(s)/problems?

21. What was the primary diagnosis following assessment?

Was this diagnosis 'suspected' or 'confirmed'? (please tick)			
'suspected'		*'confirmed'*	
Was this patient on any specific pathway? Please give details if so…			
If admitted what were the final discharge diagnoses?			

22. Based on demographics, time of day, comorbidities and presenting symptoms, in your view how difficult and complex would the *decision* to admit or discharge this patient have been? (tick one response per row)

Very difficult		Difficult		Standard		Easy		Very easy	
Very complex		Complex		Standard		Not complex		Not at all complex	

23. After discharge, did the patient re-attend ED within 30 days? (ignore if the patient was not admitted)

**Yes**	**No**
***If so - on what date(s)?* (DD/MM/YY) (multiple allowed)**	
If so – were any related to the initial attendance?	

Yes		No	

If so - with what complaint(s)?

If so - with what diagnoses?

24. After discharge, was the patient admitted to hospital within 30 days? (ignore if not admitted)

Yes	No

If so - on what date(s)? (DD/MM/YY) (multiple allowed)

If so – were any related to the initial attendance?

Yes		No	

If so - with what complaint(s)?

If so - with what diagnoses?

25. After discharge, did the patient die within 30 days? (ignore if not admitted)

Yes	No

If so - on what date? (DD/MM/YY)

If so – was this related to the initial attendance?

Yes		No	

What was the cause of death?

26. Please use this box to note any sections you cannot complete and the reasons why. It may also be used to provide more detail on any item.

[e.g. '*Upon an emergency readmission 4 days after the doctor did not admit the patient, the patient died of causes unrelated to the initial condition. I could only roughly guess at the ASA scores due to incomplete comorbidities documentation.'*]

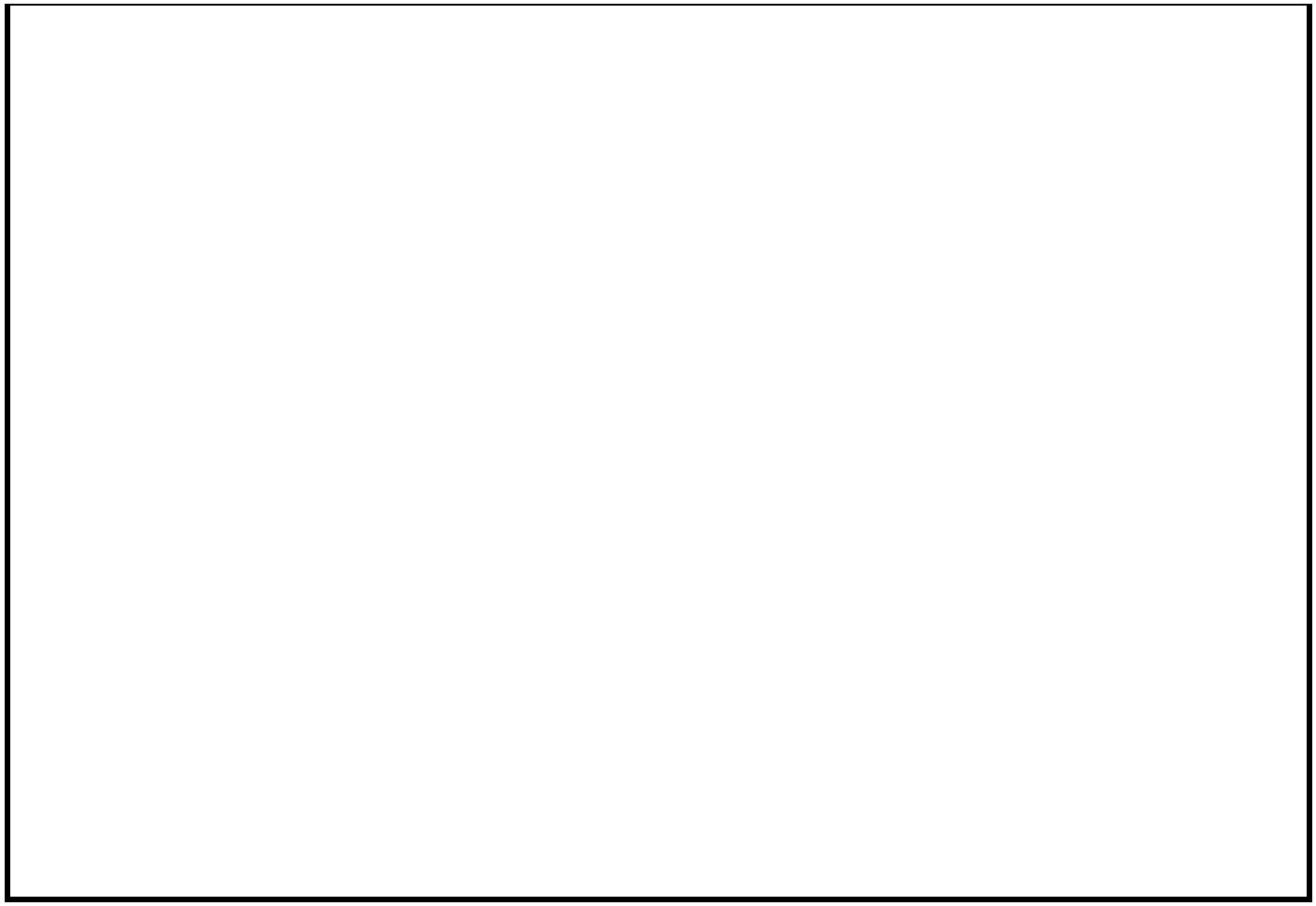

Item guide

1. **What is your extractor ID?**

   Please enter your assigned ID number. This is to link the forms you complete to yourself. This may be used so the research team can reach you and discuss specific patient cases if necessary.

2. **What is the primary treating doctor's ID?**

   Please enter the assigned ID of the participating treating doctor. This is used to link the data extracted in this form to their completed measures, e.g. their tolerance of uncertainty scores or their years of experience.

3. **What site was this patient seen at?**

   Please tick one site only. This will be used to control for site in all analyses, as well as model site specific factors.

4. **Date of presentation**

   This is the date of the initial patient presentation at the emergency department. This will be used for a variety of things but will primarily allow us to assess the time between presentation and discharge, if the patient is admitted. Please enter all dates in the format indicated DD/MM YY.

5. **Mode of arrival**

   If the patient arrived via a 999 road ambulance, please tick the provided box. If they arrived by any other means, please provide detail in the provided text box. The mode of arrival may have implications for their treatment etc. so may be useful to control for in all analyses.

6. **What was the patient's birth date?**

   Please provide this information in DD/MM/YY format. This will be used to control for age in main analyses, which may be a key determinant of the relationship between tolerance of uncertainty and patient health outcomes.

7. **What was the patient's gender?**

Please tick the response as appropriate. This data will be used for a similar purpose as in item 17.

8. **What triage score(s) did the patient receive? (note all that were recorded)**

   Please note all that were recorded in the provided free text boxes. This data will be used for a similar purpose as in item 15.

9. **What pre-existing co-morbidities are listed for this patient?**

   Please provide as much detail as possible in the provided free text box. This data will be used for a similar purpose as in item 15.

10. **Based on comorbidities, in your view this patient was…**

    Please tick your estimated grade as best as you can. It may be that there is not much information on pre-existing co-morbidities, but you suspect they may have or have no significant comorbidities. In this circumstance, please go with your clinical instinct – or note your uncertainty in the last section. ASA scores provide a well validated/reliable and standardised measure of co-morbidities. As in item 15 this will be used to control for the degree to which patients are at risk in all main analyses - which may be a key determinant of the relationship between tolerance of uncertainty and patient health outcomes.

11. **Arrival and assessment times**

    This refers to the specific time at which the patient arrived and the time they were assessed by the treating doctor. Collecting these times will allow us to build a picture of the patient episode, including any delays in treatment. Please enter all times using the 24 hour clock HH:MM.

12. **Was the patient admitted?**

    Please tick yes if so and provide additional details such as the time and specialty in the provided text boxes. Whether the patient was or was not admitted is an important variable in this study as this may reflect the degree to which the treating doctor can tolerate uncertainty.

13. **Was there any written evidence that the treating doctor discussed the decision to admit/discharge with a senior ED or a specialist clinician?**

    Please enter as much detail as you can about what was written and where. This item will be used to control for the fact that some decisions to admit or discharge may not be independent. If it is the case that a treating doctor outsourced their decision to another (or more) senior doctor, this may confound our primary exposure variable of tolerance of uncertainty - so is highly important to capture. In any case, it will be useful to assess whether those with less tolerance of uncertainty defer to others more often, as this may have various important implications.

14. **After assessment and/or treatment where was the patient discharged to?**

    Please tick one option only. Collecting this will allow us to build a picture of the patient episode, including any delays in treatment.

15. **Date of discharge from hospital**

    This is the date of the patient discharge from hospital. If this date was the same as the date of initial presentation, please tick the appropriate checkbox. If not please give the date of discharge in the format DD/MM/YY. Again, discharge may not necessarily be from the emergency department (for instance, if the patient was admitted following their assessment at the emergency department) though it is predicted that the majority of cases will be discharged on the same day as their presentation. This will be used to assess length of stay as an outcome measure.

16. **Discharge time**

    This is the time at which patients were officially discharged (i.e. the planned discharge time), and the time at which they actually left the hospital. Please enter all times using the 24 hour clock HH:MM.

17. **What tests were made and who ordered these?**

    Please provide a response for each test, and more detail on any other tests in the free text box. This is one of our primary outcome measures. It would also be very useful to document any times of these tests. This will be used to assess the correlation between tolerance of uncertainty and resource use (i.e. number of tests ordered). By specifying *who* ordered the tests we can determine whether that resource use is or is not attributable to the participating doctor.

18. **What treatments were given and who ordered these?**

Please provide a response for each treatment, and more detail on any other treatments in the free text box. It would also be very useful to document any times of these tests. As above, this is one of our primary outcome measures. This will be used to assess the correlation between tolerance of uncertainty and resource use (i.e. number of treatments given). By specifying *who* ordered the treatments we can determine whether that resource use is or is not attributable to the participating doctor.

19. Was there any written evidence that the treating doctor discussed any aspect of patient assessment/management with a senior ED or a specialist clinician?

Please enter as much detail as you can about what was written and where. As above, this item will be used to control for the fact that some decisions to order tests or treatments may not be independent in all main analyses. If it is the case that a treating doctor outsourced their decision to another (or more) senior doctor, this may confound our primary exposure variable of tolerance of uncertainty - so is highly important to capture. In any case, it will be useful to assess whether those with less tolerance of uncertainty defer to others more often, as this may have various important implications.

20. What were the patient's presenting complaint(s)/problems?

Please enter as much detail as you can about what was written and where. This will be used to control for the degree to which patients are at risk in all main analyses - which may be a key determinant of the relationship between tolerance of uncertainty and patient health outcomes.

21. What was the primary diagnosis following assessment?

Please provide this information in the free text box with as much detail as necessary. This data will be used for a similar purpose as in item 15.

22. Based on demographics, time of day, comorbidities and presenting symptoms, in your view the *decision* to admit or discharge this patient would have been… (tick one response per row)

This is your judgement about the complexity and difficulty of the decision to admit – please tick one option for complexity and one for difficulty. It was considered useful to assess whether the nature of the decisions made by doctors was comparable across patients. If it were the case, for example, that a large proportion of clinicians saw only non-complex cases conducive to easy

admission decisions (i.e. if there was no uncertainty), tolerance of uncertainty may not be relevant – and there may be no association between tolerance of uncertainty and patient outcomes.

23. Following discharge, did the patient re-attend ED within 30 days?

Please tick the appropriate text box and provide as much detail as necessary in the free text boxes. This will be used as an outcome measure. Reattendance provides *some* indication that *not* admitting a patient in the initial episode was a risky choice, which may be associated with tolerance of uncertainty.

24. Following discharge, was the patient admitted to hospital within 30 days?

As in 23.

25. Following discharge, did the patient die within 30 days?

Please tick the appropriate text box and provide as much detail as necessary in the free text boxes. This will be used as an outcome measure. As with 23 and 24, please only complete this if the patient was admitted in the initial episode.
