# Supplementary material for: Is emergency doctors’ tolerance of clinical uncertainty on a novel measure associated with doctor well-being, healthcare resource use and patient outcomes?
Source: Emerg Med J. 2024 Nov 27;42(1):e213256. doi: 10.1136/emermed-2023-213256 (PMC11874457; doi:10.1136/emermed-2023-213256)
Supplement: online supplemental file 5 [file emermed-42-1-s005.pdf]

Costing materials

As well as having individual indicators of resource use (e.g. total number of tests ordered) and patient outcomes (e.g. return admission), a cost-per-patient outcome was created. This allowed for a numeric/continuous outcome that weighted the relative costs of treatments and tests (e.g. so ordering of oral paracetamol was not statistically equivalent to IV morphine, or a CT scan was not equivalent to standard blood panel), while factoring in other doctors decisions (e.g. costs of patient admission) and costs of the potential *results* of their decisions (e.g. return admissions).

In generating the index, the aim was not to provide an exact estimate of costs, rather to generate a *relative* cost index that allowed for within-sample average episode cost comparisons between doctors.

Three variations of the cost index were analysed. The first simply assessed the cumulative estimated costs of all tests and treatments. The second tests and treatments plus admission costs. The third, which was the primary outcome, assessed all the above plus re-attendance and return admissions costs - or more specifically, the sum of the algorithim in Table 1.

Costs for attendance, return attendance, admission, return admission, and ordered tests, were largely taken from the National Schedule of NHS costs (19/20) estimates. These reference costs account for direct, indirect, and overhead costs, where:

- ***Direct costs*** *- relating directly to the delivery of patient care, e.g. medical staffing costs;*
- ***Indirect costs*** *- indirectly related to the delivery of care, but cannot always be specifically identified to individual patients, e.g. catering and linen; and*
- ***Overhead costs*** *- costs of support services that contribute to the effective running of the organisation, and that cannot be easily attributed to patients, e.g. payroll services*

Costs for ordered medications were largely taken from NICE BNF NHS drug tariff costs. As is common, one assumption was made: that is, it was assumed that a new 'unit' (e.g. box of ampules) was opened per use of each medication, regardless of dose used in practice. Every single decision regarding treatment costing along with alternative costs that could have been used are listed in Table 3.

In several cases data extraction forms provided incomplete data such that - for example - ordering of a specific or generic medication type was noted but without name, route of administration, and/or dose. In order to calculate costs in these cases, all ambiguous cases were reviewed by a consultant emergency doctor who, based on the

patients' reported complaints and other episode information, estimated the incomplete information - before an independent coder matched costs to the estimated treatments (with instruction to be conservative - i.e. pick the lowest costs - where multiple choices were applicable). Another emergency doctor went through the costs to confirm whether they looked accurate and reasonable based on their clinical experience.

Table 1. Costing algorithm with rationale/source of values.

**Indicators**		**Cost estimates**	**Rationale/source**
Admission upon initial presentation:	If attended but not admitted:	**£182**	• Attendance costs (£182) taken from National Schedule of NHS cost (19/20) estimates ('Accident and Emergency' collapsed unit cost). This represents an estimate of the average attendance cost.<br>• Costs contingent on length of stay (e.g. £802 < 2 days) were taken from National Schedule of NHS cost (19/20) average 'short' and 'long' stay costs (non-elective stay). The definitions for 'short' and 'ling' stays were taken from NHS National Cost Collection Guidance (20/21)<br>• Note that costs are only relevant for patients that were admitted by the 'treating doctor' (i.e. the participating doctor under study). Otherwise, the link between participating doctors' UT and cost could not be accurately linked.
	If attended and admitted:	If length of stay < 2 days: **£984 (£182 + £802)**	
		If length of stay > 1 days: **£3701 (£182 + £3519)**	
Tests	If not ordered by the participating doctor:	**£0**	• Test costs were taken from National Schedule of NHS cost (19/20) estimates and summed per case. Specific cost estimates per tests can be seen in Table 2.<br>• Note that one flat rate was used for the ordering of any blood test. This is because data extractors often noted that a blood test had been ordered, without specifying what specific tests. The flat rate was derived as follows. First, for patients for which specific blood tests were noted, the proportion of cases each specific test was ordered for was calculated. These proportions were then multiplied by the cost estimate for each specific blood test and summed. This gave a weighted average that reflected the average cost of ordering a blood test (per se) factoring in the fact that some tests were more expensive, but less frequently ordered
	If ordered by the participating doctor:	**Σ estimated £ of each test**	
Treatments	If not ordered by the participating doctor:	**£0**	• Treatment/medication costs were taken from NICE BNF listed drug tariff prices (or NHS indicative prices where tariff costs were not available) and summed per case. Specific cost estimates can be seen in Table 3.
	If ordered by the participating doctor:	**Σ estimated £ of each treatment**	
30-day re-attendance(s)	If no re-attendance:	**£0**	This cost estimate was taken from attendance costs as described above. Attendances related to the initial complaint were counted only; presentations for unrelated conditions were considered unlikely to be related to doctors' discharge decisions.
	If re-attended:	If not related to the initial complaint: **£0**	
		If related to the initial complaint: **£182 × n re-attendances**	
30-day post-discharge admission(s)	If not admitted:	**£0**	• This cost estimate was taken from attendance costs as described above,
	If admitted:	If not related to the initial complaint: **£0**	

Table 1. Costing algorithm with rationale/source of values.

Indicators	Cost estimates	Rationale/source
	If related to the initial complaint: **£2052 × n admissions**	except that there was just one flat estimate for any admission because return admission length of stay data was not obtained. £2052 = 0.46 × £3519 + 0.54 × £802 where the weights were the proportion of cases who had short (< 2 days) or long (> 1 days) stay lengths upon initial admission (as a best guess of the proportion of patients with short or long stays upon return admission).<br>• Admissions related to the initial complaint were counted only; admissions for unrelated conditions were considered unlikely to be related to doctors' discharge decisions.

Table 2. Test cost estimates

Test type	Test	Cost estimate	Source	Notes
Blood tests	ABG	8.13	Link	Note the flat rate (as described in Table 1 above) was £10.75
	D-Dimer	6.79	Link	
	FBC	2.53	Link	
	UE	1.20	Link	
	Glucose	1.20	Link	
	Chole	1.20	Link	
	Trop	1.20	Link	
	VBG	8.13	Link	
	Coags	29	Link	
	Amylase	1.20	Link	
	CRP	1.20	Link	
	Cultures	8.09	Link	
	ESR	2.53	Link	
	GS	2.53	Link	
	BHCG	1.20	Link	
CT	CT (averaged over various types)	123.37	Link	-
ECG	-	37	Link	-
Bladder scan	USS (averaged over various types)	49.42	Link	-
X-Ray	X-Ray (averaged over various types)	28.62	Link	-
MRI	MRI (averaged over various types)	173.38	Link	-
Misc	Stool culture	8.09	Link	-
	Poct pregnancy test	3	Link	
	Urinalysis	4.08	Link	
	BM	4.50	Link	

Table 3. Costed treatments/medications per patient episode with alternative values (most costs taken from BNF).

Treatments (TD)	Doses/brands etc.	Medication A cost	Medication B cost	Medication C Cost	Medication D Cost	Medication E Cost	Medication F cost	Total cost	Notes
IV paracetamol<br>IV antiemetic	IV paracetamol 1g<br>IV ondansetron 4mg	14.40 (12 vials)	10.00 for 10 ampules (tarriff cost: 18.70)					24.40	Medicine A alt costs (NHS indicative):<br>14.40 for 12 vials<br>24 for 20 vials<br>17.90 for 10 vials (Tarriff 12)<br>Medicine B alt costs:<br>37.11 for 5 ampules (tarriff: 29.97)<br>18.70 for 10 ampules (tarrif cost: 18.70)<br>28.47 for 5 ampules (tarriff: 29.97)<br>5.40 for 5 ampules (tarrif: 29.97)<br>29.20 for 5 ampules (tarrif: 29.97)<br>5.80 for 5 ampules (tarrif cost: 29.97)<br>18.70 for 10 ampules (tarrif cost: 18.70).<br>decision: cheapest per ampule
na								0	
IV antibiotics<br>IV antiemetics<br>IV morphine	IV cefuroxine 1.5g and metronidazole 500mg<br>IV ondansetron 4mg<br>IV morphine 10mg	50.50 (10 vials)	68.39 bag, 30 (tariff cost: 68.39)	10.00 for 10 ampules (tarriff cost: 18.70)	11.44 (10 ampoules, tariff cost: 11.45)			140.33	Medicine A costs:<br>5.05 (1 vial, hospital only)<br>50.50 (10 vials, hospital only)<br>57.90 (10 vials, hospital only)<br>4.70 (1 vial, hospital only)<br>rationale: only available option outside hospital<br>Medicine B: no alternative IV costs, assuming IV was intended.<br>Medicine C alt costs:<br>37.11 for 5 ampules (tarriff: 29.97)<br>18.70 for 10 ampules (tarrif cost: 18.70)<br>28.47 for 5 ampules (tarriff: 29.97)<br>5.40 for 5 ampules (tarrif: 29.97)<br>29.20 for 5 ampules (tarrif: 29.97)<br>5.80 for 5 ampules (tarrif cost: 29.97)<br>18.70 for 10 ampules (tarrif cost: 18.70).<br>decision: cheapest per ampule<br>Medicine D alternative costs:<br>11.45 (tariff cost: 11.45)<br>11.87 (tariff cost: 11.45)<br>14 (tariff cost: 11.45)

GTN<br>IV morphine<br>IV antiemetic	Glyceryl trinate tablet<br>IV morphine 10mg<br>IV ondansetron 4mg	1.89 (100, tariff cost 1.89)	11.44 (10 ampoules, tariff cost: 11.45)	10.00 for 10 ampules (tarriff cost: 18.70)				23.43	medicine A alt costs:<br>1.97 (tariff price 1.89)<br>2.03 (tariff price 1.89)<br>Medicine B alternative costs:<br>11.45 (tariff cost: 11.45)<br>11.87 (tariff cost: 11.45)<br>14 (tariff cost: 11.45)<br>Medicine C alt costs:<br>37.11 for 5 ampules (tarriff: 29.97)<br>18.70 for 10 ampules (tarrif cost: 18.70)<br>28.47 for 5 ampules (tarriff: 29.97)<br>5.40 for 5 ampules (tarrif: 29.97)<br>29.20 for 5 ampules (tarrif: 29.97)<br>5.80 for 5 ampules (tarrif cost: 29.97)<br>18.70 for 10 ampules (tarrif cost: 18.70).<br>decision: cheapest per ampule
TTO antibiotic	oral nitrofurantoin course 50mg	4.20 (28 tablets, tariff 4.20)						4.20	Medicine A alt costs:<br>7.51 (28 tablets, tariff 4.20)<br>31.33 (28 tablets, tariff 4.20)<br>5.08 (28 tablets, tariff 4.20)<br>7.64 (28 tablets, tariff 4.20)<br>26.31 (100 tablets)<br>18.14(100 tablets)<br>111.89 (100 tablets)
na								0	
IV paracetamol	IV paracetamol	14.40 (12 vials)						14.40	Medicine A alt costs (NHS indicative):<br>14.40 for 12 vials<br>24 for 20 vials<br>17.90 for 10 vials (Tarriff 12)
na								0	

TTO omeprazole	omeprazole course	.66 (7 capsules, .7 tariff price)						0.6	medicine A alt costs:<br>8.35 (7 capsules, tariff .7)<br>.8(7 capsules, tariff .7)<br>.7(7 capsules, tariff .7)<br>4.93(7 capsules, tariff .7)<br>.66(7 capsules, tariff .7)<br>2.37(7 capsules, tariff .7)<br>9.69(7 capsules, tariff .7)<br>3 (28 capsules)<br>9 (28 capsules)<br>9.48 (28 capsules)<br>2.64 (28 capsules)<br>3.2 (28 capsules)<br>19.72 (28 capsules)<br>26.72 (28 capsules)<br>2.8 (28 capsules)<br>3.19 (28 capsules)
na								0	
IV hartmann's<br>Oral prochlorperazine<br>Oral paracetamol	IV hartmann solution<br>Oral prochlorperazine<br>Oral paracetamol	3.95	3.34 (5mg/ml, 100ml, tariff price 3.34)	.13 (16 tablets)				7.42	Medicine A no info available, used: https://www.medekit.com/drugs/intravenous-fluids/hartmanns-solution-500ml-500-mlt-6587/<br>Medicine B no alt cost available<br>Medicine A alt costs:<br>.93 (8 tablets)<br>.86 (12 tablets)<br>1.42 (14 tablets)<br>1.04 (16 tablets)<br>.76 (16 tablets)<br>.12 (16 tablets)<br>.27 (16 tablets)<br>.72 (16 tablets)<br>.18 (16 tablets)<br>.43 (16 tablets)<br>.26 (16 tablets)<br>.36 (16 tablets)<br>.34 (16 tablets)<br>.38 (16 tablets)<br>.17 (16 tablets)<br>.19 (16 tablets)<br>.31 (16 tablets)<br>.45 (16 tablets)<br>.67 (20 tablets)<br>.48 (20 tablets)<br>.54 (20 tablets)<br>.82 (30 tablets)<br>.15 (32 tablets tariff 0.76)<br>.62 (32 tablets tariff 0.76)<br>.2 (32 tablets tariff 0.76)<br>1.92 (32 tablets tariff 0.76)<br>.89 (32 tablets tariff 0.76)<br>.76 (32 tablets tariff 0.76)<br>1.44 (32 tablets tariff 0.76)<br>.33 (32 tablets tariff 0.76)<br>.68 (32 tablets tariff 0.76)<br>.52 (32 tablets tariff 0.76)<br>.49 (32 tablets tariff 0.76)<br>.29 (32 tablets tariff 0.76)<br>1.34 (100 tablets tariff 2.38)<br>1.95 (100 tablets tariff 2.38)<br>1.62 (100 tablets tariff 2.38)<br>3.5 (100 tablets tariff 2.38)<br>2.78 (100 tablets tariff 2.38)<br>2.38 (100 tablets tariff 2.38)<br>4.5 (100 tablets tariff 2.38)

				3.05 (100 tablets tariff 2.38)<br>2.5 (100 tablets tariff 2.38)<br>2.17 (100 tablets tariff 2.38)<br>2.2 (100 tablets tariff 2.38)<br>1.84 (100 tablets tariff 2.38)<br>2.53 (100 tablets tariff 2.38)<br>.6 (100 tablets tariff 2.38)<br>2.78 (100 tablets tariff 2.38)<br>3.25 (100 tablets tariff 2.38)<br>23.8 (1000 tablets)

IV paracetamol<br>IV ondansetron<br>IV morphine<br>Oral codine	IV paracetamol<br>IV ondansetron<br>IV morphine<br>Oral codeine phosphate 60mg	14.40 (12 vials)	10.00 for 10 ampules (tarriff cost: 18.70)	11.44 (10 ampoules, tariff cost: 11.45)	1.71 200ml			37.55	Medicine A alt costs (NHS indicative):<br>14.40 for 12 vials<br>24 for 20 vials<br>17.90 for 10 vials (Tarriff 12)<br>Medicine B alt costs:<br>37.11 for 5 ampules (tarriff: 29.97)<br>18.70 for 10 ampules (tarrif cost: 18.70)<br>28.47 for 5 ampules (tarriff: 29.97)<br>5.40 for 5 ampules (tarrif: 29.97)<br>29.20 for 5 ampules (tarrif: 29.97)<br>5.80 for 5 ampules (tarrif cost: 29.97)<br>18.70 for 10 ampules (tarrif cost: 18.70).<br>decision: cheapest per ampule<br>Medicine C alternative costs:<br>11.45 (tariff cost: 11.45)<br>11.87 (tariff cost: 11.45)<br>14 (tariff cost: 11.45)<br>medicine D alt costs:<br>1.90 (tariff: 1.90)<br>1.73 (tariff: 1.90)<br>16.80 for 2000ml<br>9.90 for 2000ml
Oral cocodamol	Oral cocodamol (30/500)	11.99 (tariff cost: 11.99)						11.99	Medicine A alt costs: no oral options
na								0	

Oral ibuprofen<br>Oral co-codamol	Oral ibuprofen (400mg)<br>Oral co-codamol (30/500)	0.56 (24 tablets, tariff price 1.03)	11.99 (tariff cost: 11.99)					12.55	Medicine A alt costs:<br>1.15 (48 tablets, tariff price 1.03)<br>2.05 (96 tablets, tariff price 1.03)<br>4.9 (60 tablets, 1.03 tariff)<br>2.06 (24 tablets, 1,03 tariff)<br>3.49 (48 tablets, 1.03 tariff)<br>5.49 (96 tablets, 1.03 tariff)<br>2.8 (84 tablets, 3.61 tariff)<br>1.39 (24 tablets, 1.03 tariff)<br>2.56 (48 tablets)<br>4.1 (84 tablets, 3.61 tariff)<br>1.03 (24 tablets, 1.03 tariff)<br>2.06 (48 tablets)<br>3.61 (84 tablets, 3.61 tariff)<br>6.14 (84 tablets, 3.61 tariff)<br>0.7 (24 tablets, 1.03 tariff)<br>0.85 (48 tablets)<br>1 (84 tablets, 3.61 tariff)<br>3 (96 tablets)<br>2.71, (84 tablets, 3.61 tariff)<br>5.74 (84 tablets, 3.61 tariff)<br>0.9 (24 tablets, 1.03 tariff)<br>3.01 (84 tablets, 3.61 tariff)<br>0.7 (24 tablets, 1.03 tariff)<br>1.17 (48 tablets)<br>2 (96 tablets)<br>Medicine B alt costs: none

Oral paracetamol<br>IV morphine	Oral paracetamol (1g)<br>IV morphine (10mg)	.13 (16 tablets)	11.44 (10 ampoules, tariff cost: 11.45)					11.57	Medicine A alt costs:<br>.93 (8 tablets)<br>.86 (12 tablets)<br>1.42 (14 tablets)<br>1.04 (16 tablets)<br>.76 (16 tablets)<br>.12 (16 tablets)<br>.27 (16 tablets)<br>.72 (16 tablets)<br>.18 (16 tablets)<br>.43 (16 tablets)<br>.26 (16 tablets)<br>.36 (16 tablets)<br>.34 (16 tablets)<br>.38 (16 tablets)<br>.17 (16 tablets)<br>.19 (16 tablets)<br>.31 (16 tablets)<br>.45 (16 tablets)<br>.67 (20 tablets)<br>.48 (20 tablets)<br>.54 (20 tablets)<br>.82 (30 tablets)<br>.15 (32 tablets tariff 0.76)<br>.62 (32 tablets tariff 0.76)<br>.2 (32 tablets tariff 0.76)<br>1.92 (32 tablets tariff 0.76)<br>.89 (32 tablets tariff 0.76)<br>.76 (32 tablets tariff 0.76)<br>1.44 (32 tablets tariff 0.76)<br>.33 (32 tablets tariff 0.76)<br>.68 (32 tablets tariff 0.76)<br>.52 (32 tablets tariff 0.76)<br>.49 (32 tablets tariff 0.76)<br>.29 (32 tablets tariff 0.76)<br>1.34 (100 tablets tariff 2.38)<br>1.95 (100 tablets tariff 2.38)<br>1.62 (100 tablets tariff 2.38)<br>3.5 (100 tablets tariff 2.38)<br>2.78 (100 tablets tariff 2.38)<br>2.38 (100 tablets tariff 2.38)<br>4.5 (100 tablets tariff 2.38)<br>3.05 (100 tablets tariff 2.38)<br>2.5 (100 tablets tariff 2.38)<br>2.17 (100 tablets tariff 2.38)<br>2.2 (100 tablets tariff 2.38)<br>1.84 (100 tablets tariff 2.38)<br>2.53 (100 tablets tariff 2.38)<br>.6 (100 tablets tariff 2.38)<br>2.78 (100 tablets tariff 2.38)<br>3.25 (100 tablets tariff 2.38)<br>23.8 (1000 tablets)<br>Medicine B alternative costs:<br>11.45 (tariff cost: 11.45)<br>11.87 (tariff cost: 11.45)<br>14 (tariff cost: 11.45)

Oral cocodamol<br>Oral ibuprofen	Oral cocodamol (30/500)<br>Oral ibuprofen (400mg)	11.99 (tariff cost: 11.99)	0.56 (24 tablets, tariff price 1.03)					12.55	Medicine A alt costs: none<br>Medicine B alt costs:<br>1.15 (48 tablets, tariff price 1.03)<br>2.05 (96 tablets, tariff price 1.03)<br>4.9 (60 tablets, 1.03 tariff)<br>2.06 (24 tablets, 1,03 tariff)<br>3.49 (48 tablets, 1.03 tariff)<br>5.49 (96 tablets, 1.03 tariff)<br>2.8 (84 tablets, 3.61 tariff)<br>1.39 (24 tablets, 1.03 tariff)<br>2.56 (48 tablets)<br>4.1 (84 tablets, 3.61 tariff)<br>1.03 (24 tablets, 1.03 tariff)<br>2.06 (48 tablets)<br>3.61 (84 tablets, 3.61 tariff)<br>6.14 (84 tablets, 3.61 tariff)<br>0.7 (24 tablets, 1.03 tariff)<br>0.85 (48 tablets)<br>1 (84 tablets, 3.61 tariff)<br>3 (96 tablets)<br>2.71, (84 tablets, 3.61 tariff)<br>5.74 (84 tablets, 3.61 tariff)<br>0.9 (24 tablets, 1.03 tariff)<br>3.01 (84 tablets, 3.61 tariff)<br>0.7 (24 tablets, 1.03 tariff)<br>1.17 (48 tablets)<br>2 (96 tablets)
na								0	
na								0	
Propranolol<br>Carbimazole	Propranolol hydrochloride10mg<br>Carbimazole 15mg	0.96 (28 tablets, 1 tariff)	115.98 (100 tablets, tariff 127.03					116.84	Medicine A alt cost:<br>1 (28 tabletts, 1 tariff)<br>7 (28 tabletts, 1 tariff)<br>1.38 (28 tabletts, 1.05 tariff)<br>1.53 (28 tabletts, 1.05 tariff)<br>0.98 (28 tabletts, 1.05 tariff)<br>1.05 (28 tabletts, 1.05 tablett)<br>7 (28 tablets, 1.05 tariff)<br>Medicine B alt costs:<br>127.03 (100 tablets, tariff 127.03
TTO antibiotics	Augmetin 625mg course	9.60 (21 tablets, tariff 2.09)						9.6	Medicin A no alt costs
na								0	
na								0	
na								0	
na								0	

IV fluids<br>IV paracetamol<br>Oral codeine	IV fluids (likely N/saline)<br>IV paracetamol 1g<br>Oral codeine phopshate	2.62 Sodium chloride 0.9% (1l bottle, tariff 3.96)	14.40 (12 vials)	1.71 200ml				18.73	Medicine A alt cost:<br>4.12 1l bottle<br>24.72 6x 1lbottle<br>3.1 500ml bottle<br>37.2 12x500ml bottle<br>0.59 100ml bottle<br>11.78 20x 100ml bottle<br>2.62 1l bottle<br>26.2 10x 1l bottle<br>13.25 10x1l bottle<br>6.13 10x 250ml bottle<br>2.38 500ml bottle<br>23.80 10x 500ml bottle<br>13.25 500ml bottle<br>11.78 50ml bottle<br>Medicine B alt costs (NHS indicative):<br>14.40 for 12 vials<br>24 for 20 vials<br>17.90 for 10 vials (Tarriff 12)<br>medicine D alt costs:<br>1.90 (tariff: 1.90)<br>1.73 (tariff: 1.90)<br>16.80 for 2000ml<br>9.90 for 2000ml
TTO antibiotics	Augmetin 625mg course	9.60 (21 tablets, tariff 2.09)						9.6	Medicin A no alt costs
na								0	
na								0	

Sumatriptan<br>IV paracetamol<br>IV antiemetic	Sumatriptan 50mg oral<br>IV paracetamol 1g<br>IV ondansetron	1.14 (6 tablets, tariff 1.14)	14.40 (12 vials)	10.00 for 10 ampules (tarriff cost: 18.70)				25.54	Medicine A alt cost:<br>1.94 (6 tablets, tariff 1.14)<br>31.85 (6 tablets, tariff 1.14<br>31 (6 tablets, tariff 1.14)<br>16.60 (6 tablets, tariff 1.14)<br>22.56 (6 tablets, tariff 1.14)<br>24.85 (6 tablets, tariff 11.14)<br>1.34 (6 tablets, tariff 1.14)<br>1.35 (6 tablets, tariff 1.14<br>4.44 (6 tablets)<br>23.9 (6 tablets, tariff 1.14)Medicine B alt costs (NHS indicative):<br>14.40 for 12 vials<br>24 for 20 vials<br>17.90 for 10 vials (Tarriff 12)<br>Medicine C alt costs:<br>37.11 for 5 ampules (tarriff: 29.97)<br>18.70 for 10 ampules (tarrif cost: 18.70)<br>28.47 for 5 ampules (tarriff: 29.97)<br>5.40 for 5 ampules (tarrif: 29.97)<br>29.20 for 5 ampules (tarrif: 29.97)<br>5.80 for 5 ampules (tarrif cost: 29.97)<br>18.70 for 10 ampules (tarrif cost: 18.70).<br>decision: cheapest per ampule
TTO cocodamol	Cocodamol (30/500) course	11.99 (tariff cost: 11.99)						11.99	Medicine A alt costs: no oral options
na								0	
Ticagrelor<br>Aspirin<br>Fondaparinux	Ticagrelor 180mg<br>Aspirin 300mg<br>Fondaparinux (subcutaenous)	54.6 (90mg, tariff price 54.6)	0.23 (32 tablets, tariff 3.88)	62.79 (10 tablets, 2.5mg tariff 62.79)				117.62	medicine A: 180 not available, 90x2 is. No alternative costs.<br>alternative costs medicine B:<br>3.54 (28 tablets)<br>12.54 (100 tablets, tariff 12.11)<br>3.88 (32 tablets, tariff 3.88)<br>0.28 (32 tablets, tariff 3.88)
IV lorazepam	IV lorazepam 2mg	69 (10 ampoules of 4mg)						69	IV currently unavailable at 2mg.<br>This was the only IV Lorazepam available
na								0	

Oral analgesia	Paracetamol 1g (oral)	.13 (16 tablets)						.13	Medicine A alt costs:<br>.93 (8 tablets)<br>.86 (12 tablets)<br>1.42 (14 tablets)<br>1.04 (16 tablets)<br>.76 (16 tablets)<br>.12 (16 tablets)<br>.27 (16 tablets)<br>.72 (16 tablets)<br>.18 (16 tablets)<br>.43 (16 tablets)<br>.26 (16 tablets)<br>.36 (16 tablets)<br>.34 (16 tablets)<br>.38 (16 tablets)<br>.17 (16 tablets)<br>.19 (16 tablets)<br>.31 (16 tablets)<br>.45 (16 tablets)<br>.67 (20 tablets)<br>.48 (20 tablets)<br>.54 (20 tablets)<br>.82 (30 tablets)<br>.15 (32 tablets tariff 0.76)<br>.62 (32 tablets tariff 0.76)<br>.2 (32 tablets tariff 0.76)<br>1.92 (32 tablets tariff 0.76)<br>.89 (32 tablets tariff 0.76)<br>.76 (32 tablets tariff 0.76)<br>1.44 (32 tablets tariff 0.76)<br>.33 (32 tablets tariff 0.76)<br>.68 (32 tablets tariff 0.76)<br>.52 (32 tablets tariff 0.76)<br>.49 (32 tablets tariff 0.76)<br>.29 (32 tablets tariff 0.76)<br>1.34 (100 tablets tariff 2.38)<br>1.95 (100 tablets tariff 2.38)<br>1.62 (100 tablets tariff 2.38)<br>3.5 (100 tablets tariff 2.38)<br>2.78 (100 tablets tariff 2.38)<br>2.38 (100 tablets tariff 2.38)<br>4.5 (100 tablets tariff 2.38)<br>3.05 (100 tablets tariff 2.38)<br>2.5 (100 tablets tariff 2.38)<br>2.17 (100 tablets tariff 2.38)<br>2.2 (100 tablets tariff 2.38)<br>1.84 (100 tablets tariff 2.38)<br>2.53 (100 tablets tariff 2.38)<br>.6 (100 tablets tariff 2.38)<br>2.78 (100 tablets tariff 2.38)<br>3.25 (100 tablets tariff 2.38)<br>23.8 (1000 tablets)
IV antibiotics	IV augmentin 1.2g	10.60 (1000 + 200mg vial Amoxicillin)						10.6	Closest dosage to 1.2g IV; 1000mg vial Amoxicillin + 200mg patassium clavulanate. Alt costs:<br>29.70 (1000+200mg)<br>10.96 (1000+200mg)<br>50 (1000+200mg)<br>27.50 (1000+200mg)
na								0	

Oral antiemetic<br>TTO antibiotics<br>Oral analgesia	Oral ondansetron<br>Oral nitrofurantoin (course)<br>Oral paracetamol	30,55 (4mg/5ml: 50ml tariff 38.37)	4.20 (28 tablets, tariff 4.20)	.13 (16 tablets)				34.88	Medicine A unclear dosage 4mg/5ml was the only dosage available for oral solution, hence this was chosen. alt costs:<br>38.36 (4mg/5ml: 50ml tariff 38.37)<br>35.97 (4mg/5ml: 50ml tariff 38.37)<br>38.37 (4mg/5ml: 50ml tariff 38.37)<br>37.92 (4mg/5ml: 50ml tariff 38.37)<br>18 (4mg/5ml: 50ml tariff 38.37)<br>Medicine B alt costs:<br>7.51 (28 tablets, tariff 4.20)<br>31.33 (28 tablets, tariff 4.20)<br>5.08 (28 tablets, tariff 4.20)<br>7.64 (28 tablets, tariff 4.20)<br>26.31 (100 tablets)<br>18.14(100 tablets)<br>111.89 (100 tablets)<br>Medicine A alt costs:<br>.93 (8 tablets)<br>.86 (12 tablets)<br>1.42 (14 tablets)<br>1.04 (16 tablets)<br>.76 (16 tablets)<br>.12 (16 tablets)<br>.27 (16 tablets)<br>.72 (16 tablets)<br>.18 (16 tablets)<br>.43 (16 tablets)<br>.26 (16 tablets)<br>.36 (16 tablets)<br>.34 (16 tablets)<br>.38 (16 tablets)<br>.17 (16 tablets)<br>.19 (16 tablets)<br>.31 (16 tablets)<br>.45 (16 tablets)<br>.67 (20 tablets)<br>.48 (20 tablets)<br>.54 (20 tablets)<br>.82 (30 tablets)<br>.15 (32 tablets tariff 0.76)<br>.62 (32 tablets tariff 0.76)<br>.2 (32 tablets tariff 0.76)<br>1.92 (32 tablets tariff 0.76)<br>.89 (32 tablets tariff 0.76)<br>.76 (32 tablets tariff 0.76)<br>1.44 (32 tablets tariff 0.76)<br>.33 (32 tablets tariff 0.76)<br>.68 (32 tablets tariff 0.76)<br>.52 (32 tablets tariff 0.76)<br>.49 (32 tablets tariff 0.76)<br>.29 (32 tablets tariff 0.76)<br>1.34 (100 tablets tariff 2.38)<br>1.95 (100 tablets tariff 2.38)<br>1.62 (100 tablets tariff 2.38)<br>3.5 (100 tablets tariff 2.38)<br>2.78 (100 tablets tariff 2.38)<br>2.38 (100 tablets tariff 2.38)<br>4.5 (100 tablets tariff 2.38)<br>3.05 (100 tablets tariff 2.38)<br>2.5 (100 tablets tariff 2.38)<br>2.17 (100 tablets tariff 2.38)<br>2.2 (100 tablets tariff 2.38)<br>1.84 (100 tablets tariff 2.38)<br>2.53 (100 tablets tariff 2.38)<br>.6 (100 tablets tariff 2.38)<br>2.78 (100 tablets tariff 2.38)<br>3.25 (100 tablets tariff 2.38)<br>23.8 (1000 tablets)

IV analgesia<br>IV antibiotics	IV morphine<br>IV tazocin 4.5g	11.44 (10 ampoules, tariff cost: 11.45)	12.90 (1 vial)					24.34	Medicine A alternative costs:<br>11.45 (tariff cost: 11.45)<br>11.87 (tariff cost: 11.45)<br>14 (tariff cost: 11.45)<br>Medicine B alt costs:<br>15.75 (1 vial)<br>15.17(1 vial)<br>19.97 (1 vial)<br>36.50 (10 vials)<br>76.50 (10 vials)<br>48 (10 vials)<br>25 (10 vials)<br>99 (10 vials)
na								0	
Analgesia<br>Antiemetic	IV morphine 10mg<br>IV ondansetron 4mg	11.44 (10 ampoules, tariff cost: 11.45)	10.00 for 10 ampules (tarriff cost: 18.70)					21.44	Medicine A alternative costs:<br>11.45 (tariff cost: 11.45)<br>11.87 (tariff cost: 11.45)<br>14 (tariff cost: 11.45)<br>Medicine B alt costs:<br>37.11 for 5 ampules (tarriff: 29.97)<br>18.70 for 10 ampules (tarrif cost: 18.70)<br>28.47 for 5 ampules (tarriff: 29.97)<br>5.40 for 5 ampules (tarrif: 29.97)<br>29.20 for 5 ampules (tarrif: 29.97)<br>5.80 for 5 ampules (tarrif cost: 29.97)<br>18.70 for 10 ampules (tarrif cost: 18.70).<br>decision: cheapest per ampule
na								0	
Gaviscon	Gaviscon	2.58 (150ml, tariff 2.58)						2.58	Medicine A alt cost:<br>4.33 (300ml tariff 4.33)<br>7.11 (600ml tariff 7.11)
ACS medicines<br>GTN	Ticagrelor 180mg<br>Aspirin 300mg<br>Fondaparinux (subcutaenous)<br>GTN tablet	54.6 (90mg, tariff price 54.6)	0.23 (32 tablets, tariff 3.88)	62.79 (10 tablets, 2.5mg tariff 62.79)	1.89 (100, tariff cost 1.89)			119.51	medicine A: 180 not available, 90x2 is. No alternative costs.<br>alternative costs medicine B:<br>3.54 (28 tablets)<br>12.54 (100 tablets, tariff 12.11)<br>3.88 (32 tablets, tariff 3.88)<br>0.28 (32 tablets, tariff 3.88)<br>Medicin C: no alternative costs<br>medicine D alt costs:<br>1.97 (tariff price 1.89)<br>2.03 (tariff price 1.89)

na	Unsure re cost of oxygen	8.96 (340l)						8.96	medicine A alternative cost:<br>17.77 (300l)<br>12 (460l)<br>21.62 (600l)<br>9.77 (680l)<br>11.39 (1360l)<br>12.23 (1360l)<br>17.23 (2300l)<br>15.31 (3400l)<br>24.04 (3040l)<br>21.62 (6800l)<br>37.61 (11300l) information gathered from: https://www.boconline.co.uk/shop/en/uk/gas-a-z/oxygen/oxygen-cylinder-medical-grade-compressed-gas
na								0	
na								0	
na								0	
Paracetamol	Oral paracetamol (1g)	.13 (16 tablets, 500mg)						.13	Medicine A alt costs:<br>.93 (8 tablets)<br>.86 (12 tablets)<br>1.42 (14 tablets)<br>1.04 (16 tablets)<br>.76 (16 tablets)<br>.12 (16 tablets)<br>.27 (16 tablets)<br>.72 (16 tablets)<br>.18 (16 tablets)<br>.43 (16 tablets)<br>.26 (16 tablets)<br>.36 (16 tablets)<br>.34 (16 tablets)<br>.38 (16 tablets)<br>.17 (16 tablets)<br>.19 (16 tablets)<br>.31 (16 tablets)<br>.45 (16 tablets)<br>.67 (20 tablets)<br>.48 (20 tablets)<br>.54 (20 tablets)<br>.82 (30 tablets)<br>.15 (32 tablets tariff 0.76)<br>.62 (32 tablets tariff 0.76)<br>.2 (32 tablets tariff 0.76)<br>1.92 (32 tablets tariff 0.76)<br>.89 (32 tablets tariff 0.76)<br>.76 (32 tablets tariff 0.76)<br>1.44 (32 tablets tariff 0.76)<br>.33 (32 tablets tariff 0.76)<br>.68 (32 tablets tariff 0.76)<br>.52 (32 tablets tariff 0.76)<br>.49 (32 tablets tariff 0.76)<br>.29 (32 tablets tariff 0.76)<br>1.34 (100 tablets tariff 2.38)<br>1.95 (100 tablets tariff 2.38)<br>1.62 (100 tablets tariff 2.38)<br>3.5 (100 tablets tariff 2.38)<br>2.78 (100 tablets tariff 2.38)<br>2.38 (100 tablets tariff 2.38)<br>4.5 (100 tablets tariff 2.38)<br>3.05 (100 tablets tariff 2.38)<br>2.5 (100 tablets tariff 2.38)<br>2.17 (100 tablets tariff 2.38)<br>2.2 (100 tablets tariff 2.38)

									1.84 (100 tablets tariff 2.38)<br>2.53 (100 tablets tariff 2.38)<br>.6 (100 tablets tariff 2.38)<br>2.78 (100 tablets tariff 2.38)<br>3.25 (100 tablets tariff 2.38)<br>23.8 (1000 tablets)
Augmentin	IV augmentin 1.2g	10.60 (1000 + 200mg vial Amoxicillin)						10.6	Closest dosage to 1.2g IV; 1000mg vial Amoxicillin + 200mg patassium clavulanate. Alt costs:<br>29.70 (1000+200mg)<br>10.96 (1000+200mg)<br>50 (1000+200mg)<br>27.50 (1000+200mg)
na								0	
na								0	
na								0	
na								0	
na	Dihydrocodeine with paracetamol	2.49 (30 tablets, tariff 1.69)						0	10mg/500mg tablets most common Medicine A alt costs:<br>2.50 (30 tablets, tariff 1.69)<br>3.24(30 tablets, tariff 1.69)<br>4.50 (100 tablets, tariff 5.63)<br>7.59(100 tablets, tariff 5.63)<br>8.30(100 tablets, tariff 5.63)<br>9.75(100 tablets, tariff 5.63)<br>28.15 (500 tablets

Labetalol	IV labetalol hydrochloride 50mg	3.79 (56 tablets tariff 3.79)						3.79	Medicine A no alt costs
Co-amoxiclav	IV augmentin 1.2g	10.60 (1000 + 200mg vial Amoxicillin)						10.6	Closest dosage to 1.2g IV; 1000mg vial Amoxicillin + 200mg patassium clavulanate. Alt costs:<br>29.70 (1000+200mg)<br>10.96 (1000+200mg)<br>50 (1000+200mg)<br>27.50 (1000+200mg)
na								0	
na								0	
Analgesia (morphine, paracetamol)	IV morphine 10mg<br>oral paracetamol 1g	11.44 (10 ampoules, tariff cost: 11.45)	.13 (16tablets, 500mg)					11.57	Medicine A alternative costs:<br>11.45 (tariff cost: 11.45)<br>11.87 (tariff cost: 11.45)<br>14 (tariff cost: 11.45)<br>Medicine B alt costs:<br>.93 (8 tablets)<br>.86 (12 tablets)<br>1.42 (14 tablets)<br>1.04 (16 tablets)<br>.76 (16 tablets)<br>.12 (16 tablets)<br>.27 (16 tablets)<br>.72 (16 tablets)<br>.18 (16 tablets)<br>.43 (16 tablets)<br>.26 (16 tablets)<br>.36 (16 tablets)<br>.34 (16 tablets)<br>.38 (16 tablets)<br>.17 (16 tablets)<br>.19 (16 tablets)<br>.31 (16 tablets)<br>.45 (16 tablets)<br>.67 (20 tablets)<br>.48 (20 tablets)<br>.54 (20 tablets)<br>.82 (30 tablets)<br>.15 (32 tablets tariff 0.76)<br>.62 (32 tablets tariff 0.76)<br>.2 (32 tablets tariff 0.76)<br>1.92 (32 tablets tariff 0.76)<br>.89 (32 tablets tariff 0.76)<br>.76 (32 tablets tariff 0.76)<br>1.44 (32 tablets tariff 0.76)<br>.33 (32 tablets tariff 0.76)<br>.68 (32 tablets tariff 0.76)<br>.52 (32 tablets tariff 0.76)<br>.49 (32 tablets tariff 0.76)<br>.29 (32 tablets tariff 0.76)<br>1.34 (100 tablets tariff 2.38)<br>1.95 (100 tablets tariff 2.38)<br>1.62 (100 tablets tariff 2.38)<br>3.5 (100 tablets tariff 2.38)<br>2.78 (100 tablets tariff 2.38)<br>2.38 (100 tablets tariff 2.38)<br>4.5 (100 tablets tariff 2.38)<br>3.05 (100 tablets tariff 2.38)<br>2.5 (100 tablets tariff 2.38)<br>2.17 (100 tablets tariff 2.38)<br>2.2 (100 tablets tariff 2.38)<br>1.84 (100 tablets tariff 2.38)<br>2.53 (100 tablets tariff 2.38)<br>.6 (100 tablets tariff 2.38)<br>2.78 (100 tablets tariff 2.38)<br>3.25 (100 tablets tariff 2.38)<br>23.8 (1000 tablets)
na								0	
na								0	
na								0	

na								0	
na								0	
na								0	
na								0	
na								0	
na								0	
Analgesia	oral paracetamol		.13 (16 tablets, 500mg)					.13	Medicine A alt costs:<br>.93 (8 tablets)<br>.86 (12 tablets)<br>1.42 (14 tablets)<br>1.04 (16 tablets)<br>.76 (16 tablets)<br>.12 (16 tablets)<br>.27 (16 tablets)<br>.72 (16 tablets)<br>.18 (16 tablets)<br>.43 (16 tablets)<br>.26 (16 tablets)<br>.36 (16 tablets)<br>.34 (16 tablets)<br>.38 (16 tablets)<br>.17 (16 tablets)<br>.19 (16 tablets)<br>.31 (16 tablets)<br>.45 (16 tablets)<br>.67 (20 tablets)<br>.48 (20 tablets)<br>.54 (20 tablets)<br>.82 (30 tablets)<br>.15 (32 tablets tariff 0.76)<br>.62 (32 tablets tariff 0.76)<br>.2 (32 tablets tariff 0.76)<br>1.92 (32 tablets tariff 0.76)<br>.89 (32 tablets tariff 0.76)<br>.76 (32 tablets tariff 0.76)<br>1.44 (32 tablets tariff 0.76)<br>.33 (32 tablets tariff 0.76)<br>.68 (32 tablets tariff 0.76)<br>.52 (32 tablets tariff 0.76)<br>.49 (32 tablets tariff 0.76)<br>.29 (32 tablets tariff 0.76)<br>1.34 (100 tablets tariff 2.38)<br>1.95 (100 tablets tariff 2.38)<br>1.62 (100 tablets tariff 2.38)<br>3.5 (100 tablets tariff 2.38)<br>2.78 (100 tablets tariff 2.38)<br>2.38 (100 tablets tariff 2.38)<br>4.5 (100 tablets tariff 2.38)<br>3.05 (100 tablets tariff 2.38)<br>2.5 (100 tablets tariff 2.38)<br>2.17 (100 tablets tariff 2.38)<br>2.2 (100 tablets tariff 2.38)<br>1.84 (100 tablets tariff 2.38)<br>2.53 (100 tablets tariff 2.38)<br>.6 (100 tablets tariff 2.38)<br>2.78 (100 tablets tariff 2.38)<br>3.25 (100 tablets tariff 2.38)<br>23.8 (1000 tablets)
na								0	
na								0	
na								0	
na								0	
na								0	
na								0	

ACS medicine	Ticagrelor 180mg<br>Aspirin 300mg<br>Fondaparinux (subcutaenous) GTN tablet	54.6 (90mg, tariff price 54.6)	0.23 (32 tablets, tariff 3.88)	62.79 (10 tablets, 2.5mg tariff 62.79)	1.89 (100, tariff cost 1.89)			119.51	medicine A: 180 not available, 90x2 is. No alternative costs.<br>alternative costs medicine B:<br>3.54 (28 tablets)<br>12.54 (100 tablets, tariff 12.11)<br>3.88 (32 tablets, tariff 3.88)<br>0.28 (32 tablets, tariff 3.88)<br>Medicin C: no alternative costs<br>medicine D alt costs:<br>1.97 (tariff price 1.89)<br>2.03 (tariff price 1.89)
na								0	
ACS medicines (ticagrelor, fondaparinux)<br>GTN<br>Paracetamol	Ticagrelor 180mg<br>Aspirin 300mg<br>Fondaparinux (subcutaenous) GTN tablet<br>paracetamol 1g	54.6 (90mg, tariff price 54.6)	0.23 (32 tablets, tariff 3.88)	62.79 (10 tablets, 2.5mg tariff 62.79)	1.89 (100, tariff cost 1.89)	.13 (16 tablets, 500mg)		119.64	medicine A: 180 not available, 90x2 is. No alternative costs.<br>alternative costs medicine B:<br>3.54 (28 tablets)<br>12.54 (100 tablets, tariff 12.11)<br>3.88 (32 tablets, tariff 3.88)<br>0.28 (32 tablets, tariff 3.88)<br>Medicin C: no alternative costs<br>medicine D alt costs:<br>1.97 (tariff price 1.89)<br>2.03 (tariff price 1.89)<br>Medicine E alt costs:<br>.93 (8 tablets)<br>.86 (12 tablets)<br>1.42 (14 tablets)<br>1.04 (16 tablets)<br>.76 (16 tablets)<br>.12 (16 tablets)<br>.27 (16 tablets)<br>.72 (16 tablets)<br>.18 (16 tablets)<br>.43 (16 tablets)<br>.26 (16 tablets)<br>.36 (16 tablets)<br>.34 (16 tablets)<br>.38 (16 tablets)<br>.17 (16 tablets)<br>.19 (16 tablets)<br>.31 (16 tablets)<br>.45 (16 tablets)<br>.67 (20 tablets)<br>.48 (20 tablets)<br>.54 (20 tablets)<br>.82 (30 tablets)<br>.15 (32 tablets tariff 0.76)<br>.62 (32 tablets tariff 0.76)<br>.2 (32 tablets tariff 0.76)<br>1.92 (32 tablets tariff 0.76)<br>.89 (32 tablets tariff 0.76)<br>.76 (32 tablets tariff 0.76)<br>1.44 (32 tablets tariff 0.76)<br>.33 (32 tablets tariff 0.76)<br>.68 (32 tablets tariff 0.76)<br>.52 (32 tablets tariff 0.76)<br>.49 (32 tablets tariff 0.76)<br>.29 (32 tablets tariff 0.76)<br>1.34 (100 tablets tariff 2.38)<br>1.95 (100 tablets tariff 2.38)<br>1.62 (100 tablets tariff 2.38)<br>3.5 (100 tablets tariff 2.38)<br>2.78 (100 tablets tariff 2.38)<br>2.38 (100 tablets tariff 2.38)<br>4.5 (100 tablets tariff 2.38)<br>3.05 (100 tablets tariff 2.38)<br>2.5 (100 tablets tariff 2.38)<br>2.17 (100 tablets tariff 2.38)

									2.2 (100 tablets tariff 2.38)<br>1.84 (100 tablets tariff 2.38)<br>2.53 (100 tablets tariff 2.38)<br>.6 (100 tablets tariff 2.38)<br>2.78 (100 tablets tariff 2.38)<br>3.25 (100 tablets tariff 2.38)<br>23.8 (1000 tablets)
Analgesia								0	
na								0	

Paracetamol<br>IV Co-Amoxiclav<br>N Saline	Oral Paracetamol<br>IV Co-Amoxiclav 1.3g<br>N Saline	.13 (16 tablets, 500mg)	10.60 (1000 + 200mg vial Amoxicillin)	2.62 Sodium chloride 0.9% (1l bottle, tariff 3.96)				13.35	Medicine A alt costs:<br>.93 (8 tablets)<br>.86 (12 tablets)<br>1.42 (14 tablets)<br>1.04 (16 tablets)<br>.76 (16 tablets)<br>.12 (16 tablets)<br>.27 (16 tablets)<br>.72 (16 tablets)<br>.18 (16 tablets)<br>.43 (16 tablets)<br>.26 (16 tablets)<br>.36 (16 tablets)<br>.34 (16 tablets)<br>.38 (16 tablets)<br>.17 (16 tablets)<br>.19 (16 tablets)<br>.31 (16 tablets)<br>.45 (16 tablets)<br>.67 (20 tablets)<br>.48 (20 tablets)<br>.54 (20 tablets)<br>.82 (30 tablets)<br>.15 (32 tablets tariff 0.76)<br>.62 (32 tablets tariff 0.76)<br>.2 (32 tablets tariff 0.76)<br>1.92 (32 tablets tariff 0.76)<br>.89 (32 tablets tariff 0.76)<br>.76 (32 tablets tariff 0.76)<br>1.44 (32 tablets tariff 0.76)<br>.33 (32 tablets tariff 0.76)<br>.68 (32 tablets tariff 0.76)<br>.52 (32 tablets tariff 0.76)<br>.49 (32 tablets tariff 0.76)<br>.29 (32 tablets tariff 0.76)<br>1.34 (100 tablets tariff 2.38)<br>1.95 (100 tablets tariff 2.38)<br>1.62 (100 tablets tariff 2.38)<br>3.5 (100 tablets tariff 2.38)<br>2.78 (100 tablets tariff 2.38)<br>2.38 (100 tablets tariff 2.38)<br>4.5 (100 tablets tariff 2.38)<br>3.05 (100 tablets tariff 2.38)<br>2.5 (100 tablets tariff 2.38)<br>2.17 (100 tablets tariff 2.38)<br>2.2 (100 tablets tariff 2.38)<br>1.84 (100 tablets tariff 2.38)<br>2.53 (100 tablets tariff 2.38)<br>.6 (100 tablets tariff 2.38)<br>2.78 (100 tablets tariff 2.38)<br>3.25 (100 tablets tariff 2.38)<br>23.8 (1000 tablets)<br>Medicine C alt cost:<br>4.12 1l bottle<br>24.72 6x 1lbottle<br>3.1 500ml bottle<br>37.2 12x500ml bottle<br>0.59 100ml bottle<br>11.78 20x 100ml bottle<br>2.62 1l bottle<br>26.2 10x 1l bottle<br>13.25 10x1l bottle<br>6.13 10x 250ml bottle<br>2.38 500ml bottle<br>23.80 10x 500ml bottle<br>13.25 500ml bottle<br>11.78 50ml bottle
na								0	

IV morphine (10mg x 2)<br>Candesartan	IV morphine 20mg oral candesartan	11.44 (10 ampoules, tariff cost: 11.45)	0.49 (4mg, 28 tablets)					11.93	Medicine A alternative costs:<br>11.45 (tariff cost: 11.45)<br>11.87 (tariff cost: 11.45)<br>14 (tariff cost: 11.45)<br>medicine B chose the most common + cheapest. Alt cost:<br>3.88 (7 tablets, tariff 0.66)<br>1.11 (7 tablets, tariff 0.66)<br>0.66(7 tablets, tariff 0.66)<br>3.1 (7 tablets, tariff 0.66)<br>9.78 (28 tablets)<br>3.08 (28 tablets)<br>2.64 (28 tablets)<br>7.82 (28 tablets)<br>3.44 (28 tablets)<br>9.29 (28 tablets)<br>8.31 (28 tablets)<br>22.65 (500 tablets)<br>61.43 (500 tablets)
na								0	
na								0	
N saline<br>IV Teicoplanin (400mg)<br>Ciprofloxacin (500mg)	N saline<br>IV Teicoplanin (400mg)<br>Ciprofloxacin (500mg)	2.62 Sodium chloride 0.9% (1l bottle, tariff 3.96)	7.32 (1 vial, tariff 7.32)	0.92 (10 tablets, tariff 1.57)				10.86	Medicine A alt cost:<br>4.12 1l bottle<br>24.72 6x 1lbottle<br>3.1 500ml bottle<br>37.2 12x500ml bottle<br>0.59 100ml bottle<br>11.78 20x 100ml bottle<br>2.62 1l bottle<br>26.2 10x 1l bottle<br>13.25 10x1l bottle<br>6.13 10x 250ml bottle<br>2.38 500ml bottle<br>23.80 10x 500ml bottle<br>13.25 500ml bottle<br>11.78 50ml bottle<br>Medicine B alt cost:<br>7.57 (1 vial, tariff 7.32)<br>medicine C alt cost:<br>2.19 (20 tablets, tariff 1.57)<br>2.18 (20 tablets, tariff 1.57)<br>10.62 (20 tablets, tariff 1.57)<br>1.05(20 tablets, tariff 1.57)<br>12.49(20 tablets, tariff 1.57)<br>27.29 (20 tablets)<br>1.84(20 tablets)<br>28.23(20 tablets)<br>3.14(20 tablets)<br>4.37(20 tablets)<br>4.9(20 tablets)<br>9.1 (100 tablets)

Tramadol (50mg)	Oral Tramadol (50mg)	28.51 (28 tablets, tariff 28.51)						28.51	Medicine A closest dosage is 400mg. alternative cost: 32.47 (30 tablets)
na								0	
Codeine (60mg)	Codeine phosphate oral 60mg, oral diclofenac 100mg, oral oramorph 5mg	1.71 200ml	7.58 (28 tablets, tariff 11.33)	3.29 (60 tablets morhpine sulphate, tariff 3.29)				12.58	medicine A alt costs:<br>1.90 (tariff: 1.90)<br>1.73 (tariff: 1.90)<br>16.80 for 2000ml<br>9.90 for 2000ml<br>medicine B alt costs:<br>8.20 (28 capsules, tariff 8.20)<br>11.33 (28 capsules, tariff 11.33)<br>Medicine C not available in 5mg, morphine sulphate same contents. No alt costs
na								0	
Ondansetron	Ondansetron 4mg IV	10.00 for 10 ampules (tarriff cost: 18.70)						10	Medicine A alt costs:<br>37.11 for 5 ampules (tarriff: 29.97)<br>18.70 for 10 ampules (tarrif cost: 18.70)<br>28.47 for 5 ampules (tarriff: 29.97)<br>5.40 for 5 ampules (tarrif: 29.97)<br>29.20 for 5 ampules (tarrif: 29.97)<br>5.80 for 5 ampules (tarrif cost: 29.97)<br>18.70 for 10 ampules (tarrif cost: 18.70).<br>decision: cheapest per ampule
na	?							0	
na								0	
na								0	
Peptac<br>Codeine	Peptac (sodium alginate with calcium carbonate and sodium bicarbonate) 10ml<br>Codeine phosphate 60mg	1.95 (500ml, tariff 1.95)	1.71 200ml					3.66	Medicine A alt costs: none.<br>medicine B alt costs:<br>1.90 (tariff: 1.90)<br>1.73 (tariff: 1.90)<br>16.80 for 2000ml<br>9.90 for 2000ml

Cyclizine	oral cyclzine 50mg, oral ondansetron 4mg	2.35 (30 tablets)	10.00 for 10 ampules (tarriff cost: 18.70)					12.35	Medicine A alt costs: 5.82 (100 tablets, 4.9 tariff)<br>5 (30 tablets)<br>Medicine B alt costs:<br>37.11 for 5 ampules (tarriff: 29.97)<br>18.70 for 10 ampules (tarrif cost: 18.70)<br>28.47 for 5 ampules (tarriff: 29.97)<br>5.40 for 5 ampules (tarrif: 29.97)<br>29.20 for 5 ampules (tarrif: 29.97)<br>5.80 for 5 ampules (tarrif cost: 29.97)<br>18.70 for 10 ampules (tarrif cost: 18.70).<br>decision: cheapest per ampule
na	IV ondansetron 4mg, IV normal saline	10.00 for 10 ampules (tarriff cost: 18.70)	2.62 Sodium chloride 0.9% (1l bottle, tariff 3.96)					12.62	Medicine A alt costs:<br>37.11 for 5 ampules (tarriff: 29.97)<br>18.70 for 10 ampules (tarrif cost: 18.70)<br>28.47 for 5 ampules (tarriff: 29.97)<br>5.40 for 5 ampules (tarrif: 29.97)<br>29.20 for 5 ampules (tarrif: 29.97)<br>5.80 for 5 ampules (tarrif cost: 29.97)<br>18.70 for 10 ampules (tarrif cost: 18.70).<br>decision: cheapest per ampule<br>Medicine B alt cost:<br>4.12 1l bottle<br>24.72 6x 1lbottle<br>3.1 500ml bottle<br>37.2 12x500ml bottle<br>0.59 100ml bottle<br>11.78 20x 100ml bottle<br>2.62 1l bottle<br>26.2 10x 1l bottle<br>13.25 10x1l bottle<br>6.13 10x 250ml bottle<br>2.38 500ml bottle<br>23.80 10x 500ml bottle<br>13.25 500ml bottle<br>11.78 50ml bottle
Peptac	Peptac (sodium alginate with calcium carbonate and sodium bicarbonate) 10ml	1.95 (500ml, tariff 1.95)						1.95	Medicine A alt costs: none
Naproxen	Narpoxen 50mg oral	45 (100ml, tariff 45.01)						45	medicine A alt cost:<br>45.01 (100ml, tariff 45.01)

Tazocin<br>Plasmalyte<br>Morphine<br>Cyclizine	Tazocin 4.5 g IV<br>Plasmalyte fluid IV<br>Morphine 10mg IV<br>Cyclizine 50 mg IV	12.90 (1 vial)	N/A	11.44 (10 ampoules, tariff cost: 11.45)	9.45 (5 ampoules, tariff 5.78			33.79	Medicine A alt costs:<br>15.75 (1 vial)<br>15.17(1 vial)<br>19.97 (1 vial)<br>36.50 (10 vials)<br>76.50 (10 vials)<br>48 (10 vials)<br>25 (10 vials)<br>99 (10 vials)<br>Medicine B no price information available at the moment<br>Medicine C alternative costs:<br>11.45 (tariff cost: 11.45)<br>11.87 (tariff cost: 11.45)<br>14 (tariff cost: 11.45)<br>Medicine D alt costs: 35 (10 amopoules)<br>16.25 (5 ampoules, 5.78 tariff)<br>32.50 (10 ampoules)<br>11.67 (5 ampoules, tariff 5.78)<br>12.74 (10 ampoules)<br>17.34 (10 ampoules)
Trimethoprim	Trimethoprim 200mg course	0.71 (6 tablets, tariff 0.71)						0.71	Medicine A alt costs:<br>0.91 (14 tablets, tariff 1.66)<br>3.8 (14 tablets, tariff 1.66)<br>5 (14 tablets, tariff 1.66)<br>1.04 (14 tablets, tariff 1.66)<br>2.71 (14 tablets, tariff 1.66)<br>6 (14 tablets, tariff 1.66)<br>3.61 (14 tablets, tariff 1.66)<br>2.9 (6 tablets, tariff 0.71)<br>2.15(6 tablets, tariff 0.71)
GTN	Ticagrelor 180mg<br>Aspirin 300mg<br>Morphine 10mg IV GTN tablet	54.6 (90mg, tariff price 54.6)	0.23 (32 tablets, tariff 3.88)	11.44 (10 ampoules, tariff cost: 11.45)	1.89 (100, tariff cost 1.89)			67.93	medicine A: 180 not available, 90x2 is. No alternative costs.<br>alternative costs medicine A:<br>3.54 (28 tablets)<br>12.54 (100 tablets, tariff 12.11)<br>3.88 (32 tablets, tariff 3.88)<br>0.28 (32 tablets, tariff 3.88)<br>Medicine C alternative costs:<br>11.45 (tariff cost: 11.45)<br>11.87 (tariff cost: 11.45)<br>14 (tariff cost: 11.45)medicine D alt costs:<br>1.97 (tariff price 1.89)<br>2.03 (tariff price 1.89)

Paracetamol	Paracetamol 1g (oral)	.13 (16 tablets, 500mg)						.13	Medicine A alt costs:<br>.93 (8 tablets)<br>.86 (12 tablets)<br>1.42 (14 tablets)<br>1.04 (16 tablets)<br>.76 (16 tablets)<br>.12 (16 tablets)<br>.27 (16 tablets)<br>.72 (16 tablets)<br>.18 (16 tablets)<br>.43 (16 tablets)<br>.26 (16 tablets)<br>.36 (16 tablets)<br>.34 (16 tablets)<br>.38 (16 tablets)<br>.17 (16 tablets)<br>.19 (16 tablets)<br>.31 (16 tablets)<br>.45 (16 tablets)<br>.67 (20 tablets)<br>.48 (20 tablets)<br>.54 (20 tablets)<br>.82 (30 tablets)<br>.15 (32 tablets tariff 0.76)<br>.62 (32 tablets tariff 0.76)<br>.2 (32 tablets tariff 0.76)<br>1.92 (32 tablets tariff 0.76)<br>.89 (32 tablets tariff 0.76)<br>.76 (32 tablets tariff 0.76)<br>1.44 (32 tablets tariff 0.76)<br>.33 (32 tablets tariff 0.76)<br>.68 (32 tablets tariff 0.76)<br>.52 (32 tablets tariff 0.76)<br>.49 (32 tablets tariff 0.76)<br>.29 (32 tablets tariff 0.76)<br>1.34 (100 tablets tariff 2.38)<br>1.95 (100 tablets tariff 2.38)<br>1.62 (100 tablets tariff 2.38)<br>3.5 (100 tablets tariff 2.38)<br>2.78 (100 tablets tariff 2.38)<br>2.38 (100 tablets tariff 2.38)<br>4.5 (100 tablets tariff 2.38)<br>3.05 (100 tablets tariff 2.38)<br>2.5 (100 tablets tariff 2.38)<br>2.17 (100 tablets tariff 2.38)<br>2.2 (100 tablets tariff 2.38)<br>1.84 (100 tablets tariff 2.38)<br>2.53 (100 tablets tariff 2.38)<br>.6 (100 tablets tariff 2.38)<br>2.78 (100 tablets tariff 2.38)<br>3.25 (100 tablets tariff 2.38)<br>23.8 (1000 tablets)
Aspirin	Aspirin oral 300mg	0.23 (32 tablets, tariff 3.88)						0.23	alternative costs medicine A:<br>3.54 (28 tablets)<br>12.54 (100 tablets, tariff 12.11)<br>3.88 (32 tablets, tariff 3.88)<br>0.28 (32 tablets, tariff 3.88)
Oramorph<br>Cyclizine	Oramorph oral<br>Cyclizine 50mg IV	1.89 (100ml oral sollution)	9.45 (5 ampoules, tariff 5.78					11.34	Medicine A alt costs:<br>5.45 (300ml tariff 4.42)<br>8.50 (500ml)<br>Medicine B alt costs: 35 (10 amopoules)<br>16.25 (5 ampoules, 5.78 tariff)<br>32.50 (10 ampoules)<br>11.67 (5 ampoules, tariff 5.78)<br>12.74 (10 ampoules)<br>17.34 (10 ampoules)

Aspirin	Aspirin oral 300mg	0.23 (32 tablets, tariff 3.88)						0.23	alternative costs medicine A:<br>3.54 (28 tablets)<br>12.54 (100 tablets, tariff 12.11)<br>3.88 (32 tablets, tariff 3.88)<br>0.28 (32 tablets, tariff 3.88)
na								0	
na								0	
Morphine<br>Cyclizine	Morphine 10mg IV<br>Ondansetron 4mg IV<br>Paracetamol 1g IV<br>Cyclizine 50mg IV	11.44 (10 ampoules, tariff cost: 11.45)	10.00 for 10 ampules (tarriff cost: 18.70)	14.40 (12 vials)	9.45 (5 ampoules, tariff 5.78			45.29	Medicine A alternative costs:<br>11.45 (tariff cost: 11.45)<br>11.87 (tariff cost: 11.45)<br>14 (tariff cost: 11.45)<br>Medicine B alt costs:<br>37.11 for 5 ampules (tarriff: 29.97)<br>18.70 for 10 ampules (tarrif cost: 18.70)<br>28.47 for 5 ampules (tarriff: 29.97)<br>5.40 for 5 ampules (tarrif: 29.97)<br>29.20 for 5 ampules (tarrif: 29.97)<br>5.80 for 5 ampules (tarrif cost: 29.97)<br>18.70 for 10 ampules (tarrif cost: 18.70).<br>decision: cheapest per ampule<br>Medicine C alt costs (NHS indicative):<br>14.40 for 12 vials<br>24 for 20 vials<br>17.90 for 10 vials (Tarriff 12)<br>Medicine D alt costs: 35 (10 amopoules)<br>16.25 (5 ampoules, 5.78 tariff)<br>32.50 (10 ampoules)<br>11.67 (5 ampoules, tariff 5.78)<br>12.74 (10 ampoules)<br>17.34 (10 ampoules)

Paracetamol (1g)<br>Codeine (400mg)	Paracetamol (1g) oral<br>Codeine phosphate<br>(400mg) oral	.13 (16 tablets, 500mg)	1.71 200ml					5.84	Medicine A alt costs:<br>.93 (8 tablets)<br>.86 (12 tablets)<br>1.42 (14 tablets)<br>1.04 (16 tablets)<br>.76 (16 tablets)<br>.12 (16 tablets)<br>.27 (16 tablets)<br>.72 (16 tablets)<br>.18 (16 tablets)<br>.43 (16 tablets)<br>.26 (16 tablets)<br>.36 (16 tablets)<br>.34 (16 tablets)<br>.38 (16 tablets)<br>.17 (16 tablets)<br>.19 (16 tablets)<br>.31 (16 tablets)<br>.45 (16 tablets)<br>.67 (20 tablets)<br>.48 (20 tablets)<br>.54 (20 tablets)<br>.82 (30 tablets)<br>.15 (32 tablets tariff 0.76)<br>.62 (32 tablets tariff 0.76)<br>.2 (32 tablets tariff 0.76)<br>1.92 (32 tablets tariff 0.76)<br>.89 (32 tablets tariff 0.76)<br>.76 (32 tablets tariff 0.76)<br>1.44 (32 tablets tariff 0.76)<br>.33 (32 tablets tariff 0.76)<br>.68 (32 tablets tariff 0.76)<br>.52 (32 tablets tariff 0.76)<br>.49 (32 tablets tariff 0.76)<br>.29 (32 tablets tariff 0.76)<br>1.34 (100 tablets tariff 2.38)<br>1.95 (100 tablets tariff 2.38)<br>1.62 (100 tablets tariff 2.38)<br>3.5 (100 tablets tariff 2.38)<br>2.78 (100 tablets tariff 2.38)<br>2.38 (100 tablets tariff 2.38)<br>4.5 (100 tablets tariff 2.38)<br>3.05 (100 tablets tariff 2.38)<br>2.5 (100 tablets tariff 2.38)<br>2.17 (100 tablets tariff 2.38)<br>2.2 (100 tablets tariff 2.38)<br>1.84 (100 tablets tariff 2.38)<br>2.53 (100 tablets tariff 2.38)<br>.6 (100 tablets tariff 2.38)<br>2.78 (100 tablets tariff 2.38)<br>3.25 (100 tablets tariff 2.38)<br>23.8 (1000 tablets)<br>medicine B alt costs:<br>1.90 (tariff: 1.90)<br>1.73 (tariff: 1.90)<br>16.80 for 2000ml<br>9.90 for 2000ml
na								0	

Aspirin (300g)<br>Ticagrelor (180mg)<br>N saline (500ml)<br>Actrapid (30 units in 50ml)<br>Fondaparinux (2.5mg)<br>Glycerol trinitrate (25mg in 50ml)	Aspirin (300g) oral<br>Ticagrelor (180mg) oral<br>N saline (500ml) fluids<br>Actrapid (30 units in 50ml) IV<br>Fondaparinux (2.5mg) subcutaneous<br>Glycerol trinitrate (25mg in 50ml) IV	0.23 (32 tablets, tariff 3.88)	54.6 (90mg, tariff price 54.6)	2.62 Sodium chloride 0.9% (1l bottle, tariff 3.96)	7.48 (100 units in 10ml, 1 vial, tariff 15.68)	62.79 (10 tablets, 2.5mg tariff 62.79)	32.45 (5 ampoules)	160.17	alternative costs medicine A:<br>3.54 (28 tablets)<br>12.54 (100 tablets, tariff 12.11)<br>3.88 (32 tablets, tariff 3.88)<br>0.28 (32 tablets, tariff 3.88)<br>medicine B: 180 not available, 90x2 is. No alternative costs.<br>Medicine C alt cost:<br>4.12 1l bottle<br>24.72 6x 1lbottle<br>3.1 500ml bottle<br>37.2 12x500ml bottle<br>0.59 100ml bottle<br>11.78 20x 100ml bottle<br>2.62 1l bottle<br>26.2 10x 1l bottle<br>13.25 10x1l bottle<br>6.13 10x 250ml bottle<br>2.38 500ml bottle<br>23.80 10x 500ml bottle<br>13.25 500ml bottle<br>11.78 50ml bottle<br>Medicine D dosage not available. 100units/10ml closest. alt costs:<br>15.68 (tariff 15.68)<br>19.08 (5 vials, tariff 19.08)<br>39.39 (tariff 39.39)<br>Medicin E: no alternative costs<br>medicine F: no alt costs
Paracetamol (1g)<br>N saline (1l)<br>Buscopan (20mg)	Paracetamol (1g) IV<br>N saline (1l) IV<br>Buscopan (20mg) IV	14.40 (12 vials)	2.62 Sodium chloride 0.9% (1l bottle, tariff 3.96)	2.92 (10 ampoules, tariff cost 2.92)				19.94	Medicine A alt costs (NHS indicative):<br>14.40 for 12 vials<br>24 for 20 vials<br>17.90 for 10 vials (Tarriff 12)<br>Medicine B alt cost:<br>4.12 1l bottle<br>24.72 6x 1lbottle<br>3.1 500ml bottle<br>37.2 12x500ml bottle<br>0.59 100ml bottle<br>11.78 20x 100ml bottle<br>2.62 1l bottle<br>26.2 10x 1l bottle<br>13.25 10x1l bottle<br>6.13 10x 250ml bottle<br>2.38 500ml bottle<br>23.80 10x 500ml bottle<br>13.25 500ml bottle<br>11.78 50ml bottle<br>Medicine C no alt costs

Ibuprofen (400mg)<br>Tramadol (500mg)	Ibuprofen oral (400mg)<br>Tramadol oral (500mg)	0.56 (24 tablets, tariff price 1.03)	28.51 (28 tablets, tariff 28.51)					29.07	Medicine X alt costs:<br>1.15 (48 tablets, tariff price 1.03)<br>2.05 (96 tablets, tariff price 1.03)<br>4.9 (60 tablets, 1.03 tariff)<br>2.06 (24 tablets, 1,03 tariff)<br>3.49 (48 tablets, 1.03 tariff)<br>5.49 (96 tablets, 1.03 tariff)<br>2.8 (84 tablets, 3.61 tariff)<br>1.39 (24 tablets, 1.03 tariff)<br>2.56 (48 tablets)<br>4.1 (84 tablets, 3.61 tariff)<br>1.03 (24 tablets, 1.03 tariff)<br>2.06 (48 tablets)<br>3.61 (84 tablets, 3.61 tariff)<br>6.14 (84 tablets, 3.61 tariff)<br>0.7 (24 tablets, 1.03 tariff)<br>0.85 (48 tablets)<br>1 (84 tablets, 3.61 tariff)<br>3 (96 tablets)<br>2.71, (84 tablets, 3.61 tariff)<br>5.74 (84 tablets, 3.61 tariff)<br>0.9 (24 tablets, 1.03 tariff)<br>3.01 (84 tablets, 3.61 tariff)<br>0.7 (24 tablets, 1.03 tariff)<br>1.17 (48 tablets)<br>2 (96 tablets)<br>Medicine B closest dosage is 400mg. alternative cost:<br>32.47 (30 tablets)
na								0	
na								0	
Morphine (1-10mg)	Morphine 10mg IV	11.44 (10 ampoules, tariff cost: 11.45)						11.44	Medicine A alt costs (NHS indicative):<br>14.40 for 12 vials<br>24 for 20 vials<br>17.90 for 10 vials (Tarriff 12)
na								0	

Diclofenac (100mg)<br>Paracetamol (1g)	Diclofenac oral (100mg)<br>Paracetamol oral (1g)	7.58 (28 tablets, tariff 11.33)	.13 (16 tablets, 500mg)					7.71	medicine A alt costs:<br>8.20 (28 capsules, tariff 8.20)<br>11.33 (28 capsules, tariff 11.33)<br>Medicine B alt costs:<br>.93 (8 tablets)<br>.86 (12 tablets)<br>1.42 (14 tablets)<br>1.04 (16 tablets)<br>.76 (16 tablets)<br>.12 (16 tablets)<br>.27 (16 tablets)<br>.72 (16 tablets)<br>.18 (16 tablets)<br>.43 (16 tablets)<br>.26 (16 tablets)<br>.36 (16 tablets)<br>.34 (16 tablets)<br>.38 (16 tablets)<br>.17 (16 tablets)<br>.19 (16 tablets)<br>.31 (16 tablets)<br>.45 (16 tablets)<br>.67 (20 tablets)<br>.48 (20 tablets)<br>.54 (20 tablets)<br>.82 (30 tablets)<br>.15 (32 tablets tariff 0.76)<br>.62 (32 tablets tariff 0.76)<br>.2 (32 tablets tariff 0.76)<br>1.92 (32 tablets tariff 0.76)<br>.89 (32 tablets tariff 0.76)<br>.76 (32 tablets tariff 0.76)<br>1.44 (32 tablets tariff 0.76)<br>.33 (32 tablets tariff 0.76)<br>.68 (32 tablets tariff 0.76)<br>.52 (32 tablets tariff 0.76)<br>.49 (32 tablets tariff 0.76)<br>.29 (32 tablets tariff 0.76)<br>1.34 (100 tablets tariff 2.38)<br>1.95 (100 tablets tariff 2.38)<br>1.62 (100 tablets tariff 2.38)<br>3.5 (100 tablets tariff 2.38)<br>2.78 (100 tablets tariff 2.38)<br>2.38 (100 tablets tariff 2.38)<br>4.5 (100 tablets tariff 2.38)<br>3.05 (100 tablets tariff 2.38)<br>2.5 (100 tablets tariff 2.38)<br>2.17 (100 tablets tariff 2.38)<br>2.2 (100 tablets tariff 2.38)<br>1.84 (100 tablets tariff 2.38)<br>2.53 (100 tablets tariff 2.38)<br>.6 (100 tablets tariff 2.38)<br>2.78 (100 tablets tariff 2.38)<br>3.25 (100 tablets tariff 2.38)<br>23.8 (1000 tablets)
Chlorphenamine (4mg)	Chlorphenamine oral (4mg)	0.45 (28 tablets, tariff 3.07)						0.45	Medicine A alt costs:<br>1.74 (28 tablets, tariff 3.07)<br>3.07 (28 tablets, tariff 3.07)<br>2.41 (28 tablets, tariff 3.07)<br>1 (28 tablets, tariff 3.07)<br>10.06 (500 tablets)<br>2.23 (30 tablets)<br>3.9 (60 tablets)

Ibuprofen (400mg)<br>Paracetamol (1g)	Ibuprofen oral(400mg)<br>Paracetamol oral (1g)	0.56 (24 tablets, tariff price 1.03)	.13 (16 tablets, 500mg)					.69	Medicine A alt costs:<br>1.15 (48 tablets, tariff price 1.03)<br>2.05 (96 tablets, tariff price 1.03)<br>4.9 (60 tablets, 1.03 tariff)<br>2.06 (24 tablets, 1,03 tariff)<br>3.49 (48 tablets, 1.03 tariff)<br>5.49 (96 tablets, 1.03 tariff)<br>2.8 (84 tablets, 3.61 tariff)<br>1.39 (24 tablets, 1.03 tariff)<br>2.56 (48 tablets)<br>4.1 (84 tablets, 3.61 tariff)<br>1.03 (24 tablets, 1.03 tariff)<br>2.06 (48 tablets)<br>3.61 (84 tablets, 3.61 tariff)<br>6.14 (84 tablets, 3.61 tariff)<br>0.7 (24 tablets, 1.03 tariff)<br>0.85 (48 tablets)<br>1 (84 tablets, 3.61 tariff)<br>3 (96 tablets)<br>2.71, (84 tablets, 3.61 tariff)<br>5.74 (84 tablets, 3.61 tariff)<br>0.9 (24 tablets, 1.03 tariff)<br>3.01 (84 tablets, 3.61 tariff)<br>0.7 (24 tablets, 1.03 tariff)<br>1.17 (48 tablets)<br>2 (96 tablets)<br>Medicine B alt costs:<br>.93 (8 tablets)<br>.86 (12 tablets)<br>1.42 (14 tablets)<br>1.04 (16 tablets)<br>.76 (16 tablets)<br>.12 (16 tablets)<br>.27 (16 tablets)<br>.72 (16 tablets)<br>.18 (16 tablets)<br>.43 (16 tablets)<br>.26 (16 tablets)<br>.36 (16 tablets)<br>.34 (16 tablets)<br>.38 (16 tablets)<br>.17 (16 tablets)<br>.19 (16 tablets)<br>.31 (16 tablets)<br>.45 (16 tablets)<br>.67 (20 tablets)<br>.48 (20 tablets)<br>.54 (20 tablets)<br>.82 (30 tablets)<br>.15 (32 tablets tariff 0.76)<br>.62 (32 tablets tariff 0.76)<br>.2 (32 tablets tariff 0.76)<br>1.92 (32 tablets tariff 0.76)<br>.89 (32 tablets tariff 0.76)<br>.76 (32 tablets tariff 0.76)<br>1.44 (32 tablets tariff 0.76)<br>.33 (32 tablets tariff 0.76)<br>.68 (32 tablets tariff 0.76)<br>.52 (32 tablets tariff 0.76)<br>.49 (32 tablets tariff 0.76)<br>.29 (32 tablets tariff 0.76)<br>1.34 (100 tablets tariff 2.38)<br>1.95 (100 tablets tariff 2.38)<br>1.62 (100 tablets tariff 2.38)<br>3.5 (100 tablets tariff 2.38)<br>2.78 (100 tablets tariff 2.38)<br>2.38 (100 tablets tariff 2.38)<br>4.5 (100 tablets tariff 2.38)<br>3.05 (100 tablets tariff 2.38)<br>2.5 (100 tablets tariff 2.38)<br>2.17 (100 tablets tariff 2.38)<br>2.2 (100 tablets tariff 2.38)<br>1.84 (100 tablets tariff 2.38)<br>2.53 (100 tablets tariff 2.38)<br>.6 (100 tablets tariff 2.38)<br>2.78 (100 tablets tariff 2.38)<br>3.25 (100 tablets tariff 2.38)<br>23.8 (1000 tablets)
na								0	

GTN (0.5mg)<br>Morphine (1-10mg)<br>Isosorbide dinitrate (25mg in 50ml 0.5ml/hr)<br>Oramorph (10mg)	GTN (0.5mg) oral<br>Morphine 10mg IV<br>Isosorbide dinitrate 25mg IV<br>Oramorph 10mg oral	1.89 (100, tariff cost 1.89)	11.44 (10 ampoules, tariff cost: 11.45)	67 (10 vials)	1.89 (100ml oral sollution)			82.22	medicine A alt costs:<br>1.97 (tariff price 1.89)<br>2.03 (tariff price 1.89)<br>Medicine B alternative costs:<br>11.45 (tariff cost: 11.45)<br>11.87 (tariff cost: 11.45)<br>14 (tariff cost: 11.45)<br>Medicine C no alt costs.<br>Medicine D alt costs:<br>5.45 (300ml tariff 4.42)<br>8.50 (500ml)
Cocodamol	Cocodamol (30/500)	11.99 (tariff cost: 11.99)						11.99	Medicine A alt costs: no oral options
na								0	
na								0	
Co-amoxiclav<br>Morphine<br>Paracetamol<br>Saline (0.9%)	Augmentin 1.2g IV<br>Morphine 10mg IV<br>Paracetamol 1g IV<br>Saline (0.9%)	10.60 (1000 + 200mg vial Amoxicillin)	11.44 (10 ampoules, tariff cost: 11.45)	14.40 (12 vials)	2.62 Sodium chloride 0.9% (1l bottle, tariff 3.96)			39.06	medicine A Closest dosage to 1.2g IV; 1000mg vial Amoxicillin + 200mg patassium clavulanate. Alt costs:<br>29.70 (1000+200mg)<br>10.96 (1000+200mg)<br>50 (1000+200mg)<br>27.50 (1000+200mg)<br>Medicine B alternative costs:<br>11.45 (tariff cost: 11.45)<br>11.87 (tariff cost: 11.45)<br>14 (tariff cost: 11.45)<br>Medicine C alt costs (NHS indicative):<br>14.40 for 12 vials<br>24 for 20 vials<br>17.90 for 10 vials (Tarriff 12)<br>Medicine D alt cost:<br>4.12 1l bottle<br>24.72 6x 1lbottle<br>3.1 500ml bottle<br>37.2 12x500ml bottle<br>0.59 100ml bottle<br>11.78 20x 100ml bottle<br>2.62 1l bottle<br>26.2 10x 1l bottle<br>13.25 10x1l bottle<br>6.13 10x 250ml bottle<br>2.38 500ml bottle<br>23.80 10x 500ml bottle<br>13.25 500ml bottle<br>11.78 50ml bottle
na								0	
Sulbutamol	Salbutamol nebuliser 5mg	5.87 (20 unit doses, tariff 5.97)						5.87	Medicine A alt costs:<br>5.97 (20 doses, tariff 5.97)<br>6.24 (20 doses, tariff 5.97)

Ondasetron<br>Saline<br>Paracetamol<br>Co-amoxiclav	Ondasetron 4mg IV<br>Normal Saline (fluids) IV<br>Paracetamol 1g<br>Augmentin 1.2g IV	10.00 for 10 ampules (tarriff cost: 18.70)	2.62 Sodium chloride 0.9% (1l bottle, tariff 3.96)	.13 (16 tablets, 500mg)	10.60 (1000 + 200mg vial Amoxicillin)			23.35	Medicine A alt costs:<br>37.11 for 5 ampules (tarriff: 29.97)<br>18.70 for 10 ampules (tarrif cost: 18.70)<br>28.47 for 5 ampules (tarriff: 29.97)<br>5.40 for 5 ampules (tarrif: 29.97)<br>29.20 for 5 ampules (tarrif: 29.97)<br>5.80 for 5 ampules (tarrif cost: 29.97)<br>18.70 for 10 ampules (tarrif cost: 18.70).<br>decision: cheapest per ampule<br>Medicine B alternative costs:<br>11.45 (tariff cost: 11.45)<br>11.87 (tariff cost: 11.45)<br>14 (tariff cost: 11.45)<br>Medicine A alt costs:<br>.93 (8 tablets)<br>.86 (12 tablets)<br>1.42 (14 tablets)<br>1.04 (16 tablets)<br>.76 (16 tablets)<br>.12 (16 tablets)<br>.27 (16 tablets)<br>.72 (16 tablets)<br>.18 (16 tablets)<br>.43 (16 tablets)<br>.26 (16 tablets)<br>.36 (16 tablets)<br>.34 (16 tablets)<br>.38 (16 tablets)<br>.17 (16 tablets)<br>.19 (16 tablets)<br>.31 (16 tablets)<br>.45 (16 tablets)<br>.67 (20 tablets)<br>.48 (20 tablets)<br>.54 (20 tablets)<br>.82 (30 tablets)<br>.15 (32 tablets tariff 0.76)<br>.62 (32 tablets tariff 0.76)<br>.2 (32 tablets tariff 0.76)<br>1.92 (32 tablets tariff 0.76)<br>.89 (32 tablets tariff 0.76)<br>.76 (32 tablets tariff 0.76)<br>1.44 (32 tablets tariff 0.76)<br>.33 (32 tablets tariff 0.76)<br>.68 (32 tablets tariff 0.76)<br>.52 (32 tablets tariff 0.76)<br>.49 (32 tablets tariff 0.76)<br>.29 (32 tablets tariff 0.76)<br>1.34 (100 tablets tariff 2.38)<br>1.95 (100 tablets tariff 2.38)<br>1.62 (100 tablets tariff 2.38)<br>3.5 (100 tablets tariff 2.38)<br>2.78 (100 tablets tariff 2.38)<br>2.38 (100 tablets tariff 2.38)<br>4.5 (100 tablets tariff 2.38)<br>3.05 (100 tablets tariff 2.38)<br>2.5 (100 tablets tariff 2.38)<br>2.17 (100 tablets tariff 2.38)<br>2.2 (100 tablets tariff 2.38)<br>1.84 (100 tablets tariff 2.38)<br>2.53 (100 tablets tariff 2.38)<br>.6 (100 tablets tariff 2.38)<br>2.78 (100 tablets tariff 2.38)<br>3.25 (100 tablets tariff 2.38)<br>23.8 (1000 tablets)<br>medicine D Closest dosage to 1.2g IV; 1000mg vial Amoxicillin + 200mg patassium clavulanate. Alt costs:<br>29.70 (1000+200mg)<br>10.96 (1000+200mg)

									50 (1000+200mg)<br>27.50 (1000+200mg)

Paracetamol	Paracetamol oral 1g	.13 (16 tablets, 500mg)						.13	Medicine A alt costs:<br>.93 (8 tablets)<br>.86 (12 tablets)<br>1.42 (14 tablets)<br>1.04 (16 tablets)<br>.76 (16 tablets)<br>.12 (16 tablets)<br>.27 (16 tablets)<br>.72 (16 tablets)<br>.18 (16 tablets)<br>.43 (16 tablets)<br>.26 (16 tablets)<br>.36 (16 tablets)<br>.34 (16 tablets)<br>.38 (16 tablets)<br>.17 (16 tablets)<br>.19 (16 tablets)<br>.31 (16 tablets)<br>.45 (16 tablets)<br>.67 (20 tablets)<br>.48 (20 tablets)<br>.54 (20 tablets)<br>.82 (30 tablets)<br>.15 (32 tablets tariff 0.76)<br>.62 (32 tablets tariff 0.76)<br>.2 (32 tablets tariff 0.76)<br>1.92 (32 tablets tariff 0.76)<br>.89 (32 tablets tariff 0.76)<br>.76 (32 tablets tariff 0.76)<br>1.44 (32 tablets tariff 0.76)<br>.33 (32 tablets tariff 0.76)<br>.68 (32 tablets tariff 0.76)<br>.52 (32 tablets tariff 0.76)<br>.49 (32 tablets tariff 0.76)<br>.29 (32 tablets tariff 0.76)<br>1.34 (100 tablets tariff 2.38)<br>1.95 (100 tablets tariff 2.38)<br>1.62 (100 tablets tariff 2.38)<br>3.5 (100 tablets tariff 2.38)<br>2.78 (100 tablets tariff 2.38)<br>2.38 (100 tablets tariff 2.38)<br>4.5 (100 tablets tariff 2.38)<br>3.05 (100 tablets tariff 2.38)<br>2.5 (100 tablets tariff 2.38)<br>2.17 (100 tablets tariff 2.38)<br>2.2 (100 tablets tariff 2.38)<br>1.84 (100 tablets tariff 2.38)<br>2.53 (100 tablets tariff 2.38)<br>.6 (100 tablets tariff 2.38)<br>2.78 (100 tablets tariff 2.38)<br>3.25 (100 tablets tariff 2.38)<br>23.8 (1000 tablets)
na								0	
na								0	
na								0	
na								0	
na	Cocodamol 30/500	11.99 (tariff cost: 11.99)						11.99	Medicine A alt costs: no oral options
na								0	

Co-amoxiclav<br>N saline<br>Paracetamol<br>Gentamycin	Augmentin 1.2g IV<br>N saline IV fluids<br>Paracetamol 1g IV<br>Gentamycin	10.60 (1000 + 200mg vial Amoxicillin)	2.62 Sodium chloride 0.9% (1l bottle, tariff 3.96)	14.40 (12 vials)	6.88 (5 Ampoules, tariff 6.88)			34.5	medicine A Closest dosage to 1.2g IV; 1000mg vial Amoxicillin + 200mg patassium clavulanate. Alt costs:<br>29.70 (1000+200mg)<br>10.96 (1000+200mg)<br>50 (1000+200mg)<br>27.50 (1000+200mg)<br>Medicine B alt cost:<br>4.12 1l bottle<br>24.72 6x 1lbottle<br>3.1 500ml bottle<br>37.2 12x500ml bottle<br>0.59 100ml bottle<br>11.78 20x 100ml bottle<br>2.62 1l bottle<br>26.2 10x 1l bottle<br>13.25 10x1l bottle<br>6.13 10x 250ml bottle<br>2.38 500ml bottle<br>23.80 10x 500ml bottle<br>13.25 500ml bottle<br>11.78 50ml bottle<br>Medicine C alt costs (NHS indicative):<br>14.40 for 12 vials<br>24 for 20 vials<br>17.90 for 10 vials (Tarriff 12)<br>Medicin D assumed to be the most common version, 80mg IV. alt costs: 12 (10 ampules)<br>13.76 (10 ampoules)<br>20 (5 ampoules, tariff 6.88)
IV morphine<br>Ondansetron	IV morphine 10mg<br>Normal Saline (fluids) IV<br>Ondansetron 4mg IV	11.44 (10 ampoules, tariff cost: 11.45)	2.62 Sodium chloride 0.9% (1l bottle, tariff 3.96)	10.00 for 10 ampules (tarriff cost: 18.70)				24.06	Medicine A alternative costs:<br>11.45 (tariff cost: 11.45)<br>11.87 (tariff cost: 11.45)<br>14 (tariff cost: 11.45)<br>Medicine C alt costs:<br>37.11 for 5 ampules (tarriff: 29.97)<br>18.70 for 10 ampules (tarrif cost: 18.70)<br>28.47 for 5 ampules (tarriff: 29.97)<br>5.40 for 5 ampules (tarrif: 29.97)<br>29.20 for 5 ampules (tarrif: 29.97)<br>5.80 for 5 ampules (tarrif cost: 29.97)<br>18.70 for 10 ampules (tarrif cost: 18.70).<br>decision: cheapest per ampule
na								0	
na								0	

Ticagrelor<br>Fondaparinux	Ticagrelor 180mg<br>Aspirin 300mg<br>Morphine 10mg IV	54.6 (90mg, tariff price 54.6)	0.23 (32 tablets, tariff 3.88)	11.44 (10 ampoules, tariff cost: 11.45)				66.04	medicine A: 180 not available, 90x2 is. No alternative costs.<br>alternative costs medicine A:<br>3.54 (28 tablets)<br>12.54 (100 tablets, tariff 12.11)<br>3.88 (32 tablets, tariff 3.88)<br>0.28 (32 tablets, tariff 3.88)<br>Medicine C alternative costs:<br>11.45 (tariff cost: 11.45)<br>11.87 (tariff cost: 11.45)<br>14 (tariff cost: 11.45)
na								0	
na								0	
IV fluids<br>IV antiemetic	IV Normal Saline (fluids)<br>IV ondansetron (4mg)	2.62 Sodium chloride 0.9% (1l bottle, tariff 3.96)	10.00 for 10 ampules (tarriff cost: 18.70)					21.32	Medicine A alt cost:<br>4.12 1l bottle<br>24.72 6x 1lbottle<br>3.1 500ml bottle<br>37.2 12x500ml bottle<br>0.59 100ml bottle<br>11.78 20x 100ml bottle<br>2.62 1l bottle<br>26.2 10x 1l bottle<br>13.25 10x1l bottle<br>6.13 10x 250ml bottle<br>2.38 500ml bottle<br>23.80 10x 500ml bottle<br>13.25 500ml bottle<br>11.78 50ml bottle<br>Medicine B alt costs:<br>37.11 for 5 ampules (tarriff: 29.97)<br>18.70 for 10 ampules (tarrif cost: 18.70)<br>28.47 for 5 ampules (tarriff: 29.97)<br>5.40 for 5 ampules (tarrif: 29.97)<br>29.20 for 5 ampules (tarrif: 29.97)<br>5.80 for 5 ampules (tarrif cost: 29.97)<br>18.70 for 10 ampules (tarrif cost: 18.70).<br>decision: cheapest per ampule
na								0	

Analgesia	Paracetamol 1g (oral)	.13 (16 tablets (500mg)						.13	Medicine A alt costs:<br>.93 (8 tablets)<br>.86 (12 tablets)<br>1.42 (14 tablets)<br>1.04 (16 tablets)<br>.76 (16 tablets)<br>.12 (16 tablets)<br>.27 (16 tablets)<br>.72 (16 tablets)<br>.18 (16 tablets)<br>.43 (16 tablets)<br>.26 (16 tablets)<br>.36 (16 tablets)<br>.34 (16 tablets)<br>.38 (16 tablets)<br>.17 (16 tablets)<br>.19 (16 tablets)<br>.31 (16 tablets)<br>.45 (16 tablets)<br>.67 (20 tablets)<br>.48 (20 tablets)<br>.54 (20 tablets)<br>.82 (30 tablets)<br>.15 (32 tablets tariff 0.76)<br>.62 (32 tablets tariff 0.76)<br>.2 (32 tablets tariff 0.76)<br>1.92 (32 tablets tariff 0.76)<br>.89 (32 tablets tariff 0.76)<br>.76 (32 tablets tariff 0.76)<br>1.44 (32 tablets tariff 0.76)<br>.33 (32 tablets tariff 0.76)<br>.68 (32 tablets tariff 0.76)<br>.52 (32 tablets tariff 0.76)<br>.49 (32 tablets tariff 0.76)<br>.29 (32 tablets tariff 0.76)<br>1.34 (100 tablets tariff 2.38)<br>1.95 (100 tablets tariff 2.38)<br>1.62 (100 tablets tariff 2.38)<br>3.5 (100 tablets tariff 2.38)<br>2.78 (100 tablets tariff 2.38)<br>2.38 (100 tablets tariff 2.38)<br>4.5 (100 tablets tariff 2.38)<br>3.05 (100 tablets tariff 2.38)<br>2.5 (100 tablets tariff 2.38)<br>2.17 (100 tablets tariff 2.38)<br>2.2 (100 tablets tariff 2.38)<br>1.84 (100 tablets tariff 2.38)<br>2.53 (100 tablets tariff 2.38)<br>.6 (100 tablets tariff 2.38)<br>2.78 (100 tablets tariff 2.38)<br>3.25 (100 tablets tariff 2.38)<br>23.8 (1000 tablets)
TTO co-dydramol	Dihydrocodeine with paracetamol oral course	2.49 (30 tablets, tariff 1.69)						2.49	10mg/500mg tablets most common Medicine A alt costs:<br>2.50 (30 tablets, tariff 1.69)<br>3.24(30 tablets, tariff 1.69)<br>4.50 (100 tablets, tariff 5.63)<br>7.59(100 tablets, tariff 5.63)<br>8.30(100 tablets, tariff 5.63)<br>9.75(100 tablets, tariff 5.63)<br>28.15 (500 tablets
na								0	
na								0	

Co-dydramol	Dihydrocodeine with paracetamol	2.49 (30 tablets, tariff 1.69)						2.49	10mg/500mg tablets most common Medicine A alt costs:<br>2.50 (30 tablets, tariff 1.69)<br>3.24(30 tablets, tariff 1.69)<br>4.50 (100 tablets, tariff 5.63)<br>7.59(100 tablets, tariff 5.63)<br>8.30(100 tablets, tariff 5.63)<br>9.75(100 tablets, tariff 5.63)<br>28.15 (500 tablets
Oral flucloxacillin (500mg QDS)	Oral flucloxacillin 500mg course	1.64 (28 capsules, tariff 1.87)						1.64	Medicine A alt cost:<br>2.19(28 capsules, tariff 1.87)<br>2.2(28 capsules, tariff 1.87)<br>2.81(28 capsules, tariff 1.87)<br>10.5(28 capsules, tariff 1.87)<br>9.93 (100 capsules)<br>6.68(100 capsules)<br>37.5 (100 capsules)
na								0	
na								0	
na								0	
IV fluids<br>IV ondansetron	IV Normal Saline (fluids)<br>IV ondansetron (4mg)	2.62 Sodium chloride 0.9% (1l bottle, tariff 3.96)	10.00 for 10 ampules (tarriff cost: 18.70)					12.62	Medicine A alt cost:<br>4.12 1l bottle<br>24.72 6x 1lbottle<br>3.1 500ml bottle<br>37.2 12x500ml bottle<br>0.59 100ml bottle<br>11.78 20x 100ml bottle<br>2.62 1l bottle<br>26.2 10x 1l bottle<br>13.25 10x1l bottle<br>6.13 10x 250ml bottle<br>2.38 500ml bottle<br>23.80 10x 500ml bottle<br>13.25 500ml bottle<br>11.78 50ml bottle<br>Medicine B alt costs:<br>37.11 for 5 ampules (tarriff: 29.97)<br>18.70 for 10 ampules (tarrif cost: 18.70)<br>28.47 for 5 ampules (tarriff: 29.97)<br>5.40 for 5 ampules (tarrif: 29.97)<br>29.20 for 5 ampules (tarrif: 29.97)<br>5.80 for 5 ampules (tarrif cost: 29.97)<br>18.70 for 10 ampules (tarrif cost: 18.70).<br>decision: cheapest per ampule
na								0	

TTO codydramol<br>TTO incentive spirometer	Codydramol course<br>TTO incentive spirometer (look like cost approx £12 depending on type)	2.49 (30 tablets, tariff 1.69)	18.99 (amazon)					21.48	10mg/500mg tablets most common Medicine A alt costs:<br>2.50 (30 tablets, tariff 1.69)<br>3.24(30 tablets, tariff 1.69)<br>4.50 (100 tablets, tariff 5.63)<br>7.59(100 tablets, tariff 5.63)<br>8.30(100 tablets, tariff 5.63)<br>9.75(100 tablets, tariff 5.63)<br>28.15 (500 tablets<br>medicine B alternative cost:<br>18.99 (amazon)<br>22.99 (medisave)<br>information from<br>https://www.medisave.co.uk/incentive-spirometer-triball.html?gclid=EAIaIQobChMIh-Xu8rPk9gIVj-3tCh3ZWAs-EAQYAyABEgL9kvD_BwE<br>and<br>https://www.amazon.co.uk/Ball-Breathing-exerciser-Incentive-Spirometer/dp/B00OLZ14KU
na								0	
na								0	
na								0	
na								0	
na								0	
na								0	
na								0	
na								0	
na								0	
na								0	
TTO naproxen<br>TTO trimethoprim	Narpoxen 50mg oral course<br>Trimethoprim 200mg oral course	45 (100ml, tariff 45.01)	0.71 (6 tablets, tariff 0.71)					45.71	medicine A alt cost:<br>45.01 (100ml, tariff 45.01)<br>Medicine B alt costs:<br>0.91 (14 tablets, tariff 1.66)<br>3.8 (14 tablets, tariff 1.66)<br>5 (14 tablets, tariff 1.66)<br>1.04 (14 tablets, tariff 1.66)<br>2.71 (14 tablets, tariff 1.66)<br>6 (14 tablets, tariff 1.66)<br>3.61 (14 tablets, tariff 1.66)<br>2.9 (6 tablets, tariff 0.71)<br>2.15(6 tablets, tariff 0.71)
na								0	

Unknown IV medication<br>Unknown oral medication<br>TTO Lansoprazole	Lansoprazole IV<br>Course of lansoprazole 30mg oral	3.55 (28 tablets, tariff: 4.13)	3.55 (28 tablets, tariff: 4.13)					7.1	No IV option available for Lansoprazole.<br>medicine A & B alt costs:<br>1.90 (tariff: 1.90)<br>1.73 (tariff: 1.90)<br>16.80 for 2000ml<br>9.90 for 2000ml
TTO oramorph	Oramorph oral course	1.89 (100ml oral sollution)						1.89	Medicine D alt costs:<br>5.45 (300ml tariff 4.42)<br>8.50 (500ml)
TTO cefalexin	Oral cefalexin 500mg course	2.25 (21 tablets, tariff 2.26						2.25	Medicine A alt cost:<br>2.26 (21 tablets, tariff 2.26)<br>2.59 (21 tablets, tariff 2.26)<br>2.38 (21 tablets, tariff 2.26)<br>5.35 (21 tablets, tariff 2.26)
na								0	
na								0	
Paracetamol	Paracetamol 1g (oral)	.13 (16 tablets 500mg)-						.13	Medicine A alt costs:<br>.93 (8 tablets)<br>.86 (12 tablets)<br>1.42 (14 tablets)<br>1.04 (16 tablets)<br>.76 (16 tablets)<br>.12 (16 tablets)<br>.27 (16 tablets)<br>.72 (16 tablets)<br>.18 (16 tablets)<br>.43 (16 tablets)<br>.26 (16 tablets)<br>.36 (16 tablets)<br>.34 (16 tablets)<br>.38 (16 tablets)<br>.17 (16 tablets)<br>.19 (16 tablets)<br>.31 (16 tablets)<br>.45 (16 tablets)<br>.67 (20 tablets)<br>.48 (20 tablets)<br>.54 (20 tablets)<br>.82 (30 tablets)<br>.15 (32 tablets tariff 0.76)<br>.62 (32 tablets tariff 0.76)<br>.2 (32 tablets tariff 0.76)<br>1.92 (32 tablets tariff 0.76)<br>.89 (32 tablets tariff 0.76)<br>.76 (32 tablets tariff 0.76)<br>1.44 (32 tablets tariff 0.76)<br>.33 (32 tablets tariff 0.76)<br>.68 (32 tablets tariff 0.76)<br>.52 (32 tablets tariff 0.76)<br>.49 (32 tablets tariff 0.76)<br>.29 (32 tablets tariff 0.76)<br>1.34 (100 tablets tariff 2.38)<br>1.95 (100 tablets tariff 2.38)<br>1.62 (100 tablets tariff 2.38)<br>3.5 (100 tablets tariff 2.38)<br>2.78 (100 tablets tariff 2.38)<br>2.38 (100 tablets tariff 2.38)<br>4.5 (100 tablets tariff 2.38)<br>3.05 (100 tablets tariff 2.38)<br>2.5 (100 tablets tariff 2.38)<br>2.17 (100 tablets tariff 2.38)<br>2.2 (100 tablets tariff 2.38)<br>1.84 (100 tablets tariff 2.38)<br>2.53 (100 tablets tariff 2.38)<br>.6 (100 tablets tariff 2.38)<br>2.78 (100 tablets tariff 2.38)<br>3.25 (100 tablets tariff 2.38)<br>23.8 (1000 tablets)

na								0	
na								0	
TTO antibiotics	Amoxicillin 500mg oral course	0.41 (15 capsules, tariff 0.91)						0.41	Medicin A alt costs:<br>1.8 (15 capsules, tariff 0.91)<br>2.03 (21 capsules, tariff 1.27)<br>6.73 (100 capsules)<br>2.50 (21 capsules, tariff 1.27)<br>1.49 (15 capsules, tariff 0.91)<br>1.88 (21 capsules, tariff 1.27)<br>7.95 (100 capsules)<br>10.99 (21 capsules, tariff 1.27)<br>0.45 (21 capsules, tariff 1.27)<br>7.5 (15 capsules, tariff 0.91)<br>15 (21 capsules, tariff 1.27)<br>75 (100 capsules)<br>0.73 (21 capsules, tariff 1.27)<br>0.78 (21 capsules, tariff 1.27)
na								0	
na								0	
na								0	
na								0	
TTO Trimethoprim	Trimethoprim 200mg oral course	0.71 (6 tablets, tariff 0.71)						0.71	Medicine A alt costs:<br>0.91 (14 tablets, tariff 1.66)<br>3.8 (14 tablets, tariff 1.66)<br>5 (14 tablets, tariff 1.66)<br>1.04 (14 tablets, tariff 1.66)<br>2.71 (14 tablets, tariff 1.66)<br>6 (14 tablets, tariff 1.66)<br>3.61 (14 tablets, tariff 1.66)<br>2.9 (6 tablets, tariff 0.71)<br>2.15(6 tablets, tariff 0.71)
na								0	
na								0	
Aspirin (300mg)	Aspirin oral (300mg)	0.23 (32 tablets, tariff 3.88)						0.23	alternative costs medicine A:<br>3.54 (28 tablets)<br>12.54 (100 tablets, tariff 12.11)<br>3.88 (32 tablets, tariff 3.88)<br>0.28 (32 tablets, tariff 3.88)
na	Diclofenac 100mg PR	3.04 (10 suppository, tariff 3.64)						3.04	Medicine A alt cost:<br>3.64 (10suppository, tariff 3.64

Hartmanns (500mg)<br>Hyoscine	Hartmans (fluid) IV<br>Hyoscine 20mg oral<br>Ondansetron 4mg IV<br>Cyclzine 50mg IV	3.95	10.71 (100 tablets)	10.00 for 10 ampules (tarriff cost: 18.70)	9.45 (5 ampoule, tariff 5.78)			34.11	Medicine A no info available, used: https://www.medekit.com/drugs/intravenous-fluids/hartmanns-solution-500ml-500-mlt-6587/<br>Medicine B no alternative costs.<br>Medicine C alt costs:<br>37.11 for 5 ampules (tarriff: 29.97)<br>18.70 for 10 ampules (tarrif cost: 18.70)<br>28.47 for 5 ampules (tarriff: 29.97)<br>5.40 for 5 ampules (tarrif: 29.97)<br>29.20 for 5 ampules (tarrif: 29.97)<br>5.80 for 5 ampules (tarrif cost: 29.97)<br>18.70 for 10 ampules (tarrif cost: 18.70).<br>decision: cheapest per ampule<br>medicine D alt cost:<br>16.25 (5 ampoule, tariff: 5.78)<br>11.67 (5 ampoule, tariff: 5.78)<br>35 (10 ampoule)<br>32.50 (10 ampoule)<br>12.74 (10 ampoule)<br>17.34 (10 ampoule)

Rectal diclofenac	Diclofenac 100mg PR<br>Codein phosphate 60mg oral<br>Paracetamol 1 oral	3.04 (10 suppository, tariff 3.64)	1.71 200ml	.13 (16 tablets 500mg)				4.88	Medicine A alt cost:<br>3.64 (10suppository, tariff 3.64<br>medicine B alt costs:<br>1.90 (tariff: 1.90)<br>1.73 (tariff: 1.90)<br>16.80 for 2000ml<br>9.90 for 2000ml<br>Medicine C alt costs:<br>.93 (8 tablets)<br>.86 (12 tablets)<br>1.42 (14 tablets)<br>1.04 (16 tablets)<br>.76 (16 tablets)<br>.12 (16 tablets)<br>.27 (16 tablets)<br>.72 (16 tablets)<br>.18 (16 tablets)<br>.43 (16 tablets)<br>.26 (16 tablets)<br>.36 (16 tablets)<br>.34 (16 tablets)<br>.38 (16 tablets)<br>.17 (16 tablets)<br>.19 (16 tablets)<br>.31 (16 tablets)<br>.45 (16 tablets)<br>.67 (20 tablets)<br>.48 (20 tablets)<br>.54 (20 tablets)<br>.82 (30 tablets)<br>.15 (32 tablets tariff 0.76)<br>.62 (32 tablets tariff 0.76)<br>.2 (32 tablets tariff 0.76)<br>1.92 (32 tablets tariff 0.76)<br>.89 (32 tablets tariff 0.76)<br>.76 (32 tablets tariff 0.76)<br>1.44 (32 tablets tariff 0.76)<br>.33 (32 tablets tariff 0.76)<br>.68 (32 tablets tariff 0.76)<br>.52 (32 tablets tariff 0.76)<br>.49 (32 tablets tariff 0.76)<br>.29 (32 tablets tariff 0.76)<br>1.34 (100 tablets tariff 2.38)<br>1.95 (100 tablets tariff 2.38)<br>1.62 (100 tablets tariff 2.38)<br>3.5 (100 tablets tariff 2.38)<br>2.78 (100 tablets tariff 2.38)<br>2.38 (100 tablets tariff 2.38)<br>4.5 (100 tablets tariff 2.38)<br>3.05 (100 tablets tariff 2.38)<br>2.5 (100 tablets tariff 2.38)<br>2.17 (100 tablets tariff 2.38)<br>2.2 (100 tablets tariff 2.38)<br>1.84 (100 tablets tariff 2.38)<br>2.53 (100 tablets tariff 2.38)<br>.6 (100 tablets tariff 2.38)<br>2.78 (100 tablets tariff 2.38)<br>3.25 (100 tablets tariff 2.38)<br>23.8 (1000 tablets)
na	GTN tablet<br>Aspirin 300mg oral	1.89 (100, tariff cost 1.89)	0.23 (32 tablets, tariff 3.88)					2.12	medicine A alt costs:<br>1.97 (tariff price 1.89)<br>2.03 (tariff price 1.89)<br>alternative costs medicine A:<br>3.54 (28 tablets)<br>12.54 (100 tablets, tariff 12.11)<br>3.88 (32 tablets, tariff 3.88)<br>0.28 (32 tablets, tariff 3.88)

Anticoagulant	Dalteparin (subcutaneous)	18.58 (2.5k units/0.2ml, 10 syringes. Tariff 18.58						18.58	Medicine A alt Costs:<br>28.32 (10k units/1ml, 5 syringes. Tariff 28.23)<br>35.29 (12.5k units/0.5ml, 5 syringes. Tariff 35.29)<br>51.22 (10k units/0.5ml, 10 syringes. Tariff 51.22)<br>51.22 (10k units/4ml, 10 syringes. Tariff 51.22)<br>48.66 (100k units/4ml, 1 syringes. Tariff 48.66)<br>42.34 (15k units/0.6ml, 10 syringes. Tariff 42.34)<br>50.82 (18k units/0.72ml, 5 syringes. Tariff 50.82)<br>28.23 (5k units/0.2ml, 10 syringes. Tariff 28.23)<br>42.34 (7.5k units/0.3ml, 10 syringes. Tariff 42.34)
na								0	
na								0	
na								0	
na								0	
na								0	
na								0	
TTO antibiotics	Nitrofurantoin 50mg course	4.20 (28 tablets, tariff 4.20)						4.20	Medicine A alt costs:<br>7.51 (28 tablets, tariff 4.20)<br>31.33 (28 tablets, tariff 4.20)<br>5.08 (28 tablets, tariff 4.20)<br>7.64 (28 tablets, tariff 4.20)<br>26.31 (100 tablets)<br>18.14(100 tablets)<br>111.89 (100 tablets)

Cyclizine (50mg)<br>Paracetamol (1g)	Cyclizine oral (50mg)<br>Paracetamol oral (1g)	2.35 (30 tablets)	.13 (16 tablets 500mg)					2.48	Medicine A alt costs: 5.82 (100 tablets, 4.9 tariff)<br>5 (30 tablets)<br>Medicine B alt costs:<br>.93 (8 tablets)<br>.86 (12 tablets)<br>1.42 (14 tablets)<br>1.04 (16 tablets)<br>.76 (16 tablets)<br>.12 (16 tablets)<br>.27 (16 tablets)<br>.72 (16 tablets)<br>.18 (16 tablets)<br>.43 (16 tablets)<br>.26 (16 tablets)<br>.36 (16 tablets)<br>.34 (16 tablets)<br>.38 (16 tablets)<br>.17 (16 tablets)<br>.19 (16 tablets)<br>.31 (16 tablets)<br>.45 (16 tablets)<br>.67 (20 tablets)<br>.48 (20 tablets)<br>.54 (20 tablets)<br>.82 (30 tablets)<br>.15 (32 tablets tariff 0.76)<br>.62 (32 tablets tariff 0.76)<br>.2 (32 tablets tariff 0.76)<br>1.92 (32 tablets tariff 0.76)<br>.89 (32 tablets tariff 0.76)<br>.76 (32 tablets tariff 0.76)<br>1.44 (32 tablets tariff 0.76)<br>.33 (32 tablets tariff 0.76)<br>.68 (32 tablets tariff 0.76)<br>.52 (32 tablets tariff 0.76)<br>.49 (32 tablets tariff 0.76)<br>.29 (32 tablets tariff 0.76)<br>1.34 (100 tablets tariff 2.38)<br>1.95 (100 tablets tariff 2.38)<br>1.62 (100 tablets tariff 2.38)<br>3.5 (100 tablets tariff 2.38)<br>2.78 (100 tablets tariff 2.38)<br>2.38 (100 tablets tariff 2.38)<br>4.5 (100 tablets tariff 2.38)<br>3.05 (100 tablets tariff 2.38)<br>2.5 (100 tablets tariff 2.38)<br>2.17 (100 tablets tariff 2.38)<br>2.2 (100 tablets tariff 2.38)<br>1.84 (100 tablets tariff 2.38)<br>2.53 (100 tablets tariff 2.38)<br>.6 (100 tablets tariff 2.38)<br>2.78 (100 tablets tariff 2.38)<br>3.25 (100 tablets tariff 2.38)<br>23.8 (1000 tablets)
na								0	

na	Paracetamol 1g (oral)	.13 (16 tablets 500mg)						.13	Medicine A alt costs:<br>.93 (8 tablets)<br>.86 (12 tablets)<br>1.42 (14 tablets)<br>1.04 (16 tablets)<br>.76 (16 tablets)<br>.12 (16 tablets)<br>.27 (16 tablets)<br>.72 (16 tablets)<br>.18 (16 tablets)<br>.43 (16 tablets)<br>.26 (16 tablets)<br>.36 (16 tablets)<br>.34 (16 tablets)<br>.38 (16 tablets)<br>.17 (16 tablets)<br>.19 (16 tablets)<br>.31 (16 tablets)<br>.45 (16 tablets)<br>.67 (20 tablets)<br>.48 (20 tablets)<br>.54 (20 tablets)<br>.82 (30 tablets)<br>.15 (32 tablets tariff 0.76)<br>.62 (32 tablets tariff 0.76)<br>.2 (32 tablets tariff 0.76)<br>1.92 (32 tablets tariff 0.76)<br>.89 (32 tablets tariff 0.76)<br>.76 (32 tablets tariff 0.76)<br>1.44 (32 tablets tariff 0.76)<br>.33 (32 tablets tariff 0.76)<br>.68 (32 tablets tariff 0.76)<br>.52 (32 tablets tariff 0.76)<br>.49 (32 tablets tariff 0.76)<br>.29 (32 tablets tariff 0.76)<br>1.34 (100 tablets tariff 2.38)<br>1.95 (100 tablets tariff 2.38)<br>1.62 (100 tablets tariff 2.38)<br>3.5 (100 tablets tariff 2.38)<br>2.78 (100 tablets tariff 2.38)<br>2.38 (100 tablets tariff 2.38)<br>4.5 (100 tablets tariff 2.38)<br>3.05 (100 tablets tariff 2.38)<br>2.5 (100 tablets tariff 2.38)<br>2.17 (100 tablets tariff 2.38)<br>2.2 (100 tablets tariff 2.38)<br>1.84 (100 tablets tariff 2.38)<br>2.53 (100 tablets tariff 2.38)<br>.6 (100 tablets tariff 2.38)<br>2.78 (100 tablets tariff 2.38)<br>3.25 (100 tablets tariff 2.38)<br>23.8 (1000 tablets)
na								0	

Paracetamol	Paracetamol 1g (oral)	.13 (16 tablets)						.13	Medicine A alt costs:<br>.93 (8 tablets)<br>.86 (12 tablets)<br>1.42 (14 tablets)<br>1.04 (16 tablets)<br>.76 (16 tablets)<br>.12 (16 tablets)<br>.27 (16 tablets)<br>.72 (16 tablets)<br>.18 (16 tablets)<br>.43 (16 tablets)<br>.26 (16 tablets)<br>.36 (16 tablets)<br>.34 (16 tablets)<br>.38 (16 tablets)<br>.17 (16 tablets)<br>.19 (16 tablets)<br>.31 (16 tablets)<br>.45 (16 tablets)<br>.67 (20 tablets)<br>.48 (20 tablets)<br>.54 (20 tablets)<br>.82 (30 tablets)<br>.15 (32 tablets tariff 0.76)<br>.62 (32 tablets tariff 0.76)<br>.2 (32 tablets tariff 0.76)<br>1.92 (32 tablets tariff 0.76)<br>.89 (32 tablets tariff 0.76)<br>.76 (32 tablets tariff 0.76)<br>1.44 (32 tablets tariff 0.76)<br>.33 (32 tablets tariff 0.76)<br>.68 (32 tablets tariff 0.76)<br>.52 (32 tablets tariff 0.76)<br>.49 (32 tablets tariff 0.76)<br>.29 (32 tablets tariff 0.76)<br>1.34 (100 tablets tariff 2.38)<br>1.95 (100 tablets tariff 2.38)<br>1.62 (100 tablets tariff 2.38)<br>3.5 (100 tablets tariff 2.38)<br>2.78 (100 tablets tariff 2.38)<br>2.38 (100 tablets tariff 2.38)<br>4.5 (100 tablets tariff 2.38)<br>3.05 (100 tablets tariff 2.38)<br>2.5 (100 tablets tariff 2.38)<br>2.17 (100 tablets tariff 2.38)<br>2.2 (100 tablets tariff 2.38)<br>1.84 (100 tablets tariff 2.38)<br>2.53 (100 tablets tariff 2.38)<br>.6 (100 tablets tariff 2.38)<br>2.78 (100 tablets tariff 2.38)<br>3.25 (100 tablets tariff 2.38)<br>23.8 (1000 tablets)
Plasmalyte	Plasmalyte (fluids) IV	N/A						0	Medicine Adoes not seem to be currently available
Co-amoxiclav	Augmentin 1.2g IV	10.60 (1000 + 200mg vial Amoxicillin)						0	medicine A Closest dosage to 1.2g IV; 1000mg vial Amoxicillin + 200mg patassium clavulanate. Alt costs:<br>29.70 (1000+200mg)<br>10.96 (1000+200mg)<br>50 (1000+200mg)<br>27.50 (1000+200mg)

Paracetamol<br>Codeine<br>GTN	Paracetamol 1g oral<br>Codeine phosphate 60mg oral<br>GTN tablet	.13 (16 tablets, 500mg)	1.71 200ml	1.89 (100, tariff cost 1.89)				3.72	Medicine A alt costs:<br>.93 (8 tablets)<br>.86 (12 tablets)<br>1.42 (14 tablets)<br>1.04 (16 tablets)<br>.76 (16 tablets)<br>.12 (16 tablets)<br>.27 (16 tablets)<br>.72 (16 tablets)<br>.18 (16 tablets)<br>.43 (16 tablets)<br>.26 (16 tablets)<br>.36 (16 tablets)<br>.34 (16 tablets)<br>.38 (16 tablets)<br>.17 (16 tablets)<br>.19 (16 tablets)<br>.31 (16 tablets)<br>.45 (16 tablets)<br>.67 (20 tablets)<br>.48 (20 tablets)<br>.54 (20 tablets)<br>.82 (30 tablets)<br>.15 (32 tablets tariff 0.76)<br>.62 (32 tablets tariff 0.76)<br>.2 (32 tablets tariff 0.76)<br>1.92 (32 tablets tariff 0.76)<br>.89 (32 tablets tariff 0.76)<br>.76 (32 tablets tariff 0.76)<br>1.44 (32 tablets tariff 0.76)<br>.33 (32 tablets tariff 0.76)<br>.68 (32 tablets tariff 0.76)<br>.52 (32 tablets tariff 0.76)<br>.49 (32 tablets tariff 0.76)<br>.29 (32 tablets tariff 0.76)<br>1.34 (100 tablets tariff 2.38)<br>1.95 (100 tablets tariff 2.38)<br>1.62 (100 tablets tariff 2.38)<br>3.5 (100 tablets tariff 2.38)<br>2.78 (100 tablets tariff 2.38)<br>2.38 (100 tablets tariff 2.38)<br>4.5 (100 tablets tariff 2.38)<br>3.05 (100 tablets tariff 2.38)<br>2.5 (100 tablets tariff 2.38)<br>2.17 (100 tablets tariff 2.38)<br>2.2 (100 tablets tariff 2.38)<br>1.84 (100 tablets tariff 2.38)<br>2.53 (100 tablets tariff 2.38)<br>.6 (100 tablets tariff 2.38)<br>2.78 (100 tablets tariff 2.38)<br>3.25 (100 tablets tariff 2.38)<br>23.8 (1000 tablets)<br>medicine B alt costs:<br>1.90 (tariff: 1.90)<br>1.73 (tariff: 1.90)<br>16.80 for 2000ml<br>9.90 for 2000ml<br>medicine C alt costs:<br>1.97 (tariff price 1.89)<br>2.03 (tariff price 1.89)

Paracetamol	Paracetamol 1g (oral)	.13 (16tablets 500mg)						.13	Medicine A alt costs:<br>.93 (8 tablets)<br>.86 (12 tablets)<br>1.42 (14 tablets)<br>1.04 (16 tablets)<br>.76 (16 tablets)<br>.12 (16 tablets)<br>.27 (16 tablets)<br>.72 (16 tablets)<br>.18 (16 tablets)<br>.43 (16 tablets)<br>.26 (16 tablets)<br>.36 (16 tablets)<br>.34 (16 tablets)<br>.38 (16 tablets)<br>.17 (16 tablets)<br>.19 (16 tablets)<br>.31 (16 tablets)<br>.45 (16 tablets)<br>.67 (20 tablets)<br>.48 (20 tablets)<br>.54 (20 tablets)<br>.82 (30 tablets)<br>.15 (32 tablets tariff 0.76)<br>.62 (32 tablets tariff 0.76)<br>.2 (32 tablets tariff 0.76)<br>1.92 (32 tablets tariff 0.76)<br>.89 (32 tablets tariff 0.76)<br>.76 (32 tablets tariff 0.76)<br>1.44 (32 tablets tariff 0.76)<br>.33 (32 tablets tariff 0.76)<br>.68 (32 tablets tariff 0.76)<br>.52 (32 tablets tariff 0.76)<br>.49 (32 tablets tariff 0.76)<br>.29 (32 tablets tariff 0.76)<br>1.34 (100 tablets tariff 2.38)<br>1.95 (100 tablets tariff 2.38)<br>1.62 (100 tablets tariff 2.38)<br>3.5 (100 tablets tariff 2.38)<br>2.78 (100 tablets tariff 2.38)<br>2.38 (100 tablets tariff 2.38)<br>4.5 (100 tablets tariff 2.38)<br>3.05 (100 tablets tariff 2.38)<br>2.5 (100 tablets tariff 2.38)<br>2.17 (100 tablets tariff 2.38)<br>2.2 (100 tablets tariff 2.38)<br>1.84 (100 tablets tariff 2.38)<br>2.53 (100 tablets tariff 2.38)<br>.6 (100 tablets tariff 2.38)<br>2.78 (100 tablets tariff 2.38)<br>3.25 (100 tablets tariff 2.38)<br>23.8 (1000 tablets)
na								0	

Paracetamol	Paracetamol 1g (oral)	.13 (16 tablets 500mg)						.13	Medicine A alt costs:<br>.93 (8 tablets)<br>.86 (12 tablets)<br>1.42 (14 tablets)<br>1.04 (16 tablets)<br>.76 (16 tablets)<br>.12 (16 tablets)<br>.27 (16 tablets)<br>.72 (16 tablets)<br>.18 (16 tablets)<br>.43 (16 tablets)<br>.26 (16 tablets)<br>.36 (16 tablets)<br>.34 (16 tablets)<br>.38 (16 tablets)<br>.17 (16 tablets)<br>.19 (16 tablets)<br>.31 (16 tablets)<br>.45 (16 tablets)<br>.67 (20 tablets)<br>.48 (20 tablets)<br>.54 (20 tablets)<br>.82 (30 tablets)<br>.15 (32 tablets tariff 0.76)<br>.62 (32 tablets tariff 0.76)<br>.2 (32 tablets tariff 0.76)<br>1.92 (32 tablets tariff 0.76)<br>.89 (32 tablets tariff 0.76)<br>.76 (32 tablets tariff 0.76)<br>1.44 (32 tablets tariff 0.76)<br>.33 (32 tablets tariff 0.76)<br>.68 (32 tablets tariff 0.76)<br>.52 (32 tablets tariff 0.76)<br>.49 (32 tablets tariff 0.76)<br>.29 (32 tablets tariff 0.76)<br>1.34 (100 tablets tariff 2.38)<br>1.95 (100 tablets tariff 2.38)<br>1.62 (100 tablets tariff 2.38)<br>3.5 (100 tablets tariff 2.38)<br>2.78 (100 tablets tariff 2.38)<br>2.38 (100 tablets tariff 2.38)<br>4.5 (100 tablets tariff 2.38)<br>3.05 (100 tablets tariff 2.38)<br>2.5 (100 tablets tariff 2.38)<br>2.17 (100 tablets tariff 2.38)<br>2.2 (100 tablets tariff 2.38)<br>1.84 (100 tablets tariff 2.38)<br>2.53 (100 tablets tariff 2.38)<br>.6 (100 tablets tariff 2.38)<br>2.78 (100 tablets tariff 2.38)<br>3.25 (100 tablets tariff 2.38)<br>23.8 (1000 tablets)

Co-amoxiclav<br>Salbutamol<br>Prednisolone<br>Ipratropium<br>Plasmalyte	Augmentin 1.2g IV<br>Salbutamol 5mg nebuliser<br>Prednisolone 40mg oral<br>Ipratropium 0.5mg nebuliser<br>Plasmalyte IV infusion	10.60 (1000 + 200mg vial Amoxicillin)	5.87 (20 unit doses, tariff 5.97)	19.45 (28tablets of 20mg, tariff: 19.45)	2.73 (20 doses, tariff: 2.73)	N/A		38.65	medicine A Closest dosage to 1.2g IV; 1000mg vial Amoxicillin + 200mg patassium clavulanate. Alt costs:<br>29.70 (1000+200mg)<br>10.96 (1000+200mg)<br>50 (1000+200mg)<br>27.50 (1000+200mg)<br>Medicine B alt costs:<br>5.97 (20 doses, tariff 5.97)<br>6.24 (20 doses, tariff 5.97)medicine C Closest dosage to 40mg available. alt costs: none.<br>5.97 (20 doses, tariff 5.97)<br>6.24 (20 doses, tariff 5.97)<br>medicine D alt cost;<br>4.87 (20 doses, tariff 2.73)<br>15.99 (20 doses, tariff 2.73)<br>3.58 (20 doses, tariff 2.73)<br>3.04 (20 doses, tariff 2.73)<br>No prices currently available for medicine E

Paracetamol<br>Codeine<br>Peptac	Paracetamol 1g oral<br>Codeine phosphate 60mg oral<br>Sodium alginate with calcium carbonate and sodium bicarbonate	.13 (16 tablets 500mg)	1.71 200ml	1.95 (500ml, tariff 1.95)				2.08	Medicine A alt costs:<br>.93 (8 tablets)<br>.86 (12 tablets)<br>1.42 (14 tablets)<br>1.04 (16 tablets)<br>.76 (16 tablets)<br>.12 (16 tablets)<br>.27 (16 tablets)<br>.72 (16 tablets)<br>.18 (16 tablets)<br>.43 (16 tablets)<br>.26 (16 tablets)<br>.36 (16 tablets)<br>.34 (16 tablets)<br>.38 (16 tablets)<br>.17 (16 tablets)<br>.19 (16 tablets)<br>.31 (16 tablets)<br>.45 (16 tablets)<br>.67 (20 tablets)<br>.48 (20 tablets)<br>.54 (20 tablets)<br>.82 (30 tablets)<br>.15 (32 tablets tariff 0.76)<br>.62 (32 tablets tariff 0.76)<br>.2 (32 tablets tariff 0.76)<br>1.92 (32 tablets tariff 0.76)<br>.89 (32 tablets tariff 0.76)<br>.76 (32 tablets tariff 0.76)<br>1.44 (32 tablets tariff 0.76)<br>.33 (32 tablets tariff 0.76)<br>.68 (32 tablets tariff 0.76)<br>.52 (32 tablets tariff 0.76)<br>.49 (32 tablets tariff 0.76)<br>.29 (32 tablets tariff 0.76)<br>1.34 (100 tablets tariff 2.38)<br>1.95 (100 tablets tariff 2.38)<br>1.62 (100 tablets tariff 2.38)<br>3.5 (100 tablets tariff 2.38)<br>2.78 (100 tablets tariff 2.38)<br>2.38 (100 tablets tariff 2.38)<br>4.5 (100 tablets tariff 2.38)<br>3.05 (100 tablets tariff 2.38)<br>2.5 (100 tablets tariff 2.38)<br>2.17 (100 tablets tariff 2.38)<br>2.2 (100 tablets tariff 2.38)<br>1.84 (100 tablets tariff 2.38)<br>2.53 (100 tablets tariff 2.38)<br>.6 (100 tablets tariff 2.38)<br>2.78 (100 tablets tariff 2.38)<br>3.25 (100 tablets tariff 2.38)<br>23.8 (1000 tablets)<br>medicine B alt costs:<br>1.90 (tariff: 1.90)<br>1.73 (tariff: 1.90)<br>16.80 for 2000ml<br>9.90 for 2000ml<br>Medicine C alt costs: none

N saline	IV fluids (N/saline)	2.62 Sodium chloride 0.9% (1l bottle, tariff 3.96)						2.62	Medicine A alt cost:<br>4.12 1l bottle<br>24.72 6x 1lbottle<br>3.1 500ml bottle<br>37.2 12x500ml bottle<br>0.59 100ml bottle<br>11.78 20x 100ml bottle<br>2.62 1l bottle<br>26.2 10x 1l bottle<br>13.25 10x1l bottle<br>6.13 10x 250ml bottle<br>2.38 500ml bottle<br>23.80 10x 500ml bottle<br>13.25 500ml bottle<br>11.78 50ml bottle
na								0	
na								0	
na								0	
na								0	
GTN<br>Aspirin	GTN tablet<br>Aspirin 300mg oral	1.89 (100, tariff cost 1.89)	0.23 (32 tablets, tariff 3.88)					2.12	medicine A alt costs:<br>1.97 (tariff price 1.89)<br>2.03 (tariff price 1.89)<br>alternative costs medicine A:<br>3.54 (28 tablets)<br>12.54 (100 tablets, tariff 12.11)<br>3.88 (32 tablets, tariff 3.88)<br>0.28 (32 tablets, tariff 3.88)
na								0	
na								0	
na								0	
na								0	
na								0	
na								0	
na	Cyclizine oral (50mg)<br>Diclofenac 100mg PR	2.35 (30 tablets)	3.04 (10 suppository, tariff 3.64)					5.39	Medicine A alt costs: 5.82 (100 tablets, 4.9 tariff)<br>5 (30 tablets)<br>Medicine B alt cost:<br>3.64 (10suppository, tariff 3.64
Codeine (30mg)	Cyclizine oral (50mg)	2.35 (30 tablets)						2.35	Medicine A alt costs: 5.82 (100 tablets, 4.9 tariff)<br>5 (30 tablets)
na								0	

Paracetamol	Paracetamol 1g oral	.13 (16 tablets 500mg)						.13	Medicine A alt costs:<br>.93 (8 tablets)<br>.86 (12 tablets)<br>1.42 (14 tablets)<br>1.04 (16 tablets)<br>.76 (16 tablets)<br>.12 (16 tablets)<br>.27 (16 tablets)<br>.72 (16 tablets)<br>.18 (16 tablets)<br>.43 (16 tablets)<br>.26 (16 tablets)<br>.36 (16 tablets)<br>.34 (16 tablets)<br>.38 (16 tablets)<br>.17 (16 tablets)<br>.19 (16 tablets)<br>.31 (16 tablets)<br>.45 (16 tablets)<br>.67 (20 tablets)<br>.48 (20 tablets)<br>.54 (20 tablets)<br>.82 (30 tablets)<br>.15 (32 tablets tariff 0.76)<br>.62 (32 tablets tariff 0.76)<br>.2 (32 tablets tariff 0.76)<br>1.92 (32 tablets tariff 0.76)<br>.89 (32 tablets tariff 0.76)<br>.76 (32 tablets tariff 0.76)<br>1.44 (32 tablets tariff 0.76)<br>.33 (32 tablets tariff 0.76)<br>.68 (32 tablets tariff 0.76)<br>.52 (32 tablets tariff 0.76)<br>.49 (32 tablets tariff 0.76)<br>.29 (32 tablets tariff 0.76)<br>1.34 (100 tablets tariff 2.38)<br>1.95 (100 tablets tariff 2.38)<br>1.62 (100 tablets tariff 2.38)<br>3.5 (100 tablets tariff 2.38)<br>2.78 (100 tablets tariff 2.38)<br>2.38 (100 tablets tariff 2.38)<br>4.5 (100 tablets tariff 2.38)<br>3.05 (100 tablets tariff 2.38)<br>2.5 (100 tablets tariff 2.38)<br>2.17 (100 tablets tariff 2.38)<br>2.2 (100 tablets tariff 2.38)<br>1.84 (100 tablets tariff 2.38)<br>2.53 (100 tablets tariff 2.38)<br>.6 (100 tablets tariff 2.38)<br>2.78 (100 tablets tariff 2.38)<br>3.25 (100 tablets tariff 2.38)<br>23.8 (1000 tablets)
na								0	
na								0	

Paracetamol	Paracetamol 1g oral	.13 (16 tablets 500mg)						.13	Medicine A alt costs:<br>.93 (8 tablets)<br>.86 (12 tablets)<br>1.42 (14 tablets)<br>1.04 (16 tablets)<br>.76 (16 tablets)<br>.12 (16 tablets)<br>.27 (16 tablets)<br>.72 (16 tablets)<br>.18 (16 tablets)<br>.43 (16 tablets)<br>.26 (16 tablets)<br>.36 (16 tablets)<br>.34 (16 tablets)<br>.38 (16 tablets)<br>.17 (16 tablets)<br>.19 (16 tablets)<br>.31 (16 tablets)<br>.45 (16 tablets)<br>.67 (20 tablets)<br>.48 (20 tablets)<br>.54 (20 tablets)<br>.82 (30 tablets)<br>.15 (32 tablets tariff 0.76)<br>.62 (32 tablets tariff 0.76)<br>.2 (32 tablets tariff 0.76)<br>1.92 (32 tablets tariff 0.76)<br>.89 (32 tablets tariff 0.76)<br>.76 (32 tablets tariff 0.76)<br>1.44 (32 tablets tariff 0.76)<br>.33 (32 tablets tariff 0.76)<br>.68 (32 tablets tariff 0.76)<br>.52 (32 tablets tariff 0.76)<br>.49 (32 tablets tariff 0.76)<br>.29 (32 tablets tariff 0.76)<br>1.34 (100 tablets tariff 2.38)<br>1.95 (100 tablets tariff 2.38)<br>1.62 (100 tablets tariff 2.38)<br>3.5 (100 tablets tariff 2.38)<br>2.78 (100 tablets tariff 2.38)<br>2.38 (100 tablets tariff 2.38)<br>4.5 (100 tablets tariff 2.38)<br>3.05 (100 tablets tariff 2.38)<br>2.5 (100 tablets tariff 2.38)<br>2.17 (100 tablets tariff 2.38)<br>2.2 (100 tablets tariff 2.38)<br>1.84 (100 tablets tariff 2.38)<br>2.53 (100 tablets tariff 2.38)<br>.6 (100 tablets tariff 2.38)<br>2.78 (100 tablets tariff 2.38)<br>3.25 (100 tablets tariff 2.38)<br>23.8 (1000 tablets)
na								0	
na	Trimethoprim 200mg course	0.71 (6 tablets, tariff 0.71)						.71	Medicine A alt costs:<br>0.91 (14 tablets, tariff 1.66)<br>3.8 (14 tablets, tariff 1.66)<br>5 (14 tablets, tariff 1.66)<br>1.04 (14 tablets, tariff 1.66)<br>2.71 (14 tablets, tariff 1.66)<br>6 (14 tablets, tariff 1.66)<br>3.61 (14 tablets, tariff 1.66)<br>2.9 (6 tablets, tariff 0.71)<br>2.15(6 tablets, tariff 0.71)

TTO Trimethoprim	Trimethoprim 200mg course	0.71 (6 tablets, tariff 0.71)						0.71	Medicine A alt costs:<br>0.91 (14 tablets, tariff 1.66)<br>3.8 (14 tablets, tariff 1.66)<br>5 (14 tablets, tariff 1.66)<br>1.04 (14 tablets, tariff 1.66)<br>2.71 (14 tablets, tariff 1.66)<br>6 (14 tablets, tariff 1.66)<br>3.61 (14 tablets, tariff 1.66)<br>2.9 (6 tablets, tariff 0.71)<br>2.15(6 tablets, tariff 0.71)
na								0	
Paracetamol	Paracetamol 1g oral	.13 (16 tablets 500mg)						.13	Medicine A alt costs:<br>.93 (8 tablets)<br>.86 (12 tablets)<br>1.42 (14 tablets)<br>1.04 (16 tablets)<br>.76 (16 tablets)<br>.12 (16 tablets)<br>.27 (16 tablets)<br>.72 (16 tablets)<br>.18 (16 tablets)<br>.43 (16 tablets)<br>.26 (16 tablets)<br>.36 (16 tablets)<br>.34 (16 tablets)<br>.38 (16 tablets)<br>.17 (16 tablets)<br>.19 (16 tablets)<br>.31 (16 tablets)<br>.45 (16 tablets)<br>.67 (20 tablets)<br>.48 (20 tablets)<br>.54 (20 tablets)<br>.82 (30 tablets)<br>.15 (32 tablets tariff 0.76)<br>.62 (32 tablets tariff 0.76)<br>.2 (32 tablets tariff 0.76)<br>1.92 (32 tablets tariff 0.76)<br>.89 (32 tablets tariff 0.76)<br>.76 (32 tablets tariff 0.76)<br>1.44 (32 tablets tariff 0.76)<br>.33 (32 tablets tariff 0.76)<br>.68 (32 tablets tariff 0.76)<br>.52 (32 tablets tariff 0.76)<br>.49 (32 tablets tariff 0.76)<br>.29 (32 tablets tariff 0.76)<br>1.34 (100 tablets tariff 2.38)<br>1.95 (100 tablets tariff 2.38)<br>1.62 (100 tablets tariff 2.38)<br>3.5 (100 tablets tariff 2.38)<br>2.78 (100 tablets tariff 2.38)<br>2.38 (100 tablets tariff 2.38)<br>4.5 (100 tablets tariff 2.38)<br>3.05 (100 tablets tariff 2.38)<br>2.5 (100 tablets tariff 2.38)<br>2.17 (100 tablets tariff 2.38)<br>2.2 (100 tablets tariff 2.38)<br>1.84 (100 tablets tariff 2.38)<br>2.53 (100 tablets tariff 2.38)<br>.6 (100 tablets tariff 2.38)<br>2.78 (100 tablets tariff 2.38)<br>3.25 (100 tablets tariff 2.38)<br>23.8 (1000 tablets)

Paracetamol<br>Codeine	Paracetamol 1g oral<br>Codeine phosphate 30mg oral	.13 (16 tablets 500mg)	1.71 200ml					1.84	Medicine A alt costs:<br>.93 (8 tablets)<br>.86 (12 tablets)<br>1.42 (14 tablets)<br>1.04 (16 tablets)<br>.76 (16 tablets)<br>.12 (16 tablets)<br>.27 (16 tablets)<br>.72 (16 tablets)<br>.18 (16 tablets)<br>.43 (16 tablets)<br>.26 (16 tablets)<br>.36 (16 tablets)<br>.34 (16 tablets)<br>.38 (16 tablets)<br>.17 (16 tablets)<br>.19 (16 tablets)<br>.31 (16 tablets)<br>.45 (16 tablets)<br>.67 (20 tablets)<br>.48 (20 tablets)<br>.54 (20 tablets)<br>.82 (30 tablets)<br>.15 (32 tablets tariff 0.76)<br>.62 (32 tablets tariff 0.76)<br>.2 (32 tablets tariff 0.76)<br>1.92 (32 tablets tariff 0.76)<br>.89 (32 tablets tariff 0.76)<br>.76 (32 tablets tariff 0.76)<br>1.44 (32 tablets tariff 0.76)<br>.33 (32 tablets tariff 0.76)<br>.68 (32 tablets tariff 0.76)<br>.52 (32 tablets tariff 0.76)<br>.49 (32 tablets tariff 0.76)<br>.29 (32 tablets tariff 0.76)<br>1.34 (100 tablets tariff 2.38)<br>1.95 (100 tablets tariff 2.38)<br>1.62 (100 tablets tariff 2.38)<br>3.5 (100 tablets tariff 2.38)<br>2.78 (100 tablets tariff 2.38)<br>2.38 (100 tablets tariff 2.38)<br>4.5 (100 tablets tariff 2.38)<br>3.05 (100 tablets tariff 2.38)<br>2.5 (100 tablets tariff 2.38)<br>2.17 (100 tablets tariff 2.38)<br>2.2 (100 tablets tariff 2.38)<br>1.84 (100 tablets tariff 2.38)<br>2.53 (100 tablets tariff 2.38)<br>.6 (100 tablets tariff 2.38)<br>2.78 (100 tablets tariff 2.38)<br>3.25 (100 tablets tariff 2.38)<br>23.8 (1000 tablets)<br>medicine B alt costs:<br>1.90 (tariff: 1.90)<br>1.73 (tariff: 1.90)<br>16.80 for 2000ml<br>9.90 for 2000ml
na								0	

na	Paracetamol 1g oral	.13 (16 tablets 500mg)						.13	Medicine A alt costs:<br>.93 (8 tablets)<br>.86 (12 tablets)<br>1.42 (14 tablets)<br>1.04 (16 tablets)<br>.76 (16 tablets)<br>.12 (16 tablets)<br>.27 (16 tablets)<br>.72 (16 tablets)<br>.18 (16 tablets)<br>.43 (16 tablets)<br>.26 (16 tablets)<br>.36 (16 tablets)<br>.34 (16 tablets)<br>.38 (16 tablets)<br>.17 (16 tablets)<br>.19 (16 tablets)<br>.31 (16 tablets)<br>.45 (16 tablets)<br>.67 (20 tablets)<br>.48 (20 tablets)<br>.54 (20 tablets)<br>.82 (30 tablets)<br>.15 (32 tablets tariff 0.76)<br>.62 (32 tablets tariff 0.76)<br>.2 (32 tablets tariff 0.76)<br>1.92 (32 tablets tariff 0.76)<br>.89 (32 tablets tariff 0.76)<br>.76 (32 tablets tariff 0.76)<br>1.44 (32 tablets tariff 0.76)<br>.33 (32 tablets tariff 0.76)<br>.68 (32 tablets tariff 0.76)<br>.52 (32 tablets tariff 0.76)<br>.49 (32 tablets tariff 0.76)<br>.29 (32 tablets tariff 0.76)<br>1.34 (100 tablets tariff 2.38)<br>1.95 (100 tablets tariff 2.38)<br>1.62 (100 tablets tariff 2.38)<br>3.5 (100 tablets tariff 2.38)<br>2.78 (100 tablets tariff 2.38)<br>2.38 (100 tablets tariff 2.38)<br>4.5 (100 tablets tariff 2.38)<br>3.05 (100 tablets tariff 2.38)<br>2.5 (100 tablets tariff 2.38)<br>2.17 (100 tablets tariff 2.38)<br>2.2 (100 tablets tariff 2.38)<br>1.84 (100 tablets tariff 2.38)<br>2.53 (100 tablets tariff 2.38)<br>.6 (100 tablets tariff 2.38)<br>2.78 (100 tablets tariff 2.38)<br>3.25 (100 tablets tariff 2.38)<br>23.8 (1000 tablets)

Paracetamol<br>Codeine	Paracetamol 1g oral<br>Codeine phosphate 30mg oral	.13 (16 tablets, 500mg)	1.71 200ml					1.84	Medicine A alt costs:<br>.93 (8 tablets)<br>.86 (12 tablets)<br>1.42 (14 tablets)<br>1.04 (16 tablets)<br>.76 (16 tablets)<br>.12 (16 tablets)<br>.27 (16 tablets)<br>.72 (16 tablets)<br>.18 (16 tablets)<br>.43 (16 tablets)<br>.26 (16 tablets)<br>.36 (16 tablets)<br>.34 (16 tablets)<br>.38 (16 tablets)<br>.17 (16 tablets)<br>.19 (16 tablets)<br>.31 (16 tablets)<br>.45 (16 tablets)<br>.67 (20 tablets)<br>.48 (20 tablets)<br>.54 (20 tablets)<br>.82 (30 tablets)<br>.15 (32 tablets tariff 0.76)<br>.62 (32 tablets tariff 0.76)<br>.2 (32 tablets tariff 0.76)<br>1.92 (32 tablets tariff 0.76)<br>.89 (32 tablets tariff 0.76)<br>.76 (32 tablets tariff 0.76)<br>1.44 (32 tablets tariff 0.76)<br>.33 (32 tablets tariff 0.76)<br>.68 (32 tablets tariff 0.76)<br>.52 (32 tablets tariff 0.76)<br>.49 (32 tablets tariff 0.76)<br>.29 (32 tablets tariff 0.76)<br>1.34 (100 tablets tariff 2.38)<br>1.95 (100 tablets tariff 2.38)<br>1.62 (100 tablets tariff 2.38)<br>3.5 (100 tablets tariff 2.38)<br>2.78 (100 tablets tariff 2.38)<br>2.38 (100 tablets tariff 2.38)<br>4.5 (100 tablets tariff 2.38)<br>3.05 (100 tablets tariff 2.38)<br>2.5 (100 tablets tariff 2.38)<br>2.17 (100 tablets tariff 2.38)<br>2.2 (100 tablets tariff 2.38)<br>1.84 (100 tablets tariff 2.38)<br>2.53 (100 tablets tariff 2.38)<br>.6 (100 tablets tariff 2.38)<br>2.78 (100 tablets tariff 2.38)<br>3.25 (100 tablets tariff 2.38)<br>23.8 (1000 tablets)<br>medicine B alt costs:<br>1.90 (tariff: 1.90)<br>1.73 (tariff: 1.90)<br>16.80 for 2000ml<br>9.90 for 2000ml
na								0	
na								0	
Trimethoprim	Trimethoprim 200mg	0.71 (6 tablets, tariff 0.71)						0.71	Medicine A alt costs:<br>0.91 (14 tablets, tariff 1.66)<br>3.8 (14 tablets, tariff 1.66)<br>5 (14 tablets, tariff 1.66)<br>1.04 (14 tablets, tariff 1.66)<br>2.71 (14 tablets, tariff 1.66)<br>6 (14 tablets, tariff 1.66)<br>3.61 (14 tablets, tariff 1.66)<br>2.9 (6 tablets, tariff 0.71)<br>2.15(6 tablets, tariff 0.71)

TTO Codeine (30mg)	Codeine phosphate oral<br>60mg course	1.71 200ml						1.71	medicine A alt costs:<br>1.90 (tariff: 1.90)<br>1.73 (tariff: 1.90)<br>16.80 for 2000ml<br>9.90 for 2000ml
Paracetamol (1g)<br>TTO Codeine (30mg)	Paracetamol (1g) oral<br>Codeine phosphate oral<br>30mg course	.13 (16 tablets 500mg)	1.71 200ml					1.84	Medicine A alt costs:<br>.93 (8 tablets)<br>.86 (12 tablets)<br>1.42 (14 tablets)<br>1.04 (16 tablets)<br>.76 (16 tablets)<br>.12 (16 tablets)<br>.27 (16 tablets)<br>.72 (16 tablets)<br>.18 (16 tablets)<br>.43 (16 tablets)<br>.26 (16 tablets)<br>.36 (16 tablets)<br>.34 (16 tablets)<br>.38 (16 tablets)<br>.17 (16 tablets)<br>.19 (16 tablets)<br>.31 (16 tablets)<br>.45 (16 tablets)<br>.67 (20 tablets)<br>.48 (20 tablets)<br>.54 (20 tablets)<br>.82 (30 tablets)<br>.15 (32 tablets tariff 0.76)<br>.62 (32 tablets tariff 0.76)<br>.2 (32 tablets tariff 0.76)<br>1.92 (32 tablets tariff 0.76)<br>.89 (32 tablets tariff 0.76)<br>.76 (32 tablets tariff 0.76)<br>1.44 (32 tablets tariff 0.76)<br>.33 (32 tablets tariff 0.76)<br>.68 (32 tablets tariff 0.76)<br>.52 (32 tablets tariff 0.76)<br>.49 (32 tablets tariff 0.76)<br>.29 (32 tablets tariff 0.76)<br>1.34 (100 tablets tariff 2.38)<br>1.95 (100 tablets tariff 2.38)<br>1.62 (100 tablets tariff 2.38)<br>3.5 (100 tablets tariff 2.38)<br>2.78 (100 tablets tariff 2.38)<br>2.38 (100 tablets tariff 2.38)<br>4.5 (100 tablets tariff 2.38)<br>3.05 (100 tablets tariff 2.38)<br>2.5 (100 tablets tariff 2.38)<br>2.17 (100 tablets tariff 2.38)<br>2.2 (100 tablets tariff 2.38)<br>1.84 (100 tablets tariff 2.38)<br>2.53 (100 tablets tariff 2.38)<br>.6 (100 tablets tariff 2.38)<br>2.78 (100 tablets tariff 2.38)<br>3.25 (100 tablets tariff 2.38)<br>23.8 (1000 tablets)<br>medicine B alt costs:<br>1.90 (tariff: 1.90)<br>1.73 (tariff: 1.90)<br>16.80 for 2000ml<br>9.90 for 2000ml
na								0	
na								0	
na								0	
na								0	
na								0	
na								0	
na								0	
na								0	

TTO Nitrofurantoin	Nitrofurantoin 50mg course	4.20 (28 tablets, tariff 4.20)						4.20	Medicine A alt costs:<br>7.51 (28 tablets, tariff 4.20)<br>31.33 (28 tablets, tariff 4.20)<br>5.08 (28 tablets, tariff 4.20)<br>7.64 (28 tablets, tariff 4.20)<br>26.31 (100 tablets)<br>18.14(100 tablets)<br>111.89 (100 tablets)
na								0	
na								0	
Nitrofurantoin (7/7 course)	Nitrofurantoin 50mg course	4.20 (28 tablets, tariff 4.20)						4.20	Medicine A alt costs:<br>7.51 (28 tablets, tariff 4.20)<br>31.33 (28 tablets, tariff 4.20)<br>5.08 (28 tablets, tariff 4.20)<br>7.64 (28 tablets, tariff 4.20)<br>26.31 (100 tablets)<br>18.14(100 tablets)<br>111.89 (100 tablets)
na								0	
na								0	
Movicol<br>Paracetamol	Movicol 1 sachet<br>Paracetamol 1g oral	3.54 (20 sachets, tariff 4.38)	18 (500mg/ml 200ml, tariff 18)					21.54	Medicine A alt costs:<br>8.11 (30 sachets, 13.9g, tariff 6.09)<br>4.38 (30 sachets, 6.9g, tariff 4.38)<br>13.49 (50 sachets, 13.7g)<br>5.41 (20 sachets, 13.8g)<br>5.31 (30 sachets, 6.9g, tariff: 4.38)<br>Medicine B alt cost: none
na								0	
Diazepam<br>Codeine	Diazepam 2mg oral<br>Diclofenac 50mg oral<br>Codeine phosphate 60mg oral	0.49 (28 tablets, tariff: 0.71	7.58 (28 tablets, tariff 11.33)	1.71 200ml				9.78	Medicine A alt costs:<br>0.93 (28 tablets, tariff: 0.71)<br>0.74 (28 tablets, tariff: 0.71)<br>0.71 (28 tablets, tariff: 0.71)<br>3.88(28 tablets, tariff: 0.71)<br>1.1 (28 tablets, tariff: 0.71)<br>medicine B alt costs:<br>8.20 (28 capsules, tariff 8.20)<br>11.33 (28 capsules, tariff 11.33)<br>medicine C alt costs:<br>1.90 (tariff: 1.90)<br>1.73 (tariff: 1.90)<br>16.80 for 2000ml<br>9.90 for 2000ml

Codeine<br>Lansoprazole	Codeine phosphate 60mg oral<br>Lansoprazole 30mg oral	1.71 200ml	3.55 (28 tablets, tariff: 4.13)					5.26	medicine A alt costs:<br>1.90 (tariff: 1.90)<br>1.73 (tariff: 1.90)<br>16.80 for 2000ml<br>9.90 for 2000ml<br>medicine B alt costs:<br>1.90 (tariff: 1.90)<br>1.73 (tariff: 1.90)<br>16.80 for 2000ml<br>9.90 for 2000ml
Nitrofurantoin	Nitrofurantoin 50mg	4.20 (28 tablets, tariff 4.20)						4.20	Medicine A alt costs:<br>7.51 (28 tablets, tariff 4.20)<br>31.33 (28 tablets, tariff 4.20)<br>5.08 (28 tablets, tariff 4.20)<br>7.64 (28 tablets, tariff 4.20)<br>26.31 (100 tablets)<br>18.14(100 tablets)<br>111.89 (100 tablets)
Paracetamol<br>Codeine	Paracetamol 1g oral<br>Codeine phosphate 60mg oral	.13 (16 tablets 500mg)	1.71 200ml					1.84	Medicine A alt costs:<br>.93 (8 tablets)<br>.86 (12 tablets)<br>1.42 (14 tablets)<br>1.04 (16 tablets)<br>.76 (16 tablets)<br>.12 (16 tablets)<br>.27 (16 tablets)<br>.72 (16 tablets)<br>.18 (16 tablets)<br>.43 (16 tablets)<br>.26 (16 tablets)<br>.36 (16 tablets)<br>.34 (16 tablets)<br>.38 (16 tablets)<br>.17 (16 tablets)<br>.19 (16 tablets)<br>.31 (16 tablets)<br>.45 (16 tablets)<br>.67 (20 tablets)<br>.48 (20 tablets)<br>.54 (20 tablets)<br>.82 (30 tablets)<br>.15 (32 tablets tariff 0.76)<br>.62 (32 tablets tariff 0.76)<br>.2 (32 tablets tariff 0.76)<br>1.92 (32 tablets tariff 0.76)<br>.89 (32 tablets tariff 0.76)<br>.76 (32 tablets tariff 0.76)<br>1.44 (32 tablets tariff 0.76)<br>.33 (32 tablets tariff 0.76)<br>.68 (32 tablets tariff 0.76)<br>.52 (32 tablets tariff 0.76)<br>.49 (32 tablets tariff 0.76)<br>.29 (32 tablets tariff 0.76)<br>1.34 (100 tablets tariff 2.38)<br>1.95 (100 tablets tariff 2.38)<br>1.62 (100 tablets tariff 2.38)<br>3.5 (100 tablets tariff 2.38)<br>2.78 (100 tablets tariff 2.38)<br>2.38 (100 tablets tariff 2.38)<br>4.5 (100 tablets tariff 2.38)<br>3.05 (100 tablets tariff 2.38)<br>2.5 (100 tablets tariff 2.38)<br>2.17 (100 tablets tariff 2.38)<br>2.2 (100 tablets tariff 2.38)<br>1.84 (100 tablets tariff 2.38)<br>2.53 (100 tablets tariff 2.38)<br>.6 (100 tablets tariff 2.38)<br>2.78 (100 tablets tariff 2.38)

									3.25 (100 tablets tariff 2.38)<br>23.8 (1000 tablets)<br>medicine B alt costs:<br>1.90 (tariff: 1.90)<br>1.73 (tariff: 1.90)<br>16.80 for 2000ml<br>9.90 for 2000ml
TTO Ciprofloxacin	Ciprofloxacin 500mg oral course	0.92 (10 tablets, tariff 1.57)						0.92	medicine A alt cost:<br>2.19 (20 tablets, tariff 1.57)<br>2.18 (20 tablets, tariff 1.57)<br>10.62 (20 tablets, tariff 1.57)<br>1.05(20 tablets, tariff 1.57)<br>12.49(20 tablets, tariff 1.57)<br>27.29 (20 tablets)<br>1.84(20 tablets)<br>28.23(20 tablets)<br>3.14(20 tablets)<br>4.37(20 tablets)<br>4.9(20 tablets)<br>9.1 (100 tablets)
na								0	

IV fluids<br>Morphine<br>Paracetamol	Normal saline (fluids) IV<br>Morphine 10mg IV<br>Paracetamol 1g IV<br>Ondansetron 4mg IV	2.62 Sodium chloride 0.9% (1l bottle, tariff 3.96)	11.44 (10 ampoules, tariff cost: 11.45)	14.40 (12 vials)	10.00 for 10 ampules (tarriff cost: 18.70)			18.46	Medicine A alt cost:<br>4.12 1l bottle<br>24.72 6x 1lbottle<br>3.1 500ml bottle<br>37.2 12x500ml bottle<br>0.59 100ml bottle<br>11.78 20x 100ml bottle<br>2.62 1l bottle<br>26.2 10x 1l bottle<br>13.25 10x1l bottle<br>6.13 10x 250ml bottle<br>2.38 500ml bottle<br>23.80 10x 500ml bottle<br>13.25 500ml bottle<br>11.78 50ml bottle<br>Medicine B alternative costs:<br>11.45 (tariff cost: 11.45)<br>11.87 (tariff cost: 11.45)<br>14 (tariff cost: 11.45)<br>Medicine D alt costs:<br>37.11 for 5 ampules (tarriff: 29.97)<br>18.70 for 10 ampules (tarrif cost: 18.70)<br>28.47 for 5 ampules (tarriff: 29.97)<br>5.40 for 5 ampules (tarrif: 29.97)<br>29.20 for 5 ampules (tarrif: 29.97)<br>5.80 for 5 ampules (tarrif cost: 29.97)<br>18.70 for 10 ampules (tarrif cost: 18.70).<br>decision: cheapest per ampule<br>Medicine C alt costs (NHS indicative):<br>14.40 for 12 vials<br>24 for 20 vials<br>17.90 for 10 vials (Tarriff 12)

Oxygen<br>Oral Dexamethasone<br>IV Hartmann's<br>Paracetamol	Dexamethasone 6mg orally<br>Hartmann's IV fluids<br>Paracetamol 1g oral	1.94 (28 tablets of 20mg)	3.95	.13 (16 tablets 500mg)				6.02	Medicine A alt costs:<br>2.39 (28 tablets)<br>24 (28 tablets)<br>12 (50 tablets, tariff 3.46)<br>42.85(50 tablets, tariff 3.46)<br>29.95(50 tablets, tariff 3.46)<br>4.41(50 tablets, tariff 3.46)<br>5.46(50 tablets, tariff 3.46)<br>12.99 (100 tablets)<br>8.82 (100 tablets)<br>25 (100 tablets)<br>Medicine B no info available, used: https://www.medekit.com/drugs/intravenous-fluids/hartmanns-solution-500ml-500-mlt-6587/<br>Medicine C alt costs:<br>.93 (8 tablets)<br>.86 (12 tablets)<br>1.42 (14 tablets)<br>1.04 (16 tablets)<br>.76 (16 tablets)<br>.12 (16 tablets)<br>.27 (16 tablets)<br>.72 (16 tablets)<br>.18 (16 tablets)<br>.43 (16 tablets)<br>.26 (16 tablets)<br>.36 (16 tablets)<br>.34 (16 tablets)<br>.38 (16 tablets)<br>.17 (16 tablets)<br>.19 (16 tablets)<br>.31 (16 tablets)<br>.45 (16 tablets)<br>.67 (20 tablets)<br>.48 (20 tablets)<br>.54 (20 tablets)<br>.82 (30 tablets)<br>.15 (32 tablets tariff 0.76)<br>.62 (32 tablets tariff 0.76)<br>.2 (32 tablets tariff 0.76)<br>1.92 (32 tablets tariff 0.76)<br>.89 (32 tablets tariff 0.76)<br>.76 (32 tablets tariff 0.76)<br>1.44 (32 tablets tariff 0.76)<br>.33 (32 tablets tariff 0.76)<br>.68 (32 tablets tariff 0.76)<br>.52 (32 tablets tariff 0.76)<br>.49 (32 tablets tariff 0.76)<br>.29 (32 tablets tariff 0.76)<br>1.34 (100 tablets tariff 2.38)<br>1.95 (100 tablets tariff 2.38)<br>1.62 (100 tablets tariff 2.38)<br>3.5 (100 tablets tariff 2.38)<br>2.78 (100 tablets tariff 2.38)<br>2.38 (100 tablets tariff 2.38)<br>4.5 (100 tablets tariff 2.38)<br>3.05 (100 tablets tariff 2.38)<br>2.5 (100 tablets tariff 2.38)<br>2.17 (100 tablets tariff 2.38)<br>2.2 (100 tablets tariff 2.38)<br>1.84 (100 tablets tariff 2.38)<br>2.53 (100 tablets tariff 2.38)<br>.6 (100 tablets tariff 2.38)<br>2.78 (100 tablets tariff 2.38)<br>3.25 (100 tablets tariff 2.38)<br>23.8 (1000 tablets)
na								0	

Ondansetron<br>IV morphine<br>IV Hartmann's	Ondansetron 4mg IV<br>IV morphine 10mg IV x 2<br>IV Hartmann's (fluids)<br>Paracetamol 1g IV<br>Codeine phosphate 60mg oral	10.00 for 10 ampules (tarriff cost: 18.70)	11.44 (10 ampoules, tariff cost: 11.45)	3.95	14.40 (12 vials)	1.71 200ml		41.5	Medicine A alt costs:<br>37.11 for 5 ampules (tarriff: 29.97)<br>18.70 for 10 ampules (tarrif cost: 18.70)<br>28.47 for 5 ampules (tarriff: 29.97)<br>5.40 for 5 ampules (tarrif: 29.97)<br>29.20 for 5 ampules (tarrif: 29.97)<br>5.80 for 5 ampules (tarrif cost: 29.97)<br>18.70 for 10 ampules (tarrif cost: 18.70).<br>decision: cheapest per ampule<br>Medicine B alternative costs:<br>11.45 (tariff cost: 11.45)<br>11.87 (tariff cost: 11.45)<br>14 (tariff cost: 11.45)<br>Medicine A no info available, used: https://www.medekit.com/drugs/intravenous-fluids/hartmanns-solution-500ml-500-mlt-6587/<br>Medicine D alt costs (NHS indicative):<br>14.40 for 12 vials<br>24 for 20 vials<br>17.90 for 10 vials (Tarriff 12)<br>medicine E alt costs:<br>1.90 (tariff: 1.90)<br>1.73 (tariff: 1.90)<br>16.80 for 2000ml<br>9.90 for 2000ml
na								0	
IV fluids<br>Pantoprazole<br>IV antiemetic	Normal saline (IV infusion)<br>IV pantoprazole 40mg IV	2.62 Sodium chloride 0.9% (1l bottle, tariff 3.96)	5 (1 vial, tariff 5)					7.62	Medicine A alt cost:<br>4.12 1l bottle<br>24.72 6x 1lbottle<br>3.1 500ml bottle<br>37.2 12x500ml bottle<br>0.59 100ml bottle<br>11.78 20x 100ml bottle<br>2.62 1l bottle<br>26.2 10x 1l bottle<br>13.25 10x1l bottle<br>6.13 10x 250ml bottle<br>2.38 500ml bottle<br>23.80 10x 500ml bottle<br>13.25 500ml bottle<br>11.78 50ml bottle<br>medicine B alt costs:<br>22.50 (5 vials, tariff 22.50)<br>25.53 (5 vials, tariff 22.50)
na	Co-beneldopa 50mg oral	5.90 (100 tablets, tariff 5.90						5.90	Medicine A No alternate costs.

na								0	
na								0	
na								0	
na								0	
na								0	
na								0	
na	Paracetamol 1g oral	.13 (16 tablets 500mg)						.13	Medicine A alt costs:<br>.93 (8 tablets)<br>.86 (12 tablets)<br>1.42 (14 tablets)<br>1.04 (16 tablets)<br>.76 (16 tablets)<br>.12 (16 tablets)<br>.27 (16 tablets)<br>.72 (16 tablets)<br>.18 (16 tablets)<br>.43 (16 tablets)<br>.26 (16 tablets)<br>.36 (16 tablets)<br>.34 (16 tablets)<br>.38 (16 tablets)<br>.17 (16 tablets)<br>.19 (16 tablets)<br>.31 (16 tablets)<br>.45 (16 tablets)<br>.67 (20 tablets)<br>.48 (20 tablets)<br>.54 (20 tablets)<br>.82 (30 tablets)<br>.15 (32 tablets tariff 0.76)<br>.62 (32 tablets tariff 0.76)<br>.2 (32 tablets tariff 0.76)<br>1.92 (32 tablets tariff 0.76)<br>.89 (32 tablets tariff 0.76)<br>.76 (32 tablets tariff 0.76)<br>1.44 (32 tablets tariff 0.76)<br>.33 (32 tablets tariff 0.76)<br>.68 (32 tablets tariff 0.76)<br>.52 (32 tablets tariff 0.76)<br>.49 (32 tablets tariff 0.76)<br>.29 (32 tablets tariff 0.76)<br>1.34 (100 tablets tariff 2.38)<br>1.95 (100 tablets tariff 2.38)<br>1.62 (100 tablets tariff 2.38)<br>3.5 (100 tablets tariff 2.38)<br>2.78 (100 tablets tariff 2.38)<br>2.38 (100 tablets tariff 2.38)<br>4.5 (100 tablets tariff 2.38)<br>3.05 (100 tablets tariff 2.38)<br>2.5 (100 tablets tariff 2.38)<br>2.17 (100 tablets tariff 2.38)<br>2.2 (100 tablets tariff 2.38)<br>1.84 (100 tablets tariff 2.38)<br>2.53 (100 tablets tariff 2.38)<br>.6 (100 tablets tariff 2.38)<br>2.78 (100 tablets tariff 2.38)<br>3.25 (100 tablets tariff 2.38)<br>23.8 (1000 tablets)

Antiemetics	Paracetamol 1g IV<br>Ondansetron 4mg IV	14.40 (12 vials)	10.00 for 10 ampules (tarriff cost: 18.70)					24.40	Medicine A alt costs (NHS indicative):<br>14.40 for 12 vials<br>24 for 20 vials<br>17.90 for 10 vials (Tarriff 12)<br>Medicine B alt costs:<br>37.11 for 5 ampules (tarriff: 29.97)<br>18.70 for 10 ampules (tarrif cost: 18.70)<br>28.47 for 5 ampules (tarriff: 29.97)<br>5.40 for 5 ampules (tarrif: 29.97)<br>29.20 for 5 ampules (tarrif: 29.97)<br>5.80 for 5 ampules (tarrif cost: 29.97)<br>18.70 for 10 ampules (tarrif cost: 18.70).<br>decision: cheapest per ampule
TTO antibiotics	Amoxicillin 500mg oral course	0.41 (15 capsules, tariff 0.91)						0.41	Medicin A alt costs:<br>1.8 (15 capsules, tariff 0.91)<br>2.03 (21 capsules, tariff 1.27)<br>6.73 (100 capsules)<br>2.50 (21 capsules, tariff 1.27)<br>1.49 (15 capsules, tariff 0.91)<br>1.88 (21 capsules, tariff 1.27)<br>7.95 (100 capsules)<br>10.99 (21 capsules, tariff 1.27)<br>0.45 (21 capsules, tariff 1.27)<br>7.5 (15 capsules, tariff 0.91)<br>15 (21 capsules, tariff 1.27)<br>75 (100 capsules)<br>0.73 (21 capsules, tariff 1.27)<br>0.78 (21 capsules, tariff 1.27)

Antibiotics<br>Morphine<br>IV fluids	Augmentin 1.2g IV<br>Morphine 10mg IV<br>IV fluids (Normal saline)	10.60 (1000 + 200mg vial Amoxicillin)	11.44 (10 ampoules, tariff cost: 11.45)	2.62 Sodium chloride 0.9% (1l bottle, tariff 3.96)				24.66	medicine A Closest dosage to 1.2g IV; 1000mg vial Amoxicillin + 200mg patassium clavulanate. Alt costs:<br>29.70 (1000+200mg)<br>10.96 (1000+200mg)<br>50 (1000+200mg)<br>27.50 (1000+200mg)<br>Medicine B alternative costs:<br>11.45 (tariff cost: 11.45)<br>11.87 (tariff cost: 11.45)<br>14 (tariff cost: 11.45)<br>Medicine C alt cost:<br>4.12 1l bottle<br>24.72 6x 1lbottle<br>3.1 500ml bottle<br>37.2 12x500ml bottle<br>0.59 100ml bottle<br>11.78 20x 100ml bottle<br>2.62 1l bottle<br>26.2 10x 1l bottle<br>13.25 10x1l bottle<br>6.13 10x 250ml bottle<br>2.38 500ml bottle<br>23.80 10x 500ml bottle<br>13.25 500ml bottle<br>11.78 50ml bottle
na								0	
na								0	
na								0	

Codeine (60mg)<br>Paracetamol (1g)<br>Ibuprofen (400mg)	Codeine phosphate oral (60mg)<br>Paracetamol (1g) oral<br>Ibuprofen (400mg) oral	1.71 200ml	.13 (16 tablets 500mg)	0.56 (24 tablets, tariff price 1.03)				2.4	medicine A alt costs:<br>1.90 (tariff: 1.90)<br>1.73 (tariff: 1.90)<br>16.80 for 2000ml<br>9.90 for 2000ml<br>Medicine B alt costs:<br>.93 (8 tablets)<br>.86 (12 tablets)<br>1.42 (14 tablets)<br>1.04 (16 tablets)<br>.76 (16 tablets)<br>.12 (16 tablets)<br>.27 (16 tablets)<br>.72 (16 tablets)<br>.18 (16 tablets)<br>.43 (16 tablets)<br>.26 (16 tablets)<br>.36 (16 tablets)<br>.34 (16 tablets)<br>.38 (16 tablets)<br>.17 (16 tablets)<br>.19 (16 tablets)<br>.31 (16 tablets)<br>.45 (16 tablets)<br>.67 (20 tablets)<br>.48 (20 tablets)<br>.54 (20 tablets)<br>.82 (30 tablets)<br>.15 (32 tablets tariff 0.76)<br>.62 (32 tablets tariff 0.76)<br>.2 (32 tablets tariff 0.76)<br>1.92 (32 tablets tariff 0.76)<br>.89 (32 tablets tariff 0.76)<br>.76 (32 tablets tariff 0.76)<br>1.44 (32 tablets tariff 0.76)<br>.33 (32 tablets tariff 0.76)<br>.68 (32 tablets tariff 0.76)<br>.52 (32 tablets tariff 0.76)<br>.49 (32 tablets tariff 0.76)<br>.29 (32 tablets tariff 0.76)<br>1.34 (100 tablets tariff 2.38)<br>1.95 (100 tablets tariff 2.38)<br>1.62 (100 tablets tariff 2.38)<br>3.5 (100 tablets tariff 2.38)<br>2.78 (100 tablets tariff 2.38)<br>2.38 (100 tablets tariff 2.38)<br>4.5 (100 tablets tariff 2.38)<br>3.05 (100 tablets tariff 2.38)<br>2.5 (100 tablets tariff 2.38)<br>2.17 (100 tablets tariff 2.38)<br>2.2 (100 tablets tariff 2.38)<br>1.84 (100 tablets tariff 2.38)<br>2.53 (100 tablets tariff 2.38)<br>.6 (100 tablets tariff 2.38)<br>2.78 (100 tablets tariff 2.38)<br>3.25 (100 tablets tariff 2.38)<br>23.8 (1000 tablets)<br>Medicine C alt costs:<br>1.15 (48 tablets, tariff price 1.03)<br>2.05 (96 tablets, tariff price 1.03)<br>4.9 (60 tablets, 1.03 tariff)<br>2.06 (24 tablets, 1,03 tariff)<br>3.49 (48 tablets, 1.03 tariff)<br>5.49 (96 tablets, 1.03 tariff)<br>2.8 (84 tablets, 3.61 tariff)<br>1.39 (24 tablets, 1.03 tariff)<br>2.56 (48 tablets)<br>4.1 (84 tablets, 3.61 tariff)<br>1.03 (24 tablets, 1.03 tariff)<br>2.06 (48 tablets)<br>3.61 (84 tablets, 3.61 tariff)<br>6.14 (84 tablets, 3.61 tariff)<br>0.7 (24 tablets, 1.03 tariff)<br>0.85 (48 tablets)<br>1 (84 tablets, 3.61 tariff)<br>3 (96 tablets)<br>2.71, (84 tablets, 3.61 tariff)<br>5.74 (84 tablets, 3.61 tariff)<br>0.9 (24 tablets, 1.03 tariff)

									3.01 (84 tablets, 3.61 tariff)<br>0.7 (24 tablets, 1.03 tariff)<br>1.17 (48 tablets)<br>2 (96 tablets)
Codeine (30mg)	Codeine phosphate oral (30mg)	1.71 200ml						0	medicine A alt costs:<br>1.90 (tariff: 1.90)<br>1.73 (tariff: 1.90)<br>16.80 for 2000ml<br>9.90 for 2000ml

Urinary catheter<br>TTO Laxido	Unsure of cost of catheter ,<br>Laxido 1 sachet course<br>Phosphate eneme PR	0.99 (53cm catheter)	2.29 (20 sachets)	1.95 (tariff 1.95)				5.23	Medicine A alt cost:<br>1.19 (60cm)<br>medicine a information collected from: https://ukmedi.co.uk/22g-blue-1-inch-terumo-versatus-winged-and-ported-iv-cannula/?sku=DU2225PX-25&dfw_tracker=137014-600-0003&gclid=EAIaIQobChMI5Y6dha3k9gIViJftCh0Mxwe4EAQYASABEgLCx_D_BwE#/dffullscreen/query=catheter&query_name=match_and<br>Medicine B alt cost:<br>2.99 (30 sachets, 6.9g, tariff 4.38)<br>4.29 (30 sachets, tariff 6.09)<br>Medicine C alt cost:<br>30.78 (tariff 30.78)<br>27.93 (tariff 30.78)
na								0	
Stemetil	Prochlorperazine 20mg oral<br>Paracetamol 1g oral	3.34 (5mg/ml, 100ml)	.13 (16tablets, 500mg)					3.47	Medicine B no alt cost available<br>Medicine B alt costs:<br>.93 (8 tablets)<br>.86 (12 tablets)<br>1.42 (14 tablets)<br>1.04 (16 tablets)<br>.76 (16 tablets)<br>.12 (16 tablets)<br>.27 (16 tablets)<br>.72 (16 tablets)<br>.18 (16 tablets)<br>.43 (16 tablets)<br>.26 (16 tablets)<br>.36 (16 tablets)<br>.34 (16 tablets)<br>.38 (16 tablets)<br>.17 (16 tablets)<br>.19 (16 tablets)<br>.31 (16 tablets)<br>.45 (16 tablets)<br>.67 (20 tablets)<br>.48 (20 tablets)<br>.54 (20 tablets)<br>.82 (30 tablets)<br>.15 (32 tablets tariff 0.76)<br>.62 (32 tablets tariff 0.76)<br>.2 (32 tablets tariff 0.76)<br>1.92 (32 tablets tariff 0.76)<br>.89 (32 tablets tariff 0.76)<br>.76 (32 tablets tariff 0.76)<br>1.44 (32 tablets tariff 0.76)<br>.33 (32 tablets tariff 0.76)<br>.68 (32 tablets tariff 0.76)<br>.52 (32 tablets tariff 0.76)<br>.49 (32 tablets tariff 0.76)<br>.29 (32 tablets tariff 0.76)<br>1.34 (100 tablets tariff 2.38)<br>1.95 (100 tablets tariff 2.38)<br>1.62 (100 tablets tariff 2.38)<br>3.5 (100 tablets tariff 2.38)<br>2.78 (100 tablets tariff 2.38)<br>2.38 (100 tablets tariff 2.38)<br>4.5 (100 tablets tariff 2.38)<br>3.05 (100 tablets tariff 2.38)<br>2.5 (100 tablets tariff 2.38)<br>2.17 (100 tablets tariff 2.38)<br>2.2 (100 tablets tariff 2.38)

									1.84 (100 tablets tariff 2.38)<br>2.53 (100 tablets tariff 2.38)<br>.6 (100 tablets tariff 2.38)<br>2.78 (100 tablets tariff 2.38)<br>3.25 (100 tablets tariff 2.38)<br>23.8 (1000 tablets)

na	Paracetamol 1g oral course<br>Codeine phosphate 60mg course	.13 (16tablets 500mg)	1.71 200ml					1.84	Medicine A alt costs:<br>.93 (8 tablets)<br>.86 (12 tablets)<br>1.42 (14 tablets)<br>1.04 (16 tablets)<br>.76 (16 tablets)<br>.12 (16 tablets)<br>.27 (16 tablets)<br>.72 (16 tablets)<br>.18 (16 tablets)<br>.43 (16 tablets)<br>.26 (16 tablets)<br>.36 (16 tablets)<br>.34 (16 tablets)<br>.38 (16 tablets)<br>.17 (16 tablets)<br>.19 (16 tablets)<br>.31 (16 tablets)<br>.45 (16 tablets)<br>.67 (20 tablets)<br>.48 (20 tablets)<br>.54 (20 tablets)<br>.82 (30 tablets)<br>.15 (32 tablets tariff 0.76)<br>.62 (32 tablets tariff 0.76)<br>.2 (32 tablets tariff 0.76)<br>1.92 (32 tablets tariff 0.76)<br>.89 (32 tablets tariff 0.76)<br>.76 (32 tablets tariff 0.76)<br>1.44 (32 tablets tariff 0.76)<br>.33 (32 tablets tariff 0.76)<br>.68 (32 tablets tariff 0.76)<br>.52 (32 tablets tariff 0.76)<br>.49 (32 tablets tariff 0.76)<br>.29 (32 tablets tariff 0.76)<br>1.34 (100 tablets tariff 2.38)<br>1.95 (100 tablets tariff 2.38)<br>1.62 (100 tablets tariff 2.38)<br>3.5 (100 tablets tariff 2.38)<br>2.78 (100 tablets tariff 2.38)<br>2.38 (100 tablets tariff 2.38)<br>4.5 (100 tablets tariff 2.38)<br>3.05 (100 tablets tariff 2.38)<br>2.5 (100 tablets tariff 2.38)<br>2.17 (100 tablets tariff 2.38)<br>2.2 (100 tablets tariff 2.38)<br>1.84 (100 tablets tariff 2.38)<br>2.53 (100 tablets tariff 2.38)<br>.6 (100 tablets tariff 2.38)<br>2.78 (100 tablets tariff 2.38)<br>3.25 (100 tablets tariff 2.38)<br>23.8 (1000 tablets)<br>medicine B alt costs:<br>1.90 (tariff: 1.90)<br>1.73 (tariff: 1.90)<br>16.80 for 2000ml<br>9.90 for 2000ml
na								0	
na								0	
na								0	

na	Paracetamol 1g oral	.13 (16 tablets 500mg)						.13	Medicine A alt costs:<br>.93 (8 tablets)<br>.86 (12 tablets)<br>1.42 (14 tablets)<br>1.04 (16 tablets)<br>.76 (16 tablets)<br>.12 (16 tablets)<br>.27 (16 tablets)<br>.72 (16 tablets)<br>.18 (16 tablets)<br>.43 (16 tablets)<br>.26 (16 tablets)<br>.36 (16 tablets)<br>.34 (16 tablets)<br>.38 (16 tablets)<br>.17 (16 tablets)<br>.19 (16 tablets)<br>.31 (16 tablets)<br>.45 (16 tablets)<br>.67 (20 tablets)<br>.48 (20 tablets)<br>.54 (20 tablets)<br>.82 (30 tablets)<br>.15 (32 tablets tariff 0.76)<br>.62 (32 tablets tariff 0.76)<br>.2 (32 tablets tariff 0.76)<br>1.92 (32 tablets tariff 0.76)<br>.89 (32 tablets tariff 0.76)<br>.76 (32 tablets tariff 0.76)<br>1.44 (32 tablets tariff 0.76)<br>.33 (32 tablets tariff 0.76)<br>.68 (32 tablets tariff 0.76)<br>.52 (32 tablets tariff 0.76)<br>.49 (32 tablets tariff 0.76)<br>.29 (32 tablets tariff 0.76)<br>1.34 (100 tablets tariff 2.38)<br>1.95 (100 tablets tariff 2.38)<br>1.62 (100 tablets tariff 2.38)<br>3.5 (100 tablets tariff 2.38)<br>2.78 (100 tablets tariff 2.38)<br>2.38 (100 tablets tariff 2.38)<br>4.5 (100 tablets tariff 2.38)<br>3.05 (100 tablets tariff 2.38)<br>2.5 (100 tablets tariff 2.38)<br>2.17 (100 tablets tariff 2.38)<br>2.2 (100 tablets tariff 2.38)<br>1.84 (100 tablets tariff 2.38)<br>2.53 (100 tablets tariff 2.38)<br>.6 (100 tablets tariff 2.38)<br>2.78 (100 tablets tariff 2.38)<br>3.25 (100 tablets tariff 2.38)<br>23.8 (1000 tablets)
na								0	
na								0	

Codeine<br>Paracetamol	Paracetamol 1g oral<br>Codeine phosphate 60mg oral	13 (16 tablets 500mg)	1.71 200ml					1.84	Medicine A alt costs:<br>.93 (8 tablets)<br>.86 (12 tablets)<br>1.42 (14 tablets)<br>1.04 (16 tablets)<br>.76 (16 tablets)<br>.12 (16 tablets)<br>.27 (16 tablets)<br>.72 (16 tablets)<br>.18 (16 tablets)<br>.43 (16 tablets)<br>.26 (16 tablets)<br>.36 (16 tablets)<br>.34 (16 tablets)<br>.38 (16 tablets)<br>.17 (16 tablets)<br>.19 (16 tablets)<br>.31 (16 tablets)<br>.45 (16 tablets)<br>.67 (20 tablets)<br>.48 (20 tablets)<br>.54 (20 tablets)<br>.82 (30 tablets)<br>.15 (32 tablets tariff 0.76)<br>.62 (32 tablets tariff 0.76)<br>.2 (32 tablets tariff 0.76)<br>1.92 (32 tablets tariff 0.76)<br>.89 (32 tablets tariff 0.76)<br>.76 (32 tablets tariff 0.76)<br>1.44 (32 tablets tariff 0.76)<br>.33 (32 tablets tariff 0.76)<br>.68 (32 tablets tariff 0.76)<br>.52 (32 tablets tariff 0.76)<br>.49 (32 tablets tariff 0.76)<br>.29 (32 tablets tariff 0.76)<br>1.34 (100 tablets tariff 2.38)<br>1.95 (100 tablets tariff 2.38)<br>1.62 (100 tablets tariff 2.38)<br>3.5 (100 tablets tariff 2.38)<br>2.78 (100 tablets tariff 2.38)<br>2.38 (100 tablets tariff 2.38)<br>4.5 (100 tablets tariff 2.38)<br>3.05 (100 tablets tariff 2.38)<br>2.5 (100 tablets tariff 2.38)<br>2.17 (100 tablets tariff 2.38)<br>2.2 (100 tablets tariff 2.38)<br>1.84 (100 tablets tariff 2.38)<br>2.53 (100 tablets tariff 2.38)<br>.6 (100 tablets tariff 2.38)<br>2.78 (100 tablets tariff 2.38)<br>3.25 (100 tablets tariff 2.38)<br>23.8 (1000 tablets)<br>medicine B alt costs:<br>1.90 (tariff: 1.90)<br>1.73 (tariff: 1.90)<br>16.80 for 2000ml<br>9.90 for 2000ml
na								0	

na	Paracetamol 1g oral	.13 (16 tablets 500mg)						.13	Medicine A alt costs:<br>.93 (8 tablets)<br>.86 (12 tablets)<br>1.42 (14 tablets)<br>1.04 (16 tablets)<br>.76 (16 tablets)<br>.12 (16 tablets)<br>.27 (16 tablets)<br>.72 (16 tablets)<br>.18 (16 tablets)<br>.43 (16 tablets)<br>.26 (16 tablets)<br>.36 (16 tablets)<br>.34 (16 tablets)<br>.38 (16 tablets)<br>.17 (16 tablets)<br>.19 (16 tablets)<br>.31 (16 tablets)<br>.45 (16 tablets)<br>.67 (20 tablets)<br>.48 (20 tablets)<br>.54 (20 tablets)<br>.82 (30 tablets)<br>.15 (32 tablets tariff 0.76)<br>.62 (32 tablets tariff 0.76)<br>.2 (32 tablets tariff 0.76)<br>1.92 (32 tablets tariff 0.76)<br>.89 (32 tablets tariff 0.76)<br>.76 (32 tablets tariff 0.76)<br>1.44 (32 tablets tariff 0.76)<br>.33 (32 tablets tariff 0.76)<br>.68 (32 tablets tariff 0.76)<br>.52 (32 tablets tariff 0.76)<br>.49 (32 tablets tariff 0.76)<br>.29 (32 tablets tariff 0.76)<br>1.34 (100 tablets tariff 2.38)<br>1.95 (100 tablets tariff 2.38)<br>1.62 (100 tablets tariff 2.38)<br>3.5 (100 tablets tariff 2.38)<br>2.78 (100 tablets tariff 2.38)<br>2.38 (100 tablets tariff 2.38)<br>4.5 (100 tablets tariff 2.38)<br>3.05 (100 tablets tariff 2.38)<br>2.5 (100 tablets tariff 2.38)<br>2.17 (100 tablets tariff 2.38)<br>2.2 (100 tablets tariff 2.38)<br>1.84 (100 tablets tariff 2.38)<br>2.53 (100 tablets tariff 2.38)<br>.6 (100 tablets tariff 2.38)<br>2.78 (100 tablets tariff 2.38)<br>3.25 (100 tablets tariff 2.38)<br>23.8 (1000 tablets)

Ondansetron<br>Morphine	Ondansetron 4mg IV<br>Morphine 10mg IV<br>Codeine 60mg oral course	10.00 for 10 ampules (tarriff cost: 18.70)	11.44 (10 ampoules, tariff cost: 11.45)	1.71 200ml				23.15	Medicine A alt costs:<br>37.11 for 5 ampules (tarriff: 29.97)<br>18.70 for 10 ampules (tarrif cost: 18.70)<br>28.47 for 5 ampules (tarriff: 29.97)<br>5.40 for 5 ampules (tarrif: 29.97)<br>29.20 for 5 ampules (tarrif: 29.97)<br>5.80 for 5 ampules (tarrif cost: 29.97)<br>18.70 for 10 ampules (tarrif cost: 18.70).<br>decision: cheapest per ampule<br>Medicine B alternative costs:<br>11.45 (tariff cost: 11.45)<br>11.87 (tariff cost: 11.45)<br>14 (tariff cost: 11.45)<br>medicine C alt costs:<br>1.90 (tariff: 1.90)<br>1.73 (tariff: 1.90)<br>16.80 for 2000ml<br>9.90 for 2000ml
Codeine	Codeine phosphate 60mg oral	1.71 200ml						1.71	medicine A alt costs:<br>1.90 (tariff: 1.90)<br>1.73 (tariff: 1.90)<br>16.80 for 2000ml<br>9.90 for 2000ml
na								0	

IV saline<br>IV paracetamol<br>IV ondansetron<br>IV morphine<br>Urine dip	IV Normal Saline (fluids)<br>IV paracetamol 1g<br>IV ondansetron (4mg)<br>IV morphine (10mg)<br>Urine dip (approx 10p)	2.62 Sodium chloride 0.9% (1l bottle, tariff 3.96)	14.40 (12 vials)	10.00 for 10 ampules (tarriff cost: 18.70)	11.44 (10 ampoules, tariff cost: 11.45)	2,99 (100 strips)		30.01	Medicine A alt cost:<br>4.12 1l bottle<br>24.72 6x 1lbottle<br>3.1 500ml bottle<br>37.2 12x500ml bottle<br>0.59 100ml bottle<br>11.78 20x 100ml bottle<br>2.62 1l bottle<br>26.2 10x 1l bottle<br>13.25 10x1l bottle<br>6.13 10x 250ml bottle<br>2.38 500ml bottle<br>23.80 10x 500ml bottle<br>13.25 500ml bottle<br>11.78 50ml bottle<br>Medicine B alt costs (NHS indicative):<br>14.40 for 12 vials<br>24 for 20 vials<br>17.90 for 10 vials (Tarriff 12)<br>Medicine C alt costs:<br>37.11 for 5 ampules (tarriff: 29.97)<br>18.70 for 10 ampules (tarrif cost: 18.70)<br>28.47 for 5 ampules (tarriff: 29.97)<br>5.40 for 5 ampules (tarrif: 29.97)<br>29.20 for 5 ampules (tarrif: 29.97)<br>5.80 for 5 ampules (tarrif cost: 29.97)<br>18.70 for 10 ampules (tarrif cost: 18.70).<br>decision: cheapest per ampule<br>Medicine D alternative costs:<br>11.45 (tariff cost: 11.45)<br>Medicine E alternative costs:<br>6.99 (100)<br>7.95 (100)<br>8.99 (100)<br>collected from: https://www.valuemed.co.uk/collections/urine-testing-strips-uk<br>11.87 (tariff cost: 11.45)<br>14 (tariff cost: 11.45)
Peptac<br>Oral co-codamol	Peptac oral<br>Paracetamol and dihydrocodeine oral	1.95 (500ml, tariff 1.95)	2.49 (30 tablets, tariff 1.69)					4.34	Medicine A alt prices: none<br>10mg/500mg tablets most common Medicine B alt costs:<br>2.50 (30 tablets, tariff 1.69)<br>3.24(30 tablets, tariff 1.69)<br>4.50 (100 tablets, tariff 5.63)<br>7.59(100 tablets, tariff 5.63)<br>8.30(100 tablets, tariff 5.63)<br>9.75(100 tablets, tariff 5.63)<br>28.15 (500 tablets
na								0	

Oral paracetamol	Paracetamol 1g oral	.13 (16 tablets 500mg)						.13	Medicine A alt costs:<br>.93 (8 tablets)<br>.86 (12 tablets)<br>1.42 (14 tablets)<br>1.04 (16 tablets)<br>.76 (16 tablets)<br>.12 (16 tablets)<br>.27 (16 tablets)<br>.72 (16 tablets)<br>.18 (16 tablets)<br>.43 (16 tablets)<br>.26 (16 tablets)<br>.36 (16 tablets)<br>.34 (16 tablets)<br>.38 (16 tablets)<br>.17 (16 tablets)<br>.19 (16 tablets)<br>.31 (16 tablets)<br>.45 (16 tablets)<br>.67 (20 tablets)<br>.48 (20 tablets)<br>.54 (20 tablets)<br>.82 (30 tablets)<br>.15 (32 tablets tariff 0.76)<br>.62 (32 tablets tariff 0.76)<br>.2 (32 tablets tariff 0.76)<br>1.92 (32 tablets tariff 0.76)<br>.89 (32 tablets tariff 0.76)<br>.76 (32 tablets tariff 0.76)<br>1.44 (32 tablets tariff 0.76)<br>.33 (32 tablets tariff 0.76)<br>.68 (32 tablets tariff 0.76)<br>.52 (32 tablets tariff 0.76)<br>.49 (32 tablets tariff 0.76)<br>.29 (32 tablets tariff 0.76)<br>1.34 (100 tablets tariff 2.38)<br>1.95 (100 tablets tariff 2.38)<br>1.62 (100 tablets tariff 2.38)<br>3.5 (100 tablets tariff 2.38)<br>2.78 (100 tablets tariff 2.38)<br>2.38 (100 tablets tariff 2.38)<br>4.5 (100 tablets tariff 2.38)<br>3.05 (100 tablets tariff 2.38)<br>2.5 (100 tablets tariff 2.38)<br>2.17 (100 tablets tariff 2.38)<br>2.2 (100 tablets tariff 2.38)<br>1.84 (100 tablets tariff 2.38)<br>2.53 (100 tablets tariff 2.38)<br>.6 (100 tablets tariff 2.38)<br>2.78 (100 tablets tariff 2.38)<br>3.25 (100 tablets tariff 2.38)<br>23.8 (1000 tablets)
na								0	

Salbutamol nebuliser<br>Oral prednisolone<br>Ipratropium bromide nebuliser<br>Doxycycline oral	Salbutamol nebuliser 5mg<br>Predinsolone 40mg oral<br>Ipatropium bromide 0.5mg nebuliser<br>doxycycline 200mg oral<br>Codeine phosphate 60mg oral	5.87 (20 unit doses, tariff 5.97)	19.45 (28tablets of 20mg, tariff: 19.45)	2.73 (20 doses, tariff: 2.73)	0.9 (8 tablets of 100mg, tariff 0.9)	1.71 200ml		30.62	Medicine A alt costs:<br>5.97 (20 doses, tariff 5.97)<br>6.24 (20 doses, tariff 5.97)medicine B Closest dosage to 40mg available.<br>alt costs: none.<br>medicine C alt cost;<br>4.87 (20 doses, tariff 2.73)<br>15.99 (20 doses, tariff 2.73)<br>3.58 (20 doses, tariff 2.73)<br>3.04 (20 doses, tariff 2.73)<br>Medicine D tablets of 100mg closest dosage. alt costs:<br>1.05 (8 tablets, tariff 0.9)<br>6.56 (50 tablets)<br>1.04 (8 tablets, tariff 0.9)<br>6.55 (50 tablets)<br>5.63 (50 tablets)<br>3 (8 tablets, tariff 0.9)<br>5.25 (14 tablets)<br>19 (50 tablets)<br>2.25 (8tablets, tariff 0.9)<br>medicine E alt costs:<br>1.90 (tariff: 1.90)<br>1.73 (tariff: 1.90)<br>16.80 for 2000ml<br>9.90 for 2000ml
na								0	
na								0	
IV Hartmann's	Paracetamol 1g IV<br>Paracetamol 1g oral<br>Morphine 10mg IV<br>Hartmanns IV fluid	14.40 (12 vials)	18 (500mg/ml 200ml, tariff 18)	11.44 (10 ampoules, tariff cost: 11.45)	3.95			44.79	Medicine A alt costs (NHS indicative):<br>14.40 for 12 vials<br>24 for 20 vials<br>17.90 for 10 vials (Tarriff 12)<br>Medicine B alt cost: none<br>Medicine C alternative costs:<br>11.45 (tariff cost: 11.45)<br>11.87 (tariff cost: 11.45)<br>14 (tariff cost: 11.45)<br>Medicine C no info available, used:<br>https://www.medekit.com/drugs/intravenous-fluids/hartmanns-solution-500ml-500-mlt-6587/

IV Hartmann's<br>IV Amoxicillin<br>IV ceftriaxone<br>IV ondansetron<br>Oxygen<br>IV paracetamol	IV Hartmanns fluid<br>IV Amoxicillin 2g<br>IV ceftriaxone 2g<br>IV ondansetron 4mg<br>Oxygen<br>IV paracetamol 1g	3.95	1.92 (10x 1g vials powder for iv, tariff 1.92)	18.3 (1 vial, tariff 19.18)	10.00 for 10 ampules (tarriff cost: 18.70)	8.96 (340l)	14.40 (12 vials)	57.53	Medicine A no info available, used: https://www.medekit.com/drugs/intravenous-fluids/hartmanns-solution-500ml-500-mlt-6587/<br>Medicine B alt cost:<br>16.50 (10 x 1g vials)<br>Medicine C alt cost:<br>19.18 (1 vial, tariff: 19.18)<br>191.8 (10 vials)<br>122 (10vials)<br>Medicine D alt costs:<br>37.11 for 5 ampules (tarriff: 29.97)<br>18.70 for 10 ampules (tarrif cost: 18.70)<br>28.47 for 5 ampules (tarriff: 29.97)<br>5.40 for 5 ampules (tarrif: 29.97)<br>29.20 for 5 ampules (tarrif: 29.97)<br>5.80 for 5 ampules (tarrif cost: 29.97)<br>18.70 for 10 ampules (tarrif cost: 18.70).<br>decision: cheapest per ampule<br>medicine E alternative cost:<br>17.77 (300l)<br>12 (460l)<br>21.62 (600l)<br>9.77 (680l)<br>11.39 (1360l)<br>12.23 (1360l)<br>17.23 (2300l)<br>15.31 (3400l)<br>24.04 (3040l)<br>21.62 (6800l)<br>37.61 (11300l) information gathered from: https://www.boconline.co.uk/shop/en/uk/gas-a-z/oxygen/oxygen-cylinder-medical-grade-compressed-gas<br>Medicine F alt costs (NHS indicative):<br>14.40 for 12 vials<br>24 for 20 vials<br>17.90 for 10 vials (Tarriff 12)
na								0	
na								0	
na								0	
na								0	
na								0	
na								0	
Naproxen<br>Diazepam	Narpoxen 50mg oral<br>Diazepam 2mg oral	45 (100ml, tariff 45.01)	0.49 (28 tablets, tariff: 0.71					45.49	medicine A alt cost:<br>45.01 (100ml, tariff 45.01)Medicine B alt costs:<br>0.93 (28 tablets, tariff: 0.71)<br>0.74 (28 tablets, tariff: 0.71)<br>0.71 (28 tablets, tariff: 0.71)<br>3.88(28 tablets, tariff: 0.71)<br>1.1 (28 tablets, tariff: 0.71)
na								0	

na	Omeprazole oral 40mg course<br>Naproxen oral 50mg course<br>Paracetamol oral 1g course<br>Codeine Phosphate 60mg course	.66 (7 capsules, tariff .7)	45 (100ml, tariff 45.01)	.13 (16 tablets 500mg)	1.71 200ml			47.5	medicine A alt costs:<br>8.35 (7 capsules, tariff .7)<br>.8(7 capsules, tariff .7)<br>.7(7 capsules, tariff .7)<br>4.93(7 capsules, tariff .7)<br>.66(7 capsules, tariff .7)<br>2.37(7 capsules, tariff .7)<br>9.69(7 capsules, tariff .7)<br>3 (28 capsules)<br>9 (28 capsules)<br>9.48 (28 capsules)<br>2.64 (28 capsules)<br>3.2 (28 capsules)<br>19.72 (28 capsules)<br>26.72 (28 capsules)<br>2.8 (28 capsules)<br>3.19 (28 capsules)<br>medicine B alt cost:<br>45.01 (100ml, tariff 45.01)<br>Medicine C alt costs:<br>.93 (8 tablets)<br>.86 (12 tablets)<br>1.42 (14 tablets)<br>1.04 (16 tablets)<br>.76 (16 tablets)<br>.12 (16 tablets)<br>.27 (16 tablets)<br>.72 (16 tablets)<br>.18 (16 tablets)<br>.43 (16 tablets)<br>.26 (16 tablets)<br>.36 (16 tablets)<br>.34 (16 tablets)<br>.38 (16 tablets)<br>.17 (16 tablets)<br>.19 (16 tablets)<br>.31 (16 tablets)<br>.45 (16 tablets)<br>.67 (20 tablets)<br>.48 (20 tablets)<br>.54 (20 tablets)<br>.82 (30 tablets)<br>.15 (32 tablets tariff 0.76)<br>.62 (32 tablets tariff 0.76)<br>.2 (32 tablets tariff 0.76)<br>1.92 (32 tablets tariff 0.76)<br>.89 (32 tablets tariff 0.76)<br>.76 (32 tablets tariff 0.76)<br>1.44 (32 tablets tariff 0.76)<br>.33 (32 tablets tariff 0.76)<br>.68 (32 tablets tariff 0.76)<br>.52 (32 tablets tariff 0.76)<br>.49 (32 tablets tariff 0.76)<br>.29 (32 tablets tariff 0.76)<br>1.34 (100 tablets tariff 2.38)<br>1.95 (100 tablets tariff 2.38)<br>1.62 (100 tablets tariff 2.38)<br>3.5 (100 tablets tariff 2.38)<br>2.78 (100 tablets tariff 2.38)<br>2.38 (100 tablets tariff 2.38)<br>4.5 (100 tablets tariff 2.38)<br>3.05 (100 tablets tariff 2.38)<br>2.5 (100 tablets tariff 2.38)<br>2.17 (100 tablets tariff 2.38)<br>2.2 (100 tablets tariff 2.38)<br>1.84 (100 tablets tariff 2.38)<br>2.53 (100 tablets tariff 2.38)<br>.6 (100 tablets tariff 2.38)<br>2.78 (100 tablets tariff 2.38)<br>3.25 (100 tablets tariff 2.38)<br>23.8 (1000 tablets)<br>medicine D alt costs:<br>1.90 (tariff: 1.90)<br>1.73 (tariff: 1.90)<br>16.80 for 2000ml<br>9.90 for 2000ml

Codeine<br>Naproxen	Codeine phosphate 60mg oral<br>Naproxen 50mg oral	1.71 200ml	45 (100ml, tariff 45.01)					46.71	medicine A alt costs:<br>1.90 (tariff: 1.90)<br>1.73 (tariff: 1.90)<br>16.80 for 2000ml<br>9.90 for 2000ml<br>medicine B alt cost:<br>45.01 (100ml, tariff 45.01)
Codeine<br>Paracetamol	Codeine phosphate 60mg oral<br>Paracetamol 1g oral	1.71 200ml	.13 (16 tablets 500mg)					1.84	medicine A alt costs:<br>1.90 (tariff: 1.90)<br>1.73 (tariff: 1.90)<br>16.80 for 2000ml<br>9.90 for 2000ml<br>Medicine B alt costs:<br>.93 (8 tablets)<br>.86 (12 tablets)<br>1.42 (14 tablets)<br>1.04 (16 tablets)<br>.76 (16 tablets)<br>.12 (16 tablets)<br>.27 (16 tablets)<br>.72 (16 tablets)<br>.18 (16 tablets)<br>.43 (16 tablets)<br>.26 (16 tablets)<br>.36 (16 tablets)<br>.34 (16 tablets)<br>.38 (16 tablets)<br>.17 (16 tablets)<br>.19 (16 tablets)<br>.31 (16 tablets)<br>.45 (16 tablets)<br>.67 (20 tablets)<br>.48 (20 tablets)<br>.54 (20 tablets)<br>.82 (30 tablets)<br>.15 (32 tablets tariff 0.76)<br>.62 (32 tablets tariff 0.76)<br>.2 (32 tablets tariff 0.76)<br>1.92 (32 tablets tariff 0.76)<br>.89 (32 tablets tariff 0.76)<br>.76 (32 tablets tariff 0.76)<br>1.44 (32 tablets tariff 0.76)<br>.33 (32 tablets tariff 0.76)<br>.68 (32 tablets tariff 0.76)<br>.52 (32 tablets tariff 0.76)<br>.49 (32 tablets tariff 0.76)<br>.29 (32 tablets tariff 0.76)<br>1.34 (100 tablets tariff 2.38)<br>1.95 (100 tablets tariff 2.38)<br>1.62 (100 tablets tariff 2.38)<br>3.5 (100 tablets tariff 2.38)<br>2.78 (100 tablets tariff 2.38)<br>2.38 (100 tablets tariff 2.38)<br>4.5 (100 tablets tariff 2.38)<br>3.05 (100 tablets tariff 2.38)<br>2.5 (100 tablets tariff 2.38)<br>2.17 (100 tablets tariff 2.38)<br>2.2 (100 tablets tariff 2.38)<br>1.84 (100 tablets tariff 2.38)<br>2.53 (100 tablets tariff 2.38)<br>.6 (100 tablets tariff 2.38)<br>2.78 (100 tablets tariff 2.38)<br>3.25 (100 tablets tariff 2.38)<br>23.8 (1000 tablets)
na								0	
na								0	
na								0	

na	Paracetamol 1g oral	.13 (16tablets 500mg)						.13	Medicine A alt costs:<br>.93 (8 tablets)<br>.86 (12 tablets)<br>1.42 (14 tablets)<br>1.04 (16 tablets)<br>.76 (16 tablets)<br>.12 (16 tablets)<br>.27 (16 tablets)<br>.72 (16 tablets)<br>.18 (16 tablets)<br>.43 (16 tablets)<br>.26 (16 tablets)<br>.36 (16 tablets)<br>.34 (16 tablets)<br>.38 (16 tablets)<br>.17 (16 tablets)<br>.19 (16 tablets)<br>.31 (16 tablets)<br>.45 (16 tablets)<br>.67 (20 tablets)<br>.48 (20 tablets)<br>.54 (20 tablets)<br>.82 (30 tablets)<br>.15 (32 tablets tariff 0.76)<br>.62 (32 tablets tariff 0.76)<br>.2 (32 tablets tariff 0.76)<br>1.92 (32 tablets tariff 0.76)<br>.89 (32 tablets tariff 0.76)<br>.76 (32 tablets tariff 0.76)<br>1.44 (32 tablets tariff 0.76)<br>.33 (32 tablets tariff 0.76)<br>.68 (32 tablets tariff 0.76)<br>.52 (32 tablets tariff 0.76)<br>.49 (32 tablets tariff 0.76)<br>.29 (32 tablets tariff 0.76)<br>1.34 (100 tablets tariff 2.38)<br>1.95 (100 tablets tariff 2.38)<br>1.62 (100 tablets tariff 2.38)<br>3.5 (100 tablets tariff 2.38)<br>2.78 (100 tablets tariff 2.38)<br>2.38 (100 tablets tariff 2.38)<br>4.5 (100 tablets tariff 2.38)<br>3.05 (100 tablets tariff 2.38)<br>2.5 (100 tablets tariff 2.38)<br>2.17 (100 tablets tariff 2.38)<br>2.2 (100 tablets tariff 2.38)<br>1.84 (100 tablets tariff 2.38)<br>2.53 (100 tablets tariff 2.38)<br>.6 (100 tablets tariff 2.38)<br>2.78 (100 tablets tariff 2.38)<br>3.25 (100 tablets tariff 2.38)<br>23.8 (1000 tablets)
Codeine	Codeine phosphate 60mg oral	1.71 200ml						1.71	medicine A alt costs:<br>1.90 (tariff: 1.90)<br>1.73 (tariff: 1.90)<br>16.80 for 2000ml<br>9.90 for 2000ml
na								0	
na								0	
Codeine	Codeine phosphate 60mg oral	1.71 200ml						1.71	medicine A alt costs:<br>1.90 (tariff: 1.90)<br>1.73 (tariff: 1.90)<br>16.80 for 2000ml<br>9.90 for 2000ml
Salbutamol	Salbutamol nebuliser 5mg	5.87 (20 unit doses, tariff 5.97)						5.87	Medicine A alt costs:<br>5.97 (20 doses, tariff 5.97)<br>6.24 (20 doses, tariff 5.97)
na								0	

Codeine	Codeine phosphate 60mg oral	1.71 200ml						1.71	medicine A alt costs:<br>1.90 (tariff: 1.90)<br>1.73 (tariff: 1.90)<br>16.80 for 2000ml<br>9.90 for 2000ml
Codeine<br>Naproxen	Codeine phosphate 60mg oral<br>Naproxen 50mg oral	1.71 200ml	45 (100ml, tariff 45.01)					46.71	medicine A alt costs:<br>1.90 (tariff: 1.90)<br>1.73 (tariff: 1.90)<br>16.80 for 2000ml<br>9.90 for 2000ml<br>medicine B alt cost:<br>45.01 (100ml, tariff 45.01)
na								0	
na								0	
Paracetamol<br>Codeine	Codeine phosphate 60mg oral<br>Paracetamol 1g oral	1.71 200ml	.13 (16 tablets 500mg)					1.84	medicine A alt costs:<br>1.90 (tariff: 1.90)<br>1.73 (tariff: 1.90)<br>16.80 for 2000ml<br>9.90 for 2000ml<br>Medicine B alt costs:<br>.93 (8 tablets)<br>.86 (12 tablets)<br>1.42 (14 tablets)<br>1.04 (16 tablets)<br>.76 (16 tablets)<br>.12 (16 tablets)<br>.27 (16 tablets)<br>.72 (16 tablets)<br>.18 (16 tablets)<br>.43 (16 tablets)<br>.26 (16 tablets)<br>.36 (16 tablets)<br>.34 (16 tablets)<br>.38 (16 tablets)<br>.17 (16 tablets)<br>.19 (16 tablets)<br>.31 (16 tablets)<br>.45 (16 tablets)<br>.67 (20 tablets)<br>.48 (20 tablets)<br>.54 (20 tablets)<br>.82 (30 tablets)<br>.15 (32 tablets tariff 0.76)<br>.62 (32 tablets tariff 0.76)<br>.2 (32 tablets tariff 0.76)<br>1.92 (32 tablets tariff 0.76)<br>.89 (32 tablets tariff 0.76)<br>.76 (32 tablets tariff 0.76)<br>1.44 (32 tablets tariff 0.76)<br>.33 (32 tablets tariff 0.76)<br>.68 (32 tablets tariff 0.76)<br>.52 (32 tablets tariff 0.76)<br>.49 (32 tablets tariff 0.76)<br>.29 (32 tablets tariff 0.76)<br>1.34 (100 tablets tariff 2.38)<br>1.95 (100 tablets tariff 2.38)<br>1.62 (100 tablets tariff 2.38)<br>3.5 (100 tablets tariff 2.38)<br>2.78 (100 tablets tariff 2.38)<br>2.38 (100 tablets tariff 2.38)<br>4.5 (100 tablets tariff 2.38)<br>3.05 (100 tablets tariff 2.38)<br>2.5 (100 tablets tariff 2.38)<br>2.17 (100 tablets tariff 2.38)<br>2.2 (100 tablets tariff 2.38)<br>1.84 (100 tablets tariff 2.38)<br>2.53 (100 tablets tariff 2.38)<br>.6 (100 tablets tariff 2.38)<br>2.78 (100 tablets tariff 2.38)<br>3.25 (100 tablets tariff 2.38)<br>23.8 (1000 tablets)

Gaviscon<br>Paracetamol	Gaviscon oral<br>Paracetamol 1g oral	2.58 (150ml, tariff 2.58)	.13 (16 tablets 500mg)					2.71	Medicine A alt cost:<br>4.33 (300ml tariff 4.33)<br>7.11 (600ml tariff 7.11)<br>Medicine B alt costs:<br>.93 (8 tablets)<br>.86 (12 tablets)<br>1.42 (14 tablets)<br>1.04 (16 tablets)<br>.76 (16 tablets)<br>.12 (16 tablets)<br>.27 (16 tablets)<br>.72 (16 tablets)<br>.18 (16 tablets)<br>.43 (16 tablets)<br>.26 (16 tablets)<br>.36 (16 tablets)<br>.34 (16 tablets)<br>.38 (16 tablets)<br>.17 (16 tablets)<br>.19 (16 tablets)<br>.31 (16 tablets)<br>.45 (16 tablets)<br>.67 (20 tablets)<br>.48 (20 tablets)<br>.54 (20 tablets)<br>.82 (30 tablets)<br>.15 (32 tablets tariff 0.76)<br>.62 (32 tablets tariff 0.76)<br>.2 (32 tablets tariff 0.76)<br>1.92 (32 tablets tariff 0.76)<br>.89 (32 tablets tariff 0.76)<br>.76 (32 tablets tariff 0.76)<br>1.44 (32 tablets tariff 0.76)<br>.33 (32 tablets tariff 0.76)<br>.68 (32 tablets tariff 0.76)<br>.52 (32 tablets tariff 0.76)<br>.49 (32 tablets tariff 0.76)<br>.29 (32 tablets tariff 0.76)<br>1.34 (100 tablets tariff 2.38)<br>1.95 (100 tablets tariff 2.38)<br>1.62 (100 tablets tariff 2.38)<br>3.5 (100 tablets tariff 2.38)<br>2.78 (100 tablets tariff 2.38)<br>2.38 (100 tablets tariff 2.38)<br>4.5 (100 tablets tariff 2.38)<br>3.05 (100 tablets tariff 2.38)<br>2.5 (100 tablets tariff 2.38)<br>2.17 (100 tablets tariff 2.38)<br>2.2 (100 tablets tariff 2.38)<br>1.84 (100 tablets tariff 2.38)<br>2.53 (100 tablets tariff 2.38)<br>.6 (100 tablets tariff 2.38)<br>2.78 (100 tablets tariff 2.38)<br>3.25 (100 tablets tariff 2.38)<br>23.8 (1000 tablets)
na								0	

Morphine<br>Gentamicin<br>IV fluid	Morphine 10mg IV<br>Gentamycin 500mg IV<br>Normal Saline IV fluids	11.44 (10 ampoules, tariff cost: 11.45)	195.31	2.62 Sodium chloride 0.9% (1l bottle, tariff 3.96)				209.37	Medicine A alternative costs:<br>11.45 (tariff cost: 11.45)<br>11.87 (tariff cost: 11.45)<br>14 (tariff cost: 11.45)<br>medicine B: 20 bottles, 360mg IV the closest dosage. alt costs: none<br>Medicine C alt cost:<br>4.12 1l bottle<br>24.72 6x 1lbottle<br>3.1 500ml bottle<br>37.2 12x500ml bottle<br>0.59 100ml bottle<br>11.78 20x 100ml bottle<br>2.62 1l bottle<br>26.2 10x 1l bottle<br>13.25 10x1l bottle<br>6.13 10x 250ml bottle<br>2.38 500ml bottle<br>23.80 10x 500ml bottle<br>13.25 500ml bottle<br>11.78 50ml bottle
na								0	
Clarithromycin	Clarithromycin 500mg oral course	1.97 (14 tablets, tariff 1.7)						1.97	Medicine A alt cost:<br>2.19(14 tablets, tariff: 1.7)<br>2.2 (14 tablets, tariff: 1.7)<br>2.21 (14 tablets, tariff: 1.7)<br>21.50(14 tablets, tariff: 1.7)<br>2.65 (14 tablets, tariff: 1.7)
na								0	
na								0	
na	Morphine 10mg IV	11.44 (10 ampoules, tariff cost: 11.45)						11.44	Medicine A alternative costs:<br>11.45 (tariff cost: 11.45)<br>11.87 (tariff cost: 11.45)<br>14 (tariff cost: 11.45)
Aspirin<br>Fondaparinux<br>Clopidogrel	Aspirin 300mg oral<br>Fondaparinux (subcutaneous)<br>Clopidogrel 300mg oral	0.23 (32 tablets, tariff 3.88)	62.79 (10 tablets, 2.5mg tariff 62.79)	142.54 (30 tablets) tariff cost: 142.54				205.56	alternative costs medicine A:<br>3.54 (28 tablets)<br>12.54 (100 tablets, tariff 12.11)<br>3.88 (32 tablets, tariff 3.88)<br>0.28 (32 tablets, tariff 3.88)<br>Medicin B: no alternative costs<br>Medicin C: no alt costs

Oral Asprin<br>Oral Ticagrelor<br>Oral Fondaparinox	Aspirin 300mg oral<br>Ticagrelor 180mg oral<br>Fondaparinux subcutaneous	0.23 (32 tablets, tariff 3.88)	54.6 (90mg, tariff price 54.6)	62.79 (10 tablets, 2.5mg tariff 62.79)				117.47	alternative costs medicine A:<br>3.54 (28 tablets)<br>12.54 (100 tablets, tariff 12.11)<br>3.88 (32 tablets, tariff 3.88)<br>0.28 (32 tablets, tariff 3.88)<br>medicine B: 180 not available, 90x2 is. No alternative costs.<br>Medicin C: no alternative costs
na								0	
na								0	
na								0	
TTO clarithromycin	Clarithromycin 500mg oral course	1.97 (14 tablets, tariff 1.7)						1.97	Medicine A alt cost:<br>2.19(14 tablets, tariff: 1.7)<br>2.2 (14 tablets, tariff: 1.7)<br>2.21 (14 tablets, tariff: 1.7)<br>21.50(14 tablets, tariff: 1.7)<br>2.65 (14 tablets, tariff: 1.7)
IV Hartmann's<br>IV Ondansetron<br>IV Pantoprazole<br>IV Cyclizine<br>IV Paracetamol	IV Hartmann's (fluids)<br>IV ondansetron 4mg<br>IV pantoprazole 80mg<br>IV cyclizine<br>50mg<br>IV paracetamol 1g	3.95	10.00 for 10 ampules (tarriff cost: 18.70)	5x2=10 (1 vial, tariff 5)	9.45 (5 ampoules, tariff 5.78	14.40 (12 vials)		47.8	Medicine A no info available, used:<br>https://www.medekit.com/drugs/intravenous-fluids/hartmanns-solution-500ml-500-mlt-6587/<br>Medicine B alt costs:<br>37.11 for 5 ampules (tarriff: 29.97)<br>18.70 for 10 ampules (tarrif cost: 18.70)<br>28.47 for 5 ampules (tarriff: 29.97)<br>5.40 for 5 ampules (tarrif: 29.97)<br>29.20 for 5 ampules (tarrif: 29.97)<br>5.80 for 5 ampules (tarrif cost: 29.97)<br>18.70 for 10 ampules (tarrif cost: 18.70).<br>decision: cheapest per ampule<br>medicine C alt cost:<br>22.50 (5 vials, tariff 22.50)<br>25.53 (5 vials, tariff 22.50)Medicine D alt costs:<br>35 (10 amopoules)<br>16.25 (5 ampoules, 5.78 tariff)<br>32.50 (10 ampoules)<br>11.67 (5 ampoules, tariff 5.78)<br>12.74 (10 ampoules)<br>17.34 (10 ampoules)<br>Medicine E alt costs (NHS indicative):<br>14.40 for 12 vials<br>24 for 20 vials<br>17.90 for 10 vials (Tarriff 12)
na								0	

Urine dip + urine pregnancy<br>IV Hartmann's<br>Oral Paracetamol<br>Oral/IV ondansetron	Urine dip (approx 10 pence)<br>pregnancy test (approx £1)<br>IV Hartmann's<br>(fluids)<br>Paracetamol 1g oral<br>Ondansetron 4mg oral and IV	2,99 (100 strips)	3.99 (1 test)	3.95	.13 (16 tablets 500mg)	10.00 for 10 ampules (tarriff cost: 18.70)		21.06	Medicine A alternative costs:<br>6.99 (100)<br>7.95 (100)<br>8.99 (100)<br>collected from:<br>https://www.valuemed.co.uk/collections/urine-testing-strips-uk<br>Medicine B alt costs:<br>14 (2 tests)<br>4.99 (2)<br>16.99 (3)<br>10.49 (2)<br>8.99 (2<br>10.99 (2)<br>5.99 (1)<br>35.99 (18)<br>7.99 (1)<br>10.99 (1)<br>9.99 (1)<br>14.99 (2)<br>10.89 (2)<br>information collected from<br>https://www.boots.com/baby-child/pregnancy-maternity/pregnancy-tests<br>Medicine C no info available, used:<br>https://www.medekit.com/drugs/intravenous-fluids/hartmanns-solution-500ml-500-mlt-6587/<br>Medicine D alt costs:<br>.93 (8 tablets)<br>.86 (12 tablets)<br>1.42 (14 tablets)<br>1.04 (16 tablets)<br>.76 (16 tablets)<br>.12 (16 tablets)<br>.27 (16 tablets)<br>.72 (16 tablets)<br>.18 (16 tablets)<br>.43 (16 tablets)<br>.26 (16 tablets)<br>.36 (16 tablets)<br>.34 (16 tablets)<br>.38 (16 tablets)<br>.17 (16 tablets)<br>.19 (16 tablets)<br>.31 (16 tablets)<br>.45 (16 tablets)<br>.67 (20 tablets)<br>.48 (20 tablets)<br>.54 (20 tablets)<br>.82 (30 tablets)<br>.15 (32 tablets tariff 0.76)<br>.62 (32 tablets tariff 0.76)<br>.2 (32 tablets tariff 0.76)<br>1.92 (32 tablets tariff 0.76)<br>.89 (32 tablets tariff 0.76)<br>.76 (32 tablets tariff 0.76)<br>1.44 (32 tablets tariff 0.76)<br>.33 (32 tablets tariff 0.76)<br>.68 (32 tablets tariff 0.76)<br>.52 (32 tablets tariff 0.76)<br>.49 (32 tablets tariff 0.76)<br>.29 (32 tablets tariff 0.76)<br>1.34 (100 tablets tariff 2.38)<br>1.95 (100 tablets tariff 2.38)<br>1.62 (100 tablets tariff 2.38)<br>3.5 (100 tablets tariff 2.38)<br>2.78 (100 tablets tariff 2.38)<br>2.38 (100 tablets tariff 2.38)<br>4.5 (100 tablets tariff 2.38)<br>3.05 (100 tablets tariff 2.38)<br>2.5 (100 tablets tariff 2.38)<br>2.17 (100 tablets tariff 2.38)<br>2.2 (100 tablets tariff 2.38)<br>1.84 (100 tablets tariff 2.38)<br>2.53 (100 tablets tariff 2.38)

									.6 (100 tablets tariff 2.38)<br>2.78 (100 tablets tariff 2.38)<br>3.25 (100 tablets tariff 2.38)<br>23.8 (1000 tablets)<br>Medicine E alt costs:<br>37.11 for 5 ampules (tarriff: 29.97)<br>18.70 for 10 ampules (tarrif cost: 18.70)<br>28.47 for 5 ampules (tarriff: 29.97)<br>5.40 for 5 ampules (tarrif: 29.97)<br>29.20 for 5 ampules (tarrif: 29.97)<br>5.80 for 5 ampules (tarrif cost: 29.97)<br>18.70 for 10 ampules (tarrif cost: 18.70).<br>decision: cheapest per ampule
Oral Co-amoxiclav	Oral augmentin 625mg course	9.60 (21 tablets, tariff 2.09)						9.6	Medicin A no alt costs
IV Haloperidol<br>IV Hartmann's<br>IV Morphine<br>IV Paracetamol	IV Haloperidol<br>IV Hartmann's (fluids)<br>IV Morphine 10mg<br>IV Paracetamol 1g	35 (5mg IV, 10 ampoules, tariff: 53.98)	3.95	11.44 (10 ampoules, tariff cost: 11.45)	14.40 (12 vials)			61.79	Medicine A alt cost:<br>52.50 (10 vials, tariff 53.98)<br>53.98 (10 vials, tariff 53.98)<br>Medicine B no info available, used:<br>https://www.medekit.com/drugs/intravenous-fluids/hartmanns-solution-500ml-500-mlt-6587/<br>Medicine C alternative costs:<br>11.45 (tariff cost: 11.45)<br>11.87 (tariff cost: 11.45)<br>14 (tariff cost: 11.45)<br>Medicine D alt costs (NHS indicative):<br>14.40 for 12 vials<br>24 for 20 vials<br>17.90 for 10 vials (Tarriff 12)
na								0	
na								0	
na								0	

Urine	Not sure code for this - urine test strips (sell for £10 for 100 on Amazone??)	2,99 (100 strips)						2.99	Medicine A alternative costs:<br>6.99 (100)<br>7.95 (100)<br>8.99 (100)<br>collected from:<br>https://www.valuemed.co.uk/collections/urine-testing-strips-uk
na								0	
na								0	
na								0	
na								0	
Analgesia	Oral paracetamol 1g	.13 (16tablets 500mg)						.13	Medicine A alt costs:<br>.93 (8 tablets)<br>.86 (12 tablets)<br>1.42 (14 tablets)<br>1.04 (16 tablets)<br>.76 (16 tablets)<br>.12 (16 tablets)<br>.27 (16 tablets)<br>.72 (16 tablets)<br>.18 (16 tablets)<br>.43 (16 tablets)<br>.26 (16 tablets)<br>.36 (16 tablets)<br>.34 (16 tablets)<br>.38 (16 tablets)<br>.17 (16 tablets)<br>.19 (16 tablets)<br>.31 (16 tablets)<br>.45 (16 tablets)<br>.67 (20 tablets)<br>.48 (20 tablets)<br>.54 (20 tablets)<br>.82 (30 tablets)<br>.15 (32 tablets tariff 0.76)<br>.62 (32 tablets tariff 0.76)<br>.2 (32 tablets tariff 0.76)<br>1.92 (32 tablets tariff 0.76)<br>.89 (32 tablets tariff 0.76)<br>.76 (32 tablets tariff 0.76)<br>1.44 (32 tablets tariff 0.76)<br>.33 (32 tablets tariff 0.76)<br>.68 (32 tablets tariff 0.76)<br>.52 (32 tablets tariff 0.76)<br>.49 (32 tablets tariff 0.76)<br>.29 (32 tablets tariff 0.76)<br>1.34 (100 tablets tariff 2.38)<br>1.95 (100 tablets tariff 2.38)<br>1.62 (100 tablets tariff 2.38)<br>3.5 (100 tablets tariff 2.38)<br>2.78 (100 tablets tariff 2.38)<br>2.38 (100 tablets tariff 2.38)<br>4.5 (100 tablets tariff 2.38)<br>3.05 (100 tablets tariff 2.38)<br>2.5 (100 tablets tariff 2.38)<br>2.17 (100 tablets tariff 2.38)<br>2.2 (100 tablets tariff 2.38)<br>1.84 (100 tablets tariff 2.38)<br>2.53 (100 tablets tariff 2.38)<br>.6 (100 tablets tariff 2.38)<br>2.78 (100 tablets tariff 2.38)<br>3.25 (100 tablets tariff 2.38)<br>23.8 (1000 tablets)
na								0	
na								0	
na								0	

Lansoprazole	Oral Lansoprazole 30mg	3.55 (28 tablets, tariff: 4.13)						3.55	Medicine A alt cost:<br>7.46 (28 tablets, tariff 4.13)<br>5.15 (28 tablets, tariff 4.13)<br>55 (28 tablets, tariff 4.13)
na								0	
na								0	
Codeine<br>Naproxen	Oral Codeine Phosphate 60mg<br>Oral Naproxen 500mg	1.71 200ml	1.19 (28 tablets, tariff 1.36)					2.9	medicine A alt costs:<br>1.90 (tariff: 1.90)<br>1.73 (tariff: 1.90)<br>16.80 for 2000ml<br>9.90 for 2000ml<br>medicine B alt costs:<br>8.56<br>37.86 (500 tablets)<br>1.36 (28 tablets, tariff: 1.36)<br>3.42 (28 tablets, tariff: 1.36)<br>1.19 (28 tablets, tariff: 1.36)<br>8.14 (28 tablets, tariff: 1.36)<br>7.27 (28 tablets, tariff: 1.36)
na								0	
na								0	
na								0	
na								0	
na								0	
na								0	
