# Supplementary material for: Is emergency doctors’ tolerance of clinical uncertainty on a novel measure associated with doctor well-being, healthcare resource use and patient outcomes?
Source: Emerg Med J. 2024 Nov 27;42(1):e213256. doi: 10.1136/emermed-2023-213256 (PMC11874457; doi:10.1136/emermed-2023-213256)
Supplement: online supplemental file 6 [file emermed-42-1-s006.pdf]

This supplementary appendix (Supplementary File 3) includes:

1. **A of all multi-level model outputs for specific tests outcomes (secondary analyses).**

UT associations with outcomes.

Model	β [95% CI]	*p*	R^2	ICC	AIC | BIC
	**Random intercepts**				
	**Logistic models (effect size = odds ratios)**				
	Ordered tests: Any test ordered (yes versus no)				
Model I	0.75 [0.40-1.41]	0.18	C = 18%<br>M = 14%	5%	498 | 525
Model II	0.72 [0.38-1.36]	0.16	C = 18%<br>M = 14%	5%	496 | 528
Model III	0.72 [0.38-1.35]	0.16	C = 21%<br>M = 17%	5%	491 | 530
Model IV	0.74 [0.40-1.36]	0.17	C = 21%<br>M = 18%	4%	493 | 541
	Ordered treatments: Any treatment ordered (yes versus no)				
Model I	0.88 [0.52-1.49]	0.32	C = 7%<br>M = 6%	0.1%	515 | 543
Model II	0.87 [0.52-1.45]	0.30	C = -<br>M = 9%	nil	508 | 539
Model III	0.90 [0.54-1.52]	0.35	C = -<br>M = 11%	nil	506 | 545
Model IV	0.93 [0.55-1.59]	0.40	C =<br>M = 14%	nil	500 | 548
	Ordered treatments or tests: Any treatment or test ordered (yes versus no)				
Model I	0.65 [0.29-1.47]	0.15	C = 22%<br>M = 11%	13%	451 | 478
Model II	0.64 [0.29-1.43]	0.14	C = 22%<br>M = 11%	12%	451 | 483
Model III	0.66 [0.30-1.46]	0.15	C = 23%<br>M = 13%	11%	448 | 488
Model IV	0.71 [0.35-1.45]	0.18	C = 23%<br>M = 18%	7%	446 | 494
	Ordered tests: X-Ray (yes versus no)				
Model I	0.77 [0.35-1.69]	0.26	C = 15%<br>M = 4%	12%	457 | 485

UT associations with outcomes.

Model	β [95% CI]	p	R^2	ICC	AIC | BIC
			**Random intercepts**		
Model II	0.74 [0.34-1.64]	0.23	C = 15%<br>M = 4%	12%	454 | 486
Model III	0.71 [0.32-1.60]	0.21	C = 19%<br>M = 8%	12%	448 | 487
Model IV	0.65 [0.31-1.40]	0.14	C = 19%<br>M = 12%	8%	454 | 517
			Ordered tests: CT scan (yes versus no)		
Model I	1.10 [0.41-2.97]	0.58	C = -<br>M = 90%	-	199 | 227
Model II	1.12 [0.41-3.03]	0.59	C = -<br>M = 90%	-	200 | 232
Model III	0.98 [0.35-2.77]	0.29	C = -<br>M = 90%	-	195 | 234
Model IV	1.29 [0.42-3.99]	0.66	C = -<br>M = 90%	-	197 | 260
			Ordered tests: US scan (yes versus no)<br>*Event rate too low for stable estimates*		
			Ordered tests: MRI scan (yes versus no)<br>*Event rate too low for stable estimates*		
			Ordered tests: Blood test (any) (yes versus no)		
Model I	0.82 [0.39-1.72]	0.30	C = 15%<br>M = 10%	6%	394 | 422
Model II	0.79 [0.38-1.66]	0.27	C = 16%<br>M = 11%	6%	392 | 424
Model III	0.75 [0.36-1.56]	0.22	C = 17%<br>M = 13%	4%	390 | 430
Model IV	0.67 [0.34-1.32]	0.12	C = 16%<br>M = 16%	0.9%	398 | 461
			Ordered tests: ECG (yes versus no)		
Model I	0.26 [0.07-0.90]	0.02	C = -<br>M = 18%	-	160 | 187
Model II	0.26 [0.07-0.91]	0.02	C = -<br>M = 19%	-	162 | 193
Model III	0.24 [0.07-0.85]	0.02	C = -<br>M = 22%	-	163 | 203
Model IV	0.24 [0.06-0.92]	0.02	C = -<br>M = 27%	-	173 | 237
			Admittance on initial attendance (yes versus no)		

UT associations with outcomes.

Model	β [95% CI]	*p*	R^2	ICC	AIC | BIC
			**Random intercepts**		
Model I	1.07 [0.56-2.04]	0.85	C = -<br>M = 41%	-	372 | 400
Model II	1.05 [0.53-2.09]	0.89	C = -<br>M = 47%	-	346 | 378
Model III	0.99 [0.49-1.99]	0.97	C = -<br>M = 49%	-	336 | 375
Model IV	0.98 [0.46-2.09]	0.97	C = -<br>M = 51%	-	343 | 407
			30-day re-attendance (yes versus no)		
Model I	0.95 [0.47-1.92]	0.89	C = -<br>M = 4%	-	320 | 348
Model II	0.97 [0.47-2.00]	0.94	C = -<br>M = 9%	-	312 | 344
Model III	0.95 [0.46-1.95]	0.88	C = -<br>M = 13%	-	311 | 351
Model IV	1.05 [0.48-2.28]	0.91	C = -<br>M = 13%	-	321 | 384
			30-day admittance upon discharge (yes versus no)		
Model I	0.77 [0.28-2.11]	0.61	C = -<br>M = 18%	-	191 | 219
Model II	0.79 [0.28-2.26]	0.66	C = -<br>M = 24%	-	185 | 217
Model III	0.80 [0.28-2.30]	0.67	C = -<br>M = 24%	-	189 | 229
Model IV	0.82 [0.28-2.37]	0.71	C = -<br>M = 31%	-	196 | 259
			24-hour stay length (yes versus no)		
Model I	0.91 [0.35-2.34]	0.43	C = -<br>M = 99%	-	196 | 224
Model II	0.91 [0.35-2.34]	0.42	C = -<br>M = 98%	-	198 | 230
Model III	0.97 [0.36-2.59]	0.48	C = -<br>M = 98%	-	198 | 238
Model IV	1.21 [0.41-3.64]	0.73	C = -<br>M = 98%	-	198 | 245
			30-day death upon discharge (yes versus no)		
			Event rate too low for stable estimates		
			**Linear models (effect size = unstandardised beta)**		

UT associations with outcomes.

Model	β [95% CI]	*p*	R^2	ICC	AIC | BIC
	**Random intercepts**				
	***Cost index v1: Estimated total cost of all ordered tests + treatments** (note to log outcomes, a constant of £182 was added - which = avg cost of attendance)*				
Model I log	-0.01 [-0.06-0.05]	0.37	C = 12%<br>M = 8%	4%	-162 | -130
Model II log	-0.01 [-0.06-0.04]	0.36	C = 13%<br>M = 9%	3%	-156 | -120
Model III log	-0.01 [-0.06-0.04]	0.31	C = 16%<br>M = 13%	3%	-150 | -106
Model IV log	-0.01 [-0.06-0.04]	0.32	C = 16%<br>M = 13%	4%	-130 | -79
	Cost index v2: Index v1 + estimated hospital stay length adjusted admission costs				
Model I log	0.02 [-0.24-0.28]	0.56	C = 17%<br>M = 16%	1%	1122 | 1153
Model II log	-0.002 [-0.24-0.24]	0.49	C = -<br>M = 23%	nil	1088 | 1123
Model III log	-0.005 [-0.24-0.23]	0.48	C = -<br>M = 27%	nil	1082 | 1126
Model IV log	-0.002 [-0.23-0.23]	0.48	C = -<br>M = 28%	nil	1094 | 1145
	Cost index v3: Index v2 + estimated 30-day re-attendance/return admission costs				
Model I log	-0.05 [-0.34-0.24]	0.37	C = 19%<br>M = 17%	2%	1182 | 1213
Model II log	-0.07 [-0.33-0.19]	0.29	C = 26%<br>M = 25%	0.4%	1141 | 1177
Model III log	-0.08 [-0.33-0.17]	0.28	C = -<br>M = 27%	nil	1146 | 1190
Model IV log	-0.07 [-0.32-0.18]	0.28	C = -<br>M = 28%	nil	1156 | 1208
